# Revision of the *Lima* clade (Miconia sect. Lima, Miconieae, Melastomataceae) of the Greater Antilles

**DOI:** 10.3897/phytokeys.72.9355

**Published:** 2016-10-13

**Authors:** Lucas C. Majure, Eldis R. Bécquer, Walter S. Judd

**Affiliations:** 1 Department of Research, Conservation and Collections, Desert Botanical Garden, Phoenix, Arizona 85008 USA; 2 Department of Biology, University of Florida, Gainesville, Florida 32611–8525 USA; 3 Florida Museum of Natural History, University of Florida, Gainesville, Florida 32611–0575 USA; 4 Jardín Botánico Nacional, Universidad de La Habana, La Habana, Cuba

**Keywords:** Caribbean, Cuba, Hispaniola, Jamaica, Leandra, Ossaea, phylogeny

## Abstract

Miconia sect. Lima is an entirely Greater Antillean clade that consists of 19 known species of shrubs and small trees, which were previously recognized under the polyphyletic genera *Leandra* and *Ossaea*. The highest species richness in the clade is represented on Cuba (10 species), followed by Hispaniola (8 species) and then Jamaica (1 species). Here we present a taxonomic revision of the clade based on the study of species in the field, herbarium specimens, as well as a DNA-based phylogeny reconstruction. The *Lima* clade most likely originated on Cuba and then spread to Jamaica once and Hispaniola multiple times. Species of this clade can be recognized by the well developed bulla-based hairs of the adaxial leaf surface, as well as the clavate-dendritic hairs produced along the primary, secondary and tertiary veins of the adaxial leaf surface, mostly towards the leaf base, terminal inflorescences, acute petal apices, slightly bulla-based hairs produced subapically along the petal abaxial surface, and anthers with a dorso-basal appendage and a single, dorsally oriented pore. Descriptions, synonymies, along with distribution maps and illustrations/figures, are given for each species. *Miconia pagnolensis* **sp. nov.** is newly described in this revision.

## Introduction

Tribe Miconieae (Melastomataceae) is a widely distributed, species rich (ca. 1800 species), Neotropical clade of trees and shrubs, which is most diverse in montane regions of Central and South America and the West Indies. The group has been divided into numerous genera, with ca. 16 recognized until only recently (Michelangeli et al. 2008). Phylogenetic analyses have shown that with few exceptions (e.g., *Mecranium* Hook.f.; see Skean 1993), all of these genera are polyphyletic (Bécquer-Granados et al. 2008, Goldenberg et al. 2008, Martin et al. 2008, Michelangeli et al. 2004, 2008; Kriebel et al. 2015, Majure et al. 2015, Reginato and Michelangeli 2016) and undiagnosable, if recognized in the broad sense as currently circumscribed (e.g., *Leandra* Raddi s.l.; Majure et al. 2013). Generic limits in this group have long been considered problematic and arbitrary (Cogniaux 1891, Gleason 1932, Macbride 1941, Wurdack 1972, Judd 1989, Judd and Skean 1991).

**Figure 1.**
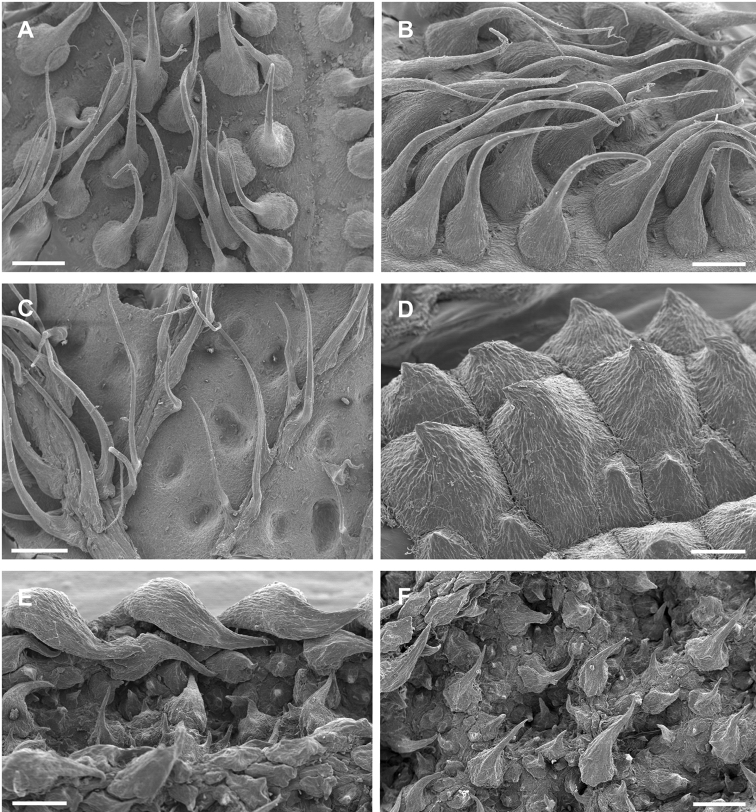
Photomicrographs of indumentum characters and epidermal features of members *Miconia pedunculata* (**A–C**) and *Miconia lima* (**D–F**). **A–B** Adaxial leaf surface of *Miconia pedunculata* (scale bar = 0.75 mm), **B** close-up of bulla-based hairs on adaxial leaf surface (scale bar = 0.6 mm), **C** abaxial leaf surface showing pits and strigose hairs (scale bar = 0.6 mm; all from *Pimentel 806*), **D** adaxial leaf surface of *Miconia lima* showing bulla-based hairs filling areoles (scale bar = 0.375 mm; all from *Judd 5194*), **E** abaxial leaf surface showing marginal, recurved bulla-based hairs covering dentations (scale bar = 0.375 mm), and **F** abaxial leaf surface showing pitting and epidermis covered in bulla-based hairs. All photos taken by G.M. Ionta.

The *Lima* clade (i.e., Miconia sect. Lima Majure & Judd: Miconieae: Melastomataceae) as treated here, is a group of 19 known species that is restricted to the Greater Antilles (excluding Puerto Rico) and occurs mostly in high elevation, montane forests. The island of Cuba contains the largest number of endemic species (10), while 8 species are endemic to Hispaniola and only one species is found on Jamaica. No species are shared between or among islands. Species within this clade are distinctive in their production of bulla-based hairs on most parts of the plant. The adaxial leaf surfaces are often nearly or entirely covered in these hairs (Fig. 1A–B, D), giving the leaves the appearance of lizard or toad skin (Majure and Judd 2013a). The majority of species within this clade previously have been recognized under either *Leandra* or *Ossaea* DC. (Cogniaux 1886, Urban 1921, 1923, 1926–27, 1929, 1931; Judd and Skean 1991, Liogier 2000, Michelangeli and Bécquer 2012), a result of the presence of acute petals produced by almost all members of the group. However, it is clear that *Leandra* and *Ossaea* are polyphyletic (Bécquer-Granados et al. 2008, Goldenberg et al. 2008, Martin et al. 2008, Michelangeli et al. 2004, 2008), and thus the most judicious decision regarding generic circumscription proposed has been to recognize these taxa, as well as most other taxa in the Miconieae (Ionta et al. 2012, Judd and Ionta 2013, Judd and Majure 2013, Majure et al. 2015), under an expanded *Miconia* s.l. (Ionta et al. 2012, Judd and Ionta 2013, Judd and Majure 2013, Majure and Judd 2013a–b, Majure et al. 2013, Majure et al. 2014a–c, Judd et al. 2014a,b, Michelangeli and Goldenberg 2016, Michelangeli et al. 2016), which would include species formerly placed in the genera *Calycogonium* DC., *Charianthus* D. Don, *Clidemia* D. Don, *Conostegia* D. Don, *Killipia* Gleason, *Leandra*, *Maieta* Aubl., *Mecranium*, *Necranium* Britton, *Ossaea*, *Pachyanthus* A. Rich., *Pleiochiton* Naudin ex A. Gray, *Tetrazygia* Rich. ex DC., and *Tococa* Aubl. Miconieae can be recognized by their berry fruit, poorly developed anther appendages, the presence of druse crystals and associated lack of megastyloid crystals in various plant parts (Michelangeli et al. 2008, Ionta et al. 2012), and lack of two pairs of large, decussate bracts subtending each flower, as in tribe Blakeeae (Penneys and Judd 2013).

**Figure 2.**
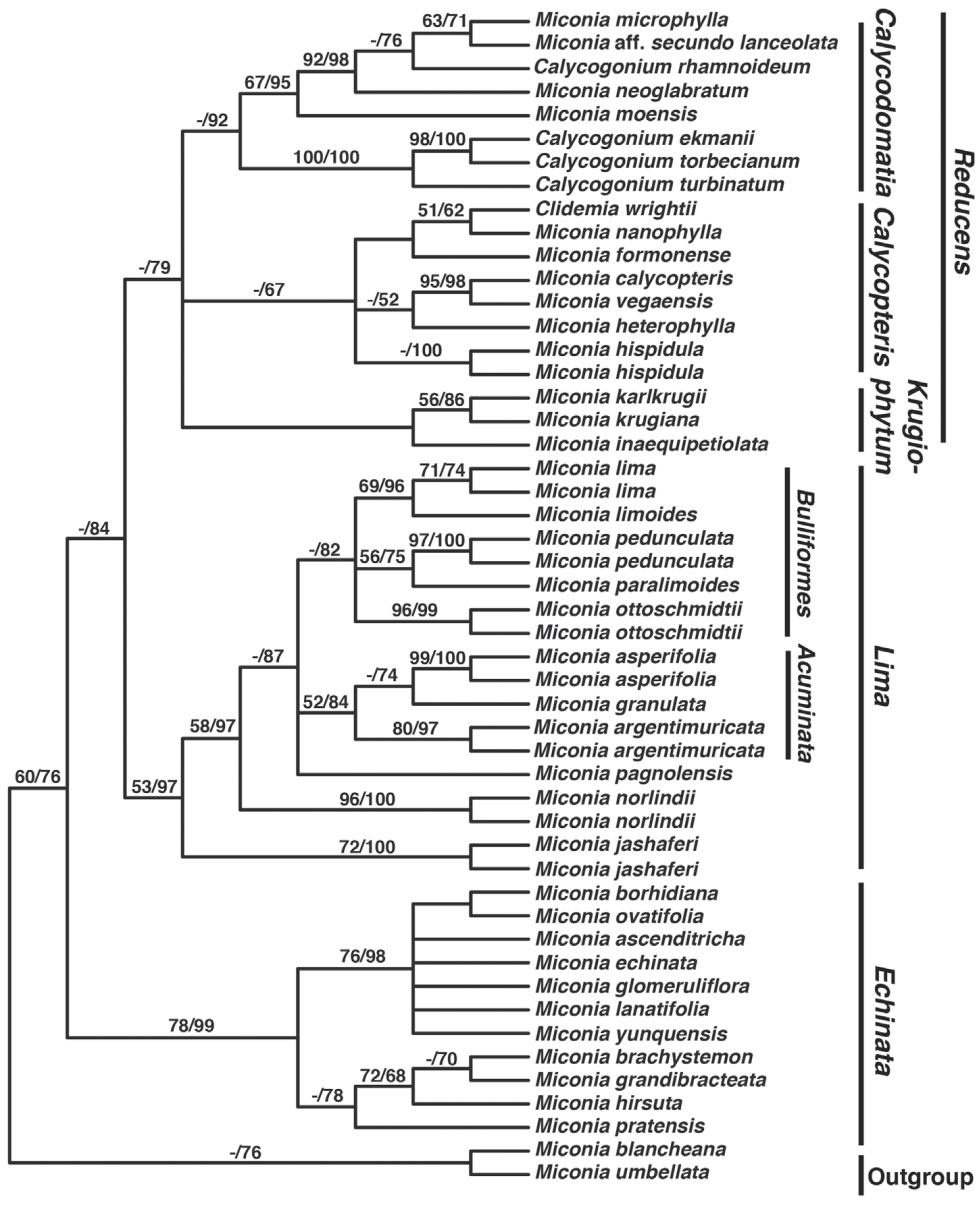
Maximum Parsimony 50% majority rule phylogeny of selected members of the *Lima*, *Calycopteris*, *Calycodomatia*, *Pseudolima*, and *Paralima* clades (modified from Majure et al. 2015). *Miconia umbellata* and *Miconia blancheana* were used as outgroup taxa. Bootstrap values from both maximum parsimony/maximum likelihood analyses are given above branches.

The striking bulla-based hairs produced along the adaxial leaf surface, although best developed in the *Lima* clade, also are found in two other closely related clades, the *Pseudolima* (Miconia sect. Krugiophytum (Cogn.) Majure & Judd; Majure et al. 2014b) and *Paralima* clades (Miconia sect. Echinata Judd, Bécquer & Majure; Majure et al. 2015; Fig. 2). The production of these bulla-based hairs may represent a retained pleisiomorphy in the *Lima*, *Paralima* and *Pseudolima* clades, although character reconstructions using parsimony are equivocal (Majure et al. 2015). Having strongly bulla-based hairs that entirely or nearly completely cover the areoles, however, is a synapomorphy of a subclade (with reversals within it) of the *Lima* clade (Majure et al. 2015). The *Lima* clade can be differentiated from the *Pseudolima* clade by the growth architecture. Members of the *Pseudolima* clade produce terminal inflorescences that often appear axillary as a result of their deflection to a lateral position from the rapid growth of lateral buds. The *Lima* clade has inflorescences that are clearly terminal (Majure et al. 2015). The *Paralima* clade is easily distinguished from the *Lima* clade by the production of globular stellate hairs on the stem, abaxial leaf surface and hypanthium, and several species of the *Paralima* clade produce truly axillary inflorescences or both axillary and terminal inflorescences (e.g., *Miconia glomeruliflora* Judd, Bécquer & Majure, *Miconia ovatifolia* Judd, Bécquer & Majure; Majure et al. 2015, Judd et al. in prep).

## Systematics of Miconia section Lima

### Taxonomic history

The taxonomic history of the *Lima* clade has been one of unstable generic classification beginning with *Miconia lima* (Desr.) M.Gómez. *Miconia lima* was the first species described in this clade and was named by Desrousseaux (1797), as *Melastoma lima* Desr. He did not cite a detailed geographic locality but merely mentioned that the collection was made by *Martin s.n.* in Sainte Domingue (i.e., Haiti). He mentions that the upper leaf surface of the species appeared similar to that of a file, and hence the specific epithet “*lima*” was used. The species was then transferred to *Clidemia* in 1828 (de Candolle 1828), *Sagraea* in 1852 (Naudin 1852), *Calycogonium* in 1866 (Grisebach 1866) and then to *Ossaea* by Triana (1871–72). Gómez (1894) later transferred the species to *Miconia*, and Judd and Skean (1991) made the transfer to *Leandra*. Grisebach (1866) incorrectly cited a specimen from Cuba collected by C. Wright (*189*) as the type; this specimen (i.e., *Wright 189*) was later described as *Ossaea cubana* Alain (Alain 1955). *Miconia asperifolia* (Naudin) Majure & Judd was the second species of the clade to be described (Naudin 1852), as *Clidemia asperifolia* Naudin, apparently from a specimen sent to Naudin from Joseph Hooker (Naudin 1952). Hooker at the time had employed William Purdie to collect plants in Jamaica from 1843–1844 (Sinha 1972), who collected the first known specimen of the species. It was then transferred to *Oxymeris* (Triana 1871–72) and then to *Ossaea* (Triana 1871–72). Cogniaux (1891) described material of the same species as *Leandra eggersiana* Cogn., which was later transferred to *Ossaea* by Urban (1921). It should be noted that MacFayden had already described *Miconia asperifolia*, as *Clidemia hirsuta* Macfad., however, his manuscript (Vol. 2 of his Flora of Jamaica) was apparently not effectively published before his untimely death. It is usually considered to have been unfinished and thus not yet intended for publication; only incomplete copies of the work exist (Stafleu and Cowan 1981, see also Porter-Utley 2015) other than one complete copy at BM. Incomplete copies of the flora were sent out by the British Museum of Natural History to HH, K, MICH, and NY (see Stafleu and Cowan 1981). Two species collected by Charles Wright in Cuba, *Miconia argentimuricata* Majure & Judd and *Miconia cubacinerea* Majure & Judd, were the next species to be described by Grisebach (1866), as *Calycogonium muricatum* Griseb. and *Clidemia cinerea* Griseb., respectively. *Miconia jashaferi* Majure & Judd was then described by Britton and Wilson (1920) as *Ossaea shaferi* Britton & Wilson. Urban (1923, 1926, 1927, 1929), and Urban and Ekman (1929, 1931) then described a series of new species based on material primarily collected by the Swedish botanist Erik L. Ekman (1883–1931) from Cuba and Hispaniola. These were all described under the genus *Ossaea* (i.e., *Ossaea capitata* Urb., *Ossaea granulata* Urb., *Ossaea hybophylla* Urb., *Ossaea limoides* Urb., *Ossaea norlindii* Urb., *Ossaea ottoschmidtii* Urb., *Ossaea polychaeta* Urb., *Ossaea turquinensis* Urb.). Majure and Judd (2013a–b) and Majure et al. (2014a) subsequently described four more species from the clade from Haiti (*Miconia phrynosomaderma* Majure & Judd), the Dominican Republic (*Miconia paralimoides* Majure & Judd) and Cuba (*Miconia bullotricha* Bécquer & Majure, *Miconia hirtistyla* Majure & Judd), and one new species from Massif de la Hotte, Haiti, is described herein. Majure and Judd (2013a) transferred all of the previously described species in this clade to *Miconia* based on our current understanding of the polyphyletic nature of the genera in which they had been placed (Bécquer-Granados et al. 2008, Goldenberg et al. 2008, Martin et al. 2008, Michelangeli et al. 2004, 2008, Majure et al. 2014c).

### Morphology

*Habit and stems*. Species within the *Lima* clade form evergreen shrubs to small trees ranging in height from 0.5 to 5 m tall. The height of several species, however, is completely unknown. Stems are rectangular or cylindrical in cross section and lack longitudinal ridges (Fig. 9M).

*Leaves*. Leaves are opposite, decussate and generally slightly anisophyllous. The blades are elliptical, ovate or lanceolate (narrowly ovate), with crenulate or dentate margins that are generally obscured by bulla-based hairs (Fig. 1E; see Indumentum below); the margins may be flat or slightly revolute. Venation is acrodromous with the 1–3 pairs of secondary veins arching towards the leaf apex; these are basal to suprabasal. Tertiary veins are percurrent, more or less perpendicular to the midvein and often connected by quaternary veins. Leaf shape in certain species is quite useful for species delimitation, such as the elliptical-rhombic leaves of *Miconia marigotiana*, which are unique in the group.

*Indumentum*. Most plants parts within this group are at least to some degree covered in bulla-based hairs. These bulla-based hairs may be lacrimal as in *Miconia pedunculata* (Fig. 1B), volcaniform as in *Miconia argentimuricata*, *Miconia lima*, *Miconia limoides*, *Miconia marigotiana*, *Miconia paralimoides* and several other species (Fig. 1D), or granulate in shape as in *Miconia norlindii* and *Miconia granulata* (Fig. 7B). The degree of bulla-based hair coverage, shape and placement or orientation can be useful for identifying species. Certain species show complete leaf areole coverage, on the adaxial surface, by volcaniform, bulla-based hairs (Fig. 1D), as in *Miconia lima*, *Miconia limoides*, *Miconia marigotiana* and *Miconia paralimoides*. Conversely, *Miconia jashaferi*, *Miconia hirtistyla* and *Miconia cubacinerea* have poorly-formed, slender, lacrimal-shaped bulla-based hairs that cover most, but not all, of the adaxial leaf surface. Clavate-dendritic hairs are common on most species at the leaf blade bases (where the petiole enters the leaf) and are most easily observed on developing leaves. *Miconia jashaferi*, however, exhibits filiform hairs at this position. Clavate-dendritic hairs can commonly be found also at the apices of the calyx tube in most species (but see Morphological Evolution below). Most species within the *Lima* clade exhibit tranluscent or colored glandular hairs, which may be present on most portions of the plant body (stem, both leaf surfaces, hypanthium). These are smaller than and occur between (at the base) or below the bulla-based hairs. Domatia composed of bulla-based or linear hairs are common in certain species, generally at the union of the primary and secondary veins but can also be found at the unions of the tertiary and primary, as well as tertiary and secondary veins (e.g., *Miconia asperifolia*, *Miconia hybophylla*). The directionality of the bulla-based hairs of the stem of specific species is very useful for species delimitation. For example, only *Miconia limoides* and *Miconia phrynosomaderma* exhibit downward oriented hairs on the stem surface.

**Figure 3.**
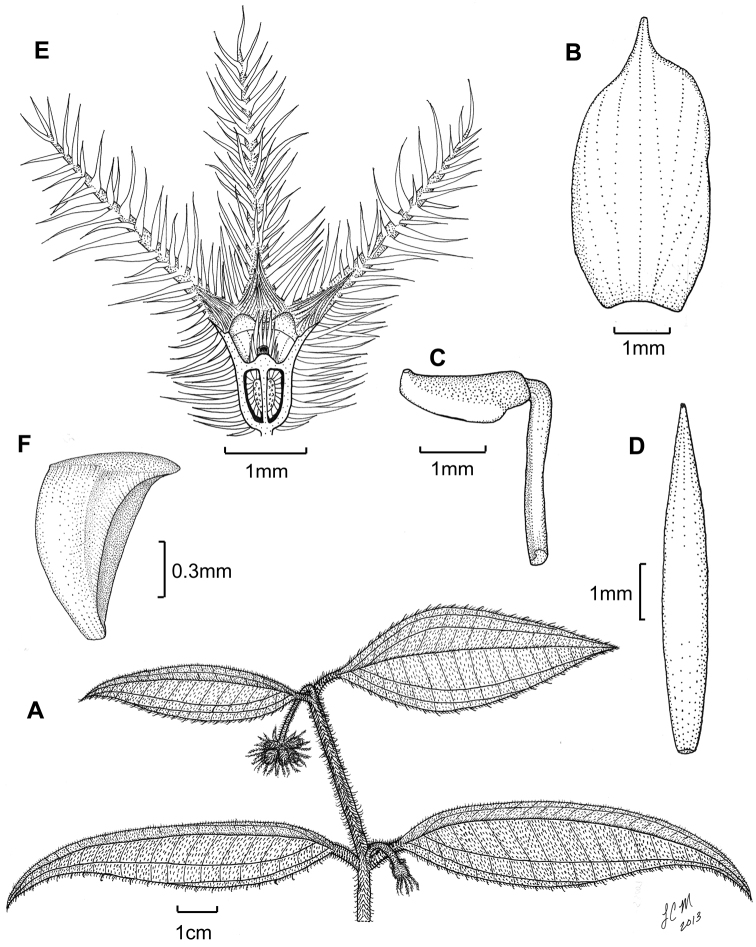
Illustration of *Miconia jashaferi* **A** habit (*Ekman 3849*) **B** petal (*Alain 871*) **C** stamen (*Alain 871*) **D** style (*Alain 871*) **E** immature fruit longitudinal section (*Alain 871*) **F** seed (*Acuña SV-13275*). Reproduced with permission from Majure et al. (2014a).

*Inflorescences*. Inflorescences are terminal in all species of the *Lima* clade and are formed typically from 3-flowered dichasia; inflorescences are generally quickly surpassed by rapidly growing, lateral shoots. The flowers may be pedicellate or sessile and thus form open to relatively dense, and even slighly capitate (e.g., *Miconia jashaferi*; Fig. 3A) cymose inflorescences. Bracts subtending 3-flowered dichasia are either granulate, lacrimal or in some taxa (e.g., *Miconia pedunculata*; Fig. 22A) foliose. Bracteoles are similar in form to the bracts.

*Hypanthium and calyx lobes*. The hypanthia are mostly 4–5 lobed, or mostly speherical (Fig. 12H). However, slight lobing is often obscured by the production of dense bulla-based hairs. Calyx lobes are triangular with acute to acuminate apices (Fig. 3E). The calyx teeth may be equal or longer than the calyx lobes and are terete (Fig. 3E) and often reflexing when in fruit.

*Corollas*. Corollas are actinomorphic or nearly so, with 4–5 ovate to obovate or oblong petals; these are symmetric or slightly assymetric with acute to acuminate apices. The petal apices often exhibit 1–several bulla-based hairs on the abaxial surface (e.g., Fig. 14B). Petals are white, rose, red, purple or white with a purple tinge abaxially and are generally reflexed in flower.

*Stamens*. Stamens are 8–10 with glabrous filaments. The anthers are white to yellow and may or may not have a dorso-basal appendage; they typically have one, dorsally-inclined pore (Fig. 3C).

*Gynoecium*. The gyneocium in the *Lima* clade ranges from 2–7 carpellate, with *Miconia marigotiana* being the only species with two carpels (Fig. 25G). The ovary is inferior with axile placentation, with the placentas generally deeply intruded into the ovary locule. The upper portion of the ovary may be glabrous or pubescent. The style is straight or slightly curved with a punctiform stigma. Only *Miconia hirtistyla* exhibits a pubescent style (Fig. 5F), while the rest of the species have glabrous styles. However, a crown of needle-like hairs often is present at the base of the styles (along ovary apex) of most species (e.g., Fig. 3E).

*Fruit*. Fruit in the *Lima* clade are multi-seeded, mostly spherical berries that range in color from purple, purple-black to black (Figs 9I, N, 15F, 19C, 27D). There is still much missing data for fruit color and size for numerous taxa within this group, as fruit are not well-preserved in herbarium specimens, and many specimens lack fruit altogether.

### Phylogenetics and evolution

Majure et al. (2015) reconstructed a phylogeny of the *Lima* clade using five plastid intergenic spacers (*accD-psaI*, *psbK-psbI*, *rpl32-trnL*, *trnH-psbA*, *trnV-ndhC*) and two nuclear, ribosomal loci (ETS and ITS). They included 11 of the 19 species of the *Lima* clade, i.e., all species for which material was available in silica from field collections or from “recently” collected herbarium material (e.g., within the last 10 years). They were able to include two accessions per species for six of the included species (Fig. 2). Other closely related members of the Caribbean clade were included as outgroup taxa (i.e., Miconia sect. Calycopteris Judd, Bécquer & Majure; Judd et al. 2014; Miconia sect. Echinata Judd, Bécquer & Majure; Majure et al. 2015; Miconia sect. Calycodomatia Skean, Judd & Majure; Majure et al. 2015, Miconia sect. Krugiophytum (Cogn.) Majure & Judd; Majure et al. 2014b, as well as *Miconia blancheana* Urb. and *Miconia umbellata* (Mill.) Judd & Ionta). The phylogeny presented here is based on Majure et al. (2015). The *Lima* + *Calycopteris* + *Paralima* + *Pseudolima* + *Calycodomatia* clade is referred to as the Sandpaper clade, because of the many species of the group that have bulla-based hairs on the upper leaf surface (see Majure et al. 2015; Fig. 2).

The *Lima* clade is most closely related to the *Reducens* clade, composed of Miconia sect. Calycopteris, sect. Krugiophytum and sect. Calycodomatia. Most of the members of those other three sections have reduced inflorescences and lack the conspicuous bulla-based hairs of the *Lima* clade (except for sect. Krugiophytum). They also have conspicuous to reduced globular-stellate hairs, again, except for sect. Krugiophytum, whose members possess sessile-glandular hairs like those of the *Lima* clade (Majure et al. 2015).

Within the *Lima* clade, *Miconia jashaferi* is resolved as sister to the remaining species, although other putative close relatives of *Miconia jashaferi* have not been sampled (e.g., *Miconia cubacinerea*, *Miconia hirtistyla*, *Miconia tentaculicapitata*). It is most likely that these four species form a subclade sister to the rest of the *Lima* clade, as they have the putative synapomorphies of leaf-like bracts and bracteoles, long, filiform hairs on the adaxial calyx lobe and calyx tube apex (homologs of clavate-dendritic hairs on other species of the clade; Majure et al. 2015), the lack of dorso-basal anther appendages and 3-locular ovaries. In our phylogeny, the first two diverging members of the *Lima* clade, *Miconia jashaferi* and *Miconia norlindii*, are Cuban species, as are other putative relatives of *Miconia jashaferi* not included in our analyses (i.e., *Miconia cubacinerea*, *Miconia hirtistyla*, *Miconia tentaculicapitata*). The *Miconia lima* complex of Hispaniola (sensu Majure and Judd 2013a,b), composed of *Miconia lima*, *Miconia limoides*, and *Miconia paralimoides* in our phylogeny, are phylogenetically adjacent, however, *Miconia paralimoides* apparently is more closely related to *Miconia pedunculata* than to either *Miconia lima* or *Miconia limoides*, contrary to relationships initially suspected based on morphology alone (Majure and Judd 2013b). *Miconia limoides* is sister to *Miconia lima*, and the Cuban *Miconia ottoschmidtii* is unresolved in the same subclade as well (i.e., the *Bulliformes* subclade; Majure et al. 2015). Thus, *Miconia ottoschmidtii* and *Miconia pedunculata* must be included in what Majure and Judd (2013a) termed the *Miconia lima* complex, which we refer to here as the *Bulliformes* subclade. The *Bulliformes* subclade is restricted to Hispaniola and Cuba. *Miconia marigotiana* and *Miconia phrynosomaderma* also are likely members of the *Bulliformes* subclade, although they have not been recollected since the type material was collected by Ekman. They are linked to the *Bulliformes* subclade by their bulla-based hairs on the adaxial leaf surface almost completely filling the leaf areoles, as well as the descending stem hairs in *Miconia phrynosomaderma* (found otherwise only in *Miconia limoides* of the *Bulliformes* subclade). Another subclade is formed by *Miconia argentimuricata*, *Miconia asperifolia*, *Miconia granulata* and *Miconia pagnolensis* (i.e., the *Acuminata* subclade) represented by members from Cuba, Haiti, and Jamaica. *Miconia cubana* and *Miconia hybophylla* also are most likely members of this subclade, as they produce large, open inflorescences (i.e., in *Miconia cubana* and *Miconia hybophylla*, as in *Miconia asperifolia* and *Miconia argentimuricata*). They also tend to have less well-developed bulla-based hairs on the upper leaf surface, similar to other members of the *Acuminata* clade. *Miconia hybophylla* also is linked to *Miconia asperifolia* by the production of well-developed domatia in the axils of the primary and secondary, as well as the primary and tertiary and secondary and tertiary veins. *Miconia bullotricha* may also form part of the *Acuminata* subclade in that it produces deflexed inflorescences, which are similar to the partially deflexed inflorescences in *Miconia granulata*, demonstrates a similar leaf shape (narrowly ovate), as do both *Miconia granulata* and *Miconia argentimuricata* in many specimens, and occurs over serpentine soils, as do both *Miconia argentimuricata* and *Miconia granulata*.

*Miconia argentimuricata*, *Miconia asperifolia*, *Miconia jashaferi*, *Miconia lima*, *Miconia norlindii*, *Miconia ottoschmidtii*, and *Miconia pedunculata* are likely cladospecies (sensu Donoghue 1985), as multiple accessions of each of these species form clades in our phylogeny (Majure et al. 2015; Fig. 2). We were unable to assess the monophyly of the rest of the species, as multiple accessions were unavailable for phylogenetic analysis. However, considering the morphological apomorphies uniting the populations of most of the other members of the clade, they also likely represent cladospecies (see Majure and Judd 2013a-b, Majure et al. 2014a).

### Biogeography

Ancestral area reconstruction, as carried out in Majure et al. (2015), suggests that the *Lima* clade evolved in the northeastern mountains of Cuba (Sierra de Baracoa, Moa, and Cristal) and then subsequently spread to other mountain ranges in Cuba (Sierra Maestra, Trinidad Mts. and Pinar del Río), as well as to Hispaniola (likely four times) and Jamaica (once). There were three separate movements into the Sierra Maestra of Cuba represented by *Miconia argentimuricata*, *Miconia norlindii* and *Miconia ottoschmidtii*. The *Acuminata* subclade originated in eastern Cuba, and subsequently dispersed to the Massif de la Hotte, Haiti (*Miconia pagnolensis*) and to Jamaica (*Miconia asperifolia*). The unsampled species, *Miconia hybophylla*, most likely sister to *Miconia asperifolia*, would have represented yet another dispersal of the *Lima* clade to Hispaniola; thus four dispersals to Hispaniola from Cuba are most likely for the *Lima* clade. The *Bulliformes* subclade dispersed twice to Hispaniola, once to the Massif de la Selle-Sierra de Baoruco (*Miconia lima*, *Miconia limoides*) and once to the Cordillera Central (*Miconia paralimoides*, *Miconia pedunculata*). There was then broader dispersion of *Miconia lima* throughout Hispaniola and a subsequent dispersal of *Miconia limoides* to the Massif de la Hotte (Majure et al. 2015).

The *Lima* clade appears to have evolved primarily on serpentine soils of Cuba and then dispersed and adapted several times to limestone-derived soils, soils mixed with limestone, or other substrates (e.g., clay soils in *Miconia asperifolia*; Majure et al. 2015). A number of taxa within the *Lima* clade are still mostly restricted to serpentine soils (*Miconia bullotricha*, *Miconia granulata*, *Miconia jashaferi*), or at least occur in parts of their range on serpentine (*Miconia argentimuricata*, *Miconia ottoschmidtii*).

### Morphological evolution

There are very few morphological synapomorphies for the *Lima* clade, but the production of clavate-dendritic hairs on the upper leaf surface is a synapomorphy of a subclade of the group (only excluding *Miconia jashaferi* in our analyses), as is the production of dilated styles (excluding only *Miconia jashaferi* and *Miconia norlindii*, the two basalmost taxa in our phylogeny). The production of very well-developed bulla-based hairs on the upper leaf surface that mostly fill the areoles also is a synapomorphy of a subclade (excluding *Miconia jashaferi* and *Miconia norlindii*), and the production of sessile, glandular hairs is a synapomorphy for the entire clade. The sessile-glandular hairs on the upper and lower leaf surfaces are homologs to the globular-stellate hairs produced in close relatives (*Calycopteris* and *Calycodomatia* clades; Majure et al. 2015) and have also evolved in the *Pseudolima* clade, as well as a few members of the *Paralima* clade. Thus, although the sessile-glandular hair type is synapomorphic for the *Lima* clade, it is not a unique synapomorphy within the Sandpaper clade (Majure et al. 2015).

Suites of characters that can be used to diagnose members of the *Lima* clade include multicellular, bulla-based hairs occuring over most surfaces of the plant, including the ad- and abaxial leaf surface, petioles, stem, inflorescence axes and hypanthia; sessile-pigmented glands on the lower leaf surface, as well as on the hypanthium; deciduous, dendritic hairs on adaxial leaf surface arising from the lamina between the bulla-based hairs, these long stipitate and flattened or clavate at the apex; ovate or oblong-triangular petals with slightly bulla-based, subapical hairs, these usually extending past the acute apex of the petal. Other characters unifying most species in this clade are anthers with a dorso-basal appendage and dorsally-oriented single pore, hypanthia that are constricted below the torus, and which are often slightly 4-lobed, styles that are slightly expanded (dilated) in the middle, and obpyramidal seeds with a smooth testa and dark raphe extending the length of the seed.

### Ploidy

Chromosome numbers are unknown in this group.

### Species concepts

Criteria for the recognition of species in this group are based upon morphological cohesiveness or the morphological-phenetic/diagnostic species concepts (Judd 1981, Wheeler and Platnick 2000, Judd 2007), as well as phylogenetic or apomorphic species concepts (Donoghue 1985, Judd 2007), thus unifying our knowledge regarding the evolutionary history of a species along with its morphological characteristics (i.e., producing a more unified species concept; DeQueiroz 2007). A species thus consists of populations of individuals that can be morphologically diagnosed from other populations based on synapomorphies or suites of characters, and when the information is available, also form part of the same lineage or clade. Species consisting of metaspecies complexes and resulting from the invasion of new niches by closely related and subsequently divergent populations (peri- or parapatric speciation, or peripherial isolate speciation) would appear to be a highly likely in this clade, considering the restriction of many of these taxa to high elevation mountain ranges (see Judd et al. 2015a for an example of this). A given population evolving within a metapopulation could easily diverge from the rest of the lineage via niche specialization or other processes leading to allopatric speciation. However, within the *Lima* clade, we do not have direct evidence of speciation via metaspecies lineages; all of the species included in phylogenetic analyses with multiple accessions appear to form clades. So it appears that the most common mode of speciation in this group is through cladogenesis, which may be brought about through the restriction of many species to specific ecological niches (e.g., high elevation cloud forests on limestone-derived, clay soils, or other substrates or low elevation sclerophyllous forests on serpentine soils). Thus, most species within the *Lima* clade have at least a few apomorphic characters (uniting their populations), as well as quantitative character differences, which may or may not be apomorphies. We do not have any information regarding ploidy in this group, so we cannot determine whether or not hybridization and subsequent genome duplication may have played a role in the origin and subsequent evolution of any of the species.

## Materials and methods

A total of 894 herbarium specimens were observed and measured to determine morphological variability among and within species of the *Lima* clade. Herbaria from which specimens were studied are A, ALBC, BR, F, FTG, G, GH, HAJB, IJ, JBSD, JE, K, MICH, MO, NY, S, UCMM, and US. Abbreviations follow Thiers (2012). Acronyms and citation of collection numbers for Cuban specimens follows Regalado Gabancho et al. (2008). Altogether, more than 100 quantitative and qualitative morphological characters were analyzed from herbarium specimens, photos, alcohol preserved material, or from live plants in the field. Phenological, distributional and ecological data were derived mostly from specimen label data or observations taken in the field. Measurements for reproductive characters (e.g., hypanthium length, calyx tube, lobe and tooth length, ovary length and width, etc.) were based on Judd (2007) and were taken from herbarium material rehydrated via boiling for several minutes in water mixed with a detergent (see Judd 2007). Illustrations were based on herbarium specimens.

Typifications of species described by Urban are carried out, in part, using the criteria of Bécquer (2012). However, as Urban never actually explicitly stated the repository of type material, and there is potential confusion as to where holotypes may have originally been deposited, since all specimens of Melastomataceae were lost in the allied bombings of Berlin during the second World War, we have chosen to lectotypify those species for clarity (see also McNeill 2014).

Although Alain and Liogier both refer to Henri Alain Liogier, we maintain those entries seperately, as Liogier used only the name, Bro. Alain, while collecting in Cuba, but generally used Henri Alain Liogier (or Alain Henri Liogier) while collecting in Hispaniola after he left the Catholic religious order. Collections made by Erik Ekman in Hispaniola are preceeded with an H, as his collection numbers were started anew during his work in Haiti and the Dominican Republic (see also Index to numbered collections).

## Results

### Miconia sect. Lima

Taxon classificationPlantaeMyrtalesMelastomataceae

Majure & Judd, J. Bot. Res. Inst. Texas. 7: 266. 2013

#### Type.

*Miconia lima* (Desr.) M.Gómez (*Melastoma lima* Desr., Encycl. [J. Lamarck & al.] 4: 47. 1797).

#### Description.

Evergreen shrubs to small trees; young stems terete, elliptic or slightly rectangular in cross section, lacking longitudinal ridges, the indumentum of dense bulla-based hairs, these long appressed, spreading, or recurved, or short and granulate. Leaves opposite, slightly anisophyllous; blade elliptical, ovate, or lanceolate, the margin crenulate to dentate, these crenulations/dentations obscured by large bulla-based hairs, which slightly fold over the leaf margin, producing in some cases a moderately revolute margin, the indumentum of adaxial leaf surface typically of broad bulla-based hairs and more or less filling the areoles, although sometimes these hairs relatively narrow and wide-spaced, not filling the areoles, with long-stemmed, clavate-dentritic hairs produced along the primary, secondary and tertiary veins from between the bulla-based hairs, and also sessile to short-stalked glandular hairs present on all parts of the lamina (between bulla-based hairs), the abaxial leaf surface variously covered by narrow bulla-based hairs, these either long and well developed or short and granulate, these appressed, spreading, or erect, the lamina with sparse, sessile glands, the venation acrodromous, with secondary veins arching toward leaf apex, 1 to 3 pairs, basal to suprabasal, tertiary veins percurrent, more or less perpendicular to the midvein, sometimes mostly obscured by bulla-based hairs on the adaxial leaf surface, connected by quaternary veins, the primary, secondary, tertiary and quaternary veins mostly impressed on the adaxial surface and raised on the abaxial surface, domatia present or absent, occurring at the junctions of primary, secondary and tertiary veins, forming a pocket-like structure in the axils of the primary and innermost secondary veins or formed from a tuft of hairs in the vein axils. Inflorescences terminal, although often surpassed by the rapid growth of axillary shoots, the flowers in 3-flowered dichasia, sessile, subsessile or pedicellate, thus forming open cymes or sessile and nearly headlike clusters. Flowers 4–5(7)-merous, mostly actinomorphic or nearly so; hypanthium 4–5-lobed, the lobes sometimes obscured by retrorse or antrorse bulla-based hairs, bulla-based hairs long and well developed, or granulate, hypanthium also with sessile glands; calyx lobes triangular, acute to acuminate, often covered by sessile glands throughout the adaxial surface or such glands restricted to the apex of the adaxial surface, abaxial surface covered in bulla-based hairs and more or less sessile glands; calyx teeth ca. equal to or longer than calyx lobes, terete, mostly reflexed in fruit, covered in long and well developed, or granulate bulla-based hairs, sessile glands present or absent; calyx tube often with long stemmed, clavate-dendritic hairs produced from apex along the margin, sessile glands more or less present on adaxial surface, abaxial surface covered in bulla-based hairs; petals ovate to obovate or slightly oblong, symmetric or asymmetric, white, red, rose, purple, or white with purple tinge abaxially, apices acute to acuminate, with moderately bulla-based hairs produced from the abaxial surfaces just below the petal apex and occasionally from the medial portion of the petal as well; stamens 8–10, not geniculate, the filaments glabrous, the anthers with or without a small dorso-basal appendage and a single, dorsally inclined pore; style straight to moderately curved, generally expanded in the middle, the stigma punctiform; ovary 2–5(7) locular, more or less inferior, with axile placentation, the placenta intruded into each locule, the ovary apex without a collar but commonly with a crown of multicellular hairs, the upper portion of the ovary pubescent (bulla-based hairs) to mostly glabrous (i.e., with only crown hairs present). Fruit a globose and slightly 4- or 5-lobed berry, purple-black at maturity. Seeds angular, obpyramidal, obovoid to obovoid-falcate, with a linear to oblong, dark colored raphe that extends the length of the seed; testa smooth; appendage absent.

Miconia sect. Lima is a clade of 19 species restricted to the Greater Antilles (excluding Puerto Rico).

### Key to the species of the *Lima* clade

**Table:** 

1	Upper leaf surfaces appearing velvety or soft, covered in narrowly dilated (poorly developed bulla-based) hairs with long, attenuate apices, these mostly not covering leaf areoles, thus the lamina clearly visible	**2**
–	Upper leaf surfaces appearing as a rasp or file, or lizard or toad skin, covered in broadly dilated (well developed bulla-based hairs), with short attenuate to truncate apices, these mostly covering the leaf areoles, thus the lamina mostly obscured, or if with long attenuate apices, then bulla-based hairs well developed	**5**
2	Inflorescence an expanded, compound cyme, very delicate, with proximal inflorescence branches 8–25 mm long; bracts and bracteoles not foliose; calyx lobes and teeth 4; ovary 4-locular and 4-lobed; Pinar del Río, Cuba	**9. *Miconia cubana***
–	Inflorescence a dense cluster of sessile flowers (glomerulate), robust, with proximal inflorescence branches 0–5.5 mm long; bracts and bracteoles foliose; calyx lobes and teeth 5–7; ovary 3-locular and unlobed; eastern Cuba	3
3	Leaf apices narrowly acute to acuminate; leaf margins composed of large and small bulla-based hairs (appearing jagged); inflorescences pendant; Baracoa, Moa, Sierra de Cristal, eastern Sierra Maestra, Cuba	**1. *Miconia jashaferi***
–	Leaf apices broadly acute; leaf margins with only one size of bulla-based hairs (appearing smooth); inflorescences erect; Baracoa and western Sierra Maestra, Cuba	**4**
4	Abaxial leaf surface conspicuously pitted as a result of the bulla-based hairs produced on the adaxial surface; styles pubescent; calyx teeth 4.5–4.6 × 0.2–0.4 mm; Pico Turquino region, Cuba	**2. *Miconia hirtistyla***
–	Abaxial leaf surface not conspicuously pitted; styles glabrous; calyx teeth 5.7–6.2 × 0.6–0.7 mm; Baracoa region, Cuba	**3. *Miconia cubacinerea***
5	Inflorescence bracts and bracteoles ovate, obovate or elliptic, broad and foliaceous; bracteoles 3–7 × 1.6–6.5 mm; flowers in glomerulate heads; inflorescences appearing long pedunculate or sessile	**6**
–	Inflorescence bracts and bracteoles oblong, acute or linear, not broad and foliaceous; bracteoles 0.4–3.5 × 0.15–0.8 mm; flowers well separated or if in glomerate heads then in expanded inflorescences not appearing long pedunculate or sessile	**7**
6	Leaves 5-veined; adaxial leaf surface with bulla-based hairs completely covering leaf areoles, not produced in rows; proximal inflorescence branches absent; Pico Turquino region, Cuba	**4. *Miconia tentaculicapitata***
–	Leaves 7–9-veined; adaxial leaf surface with bulla-based hairs not completely covering leaf areoles, well spaced and produced in lateral rows; proximal inflorescence branches 15–45 mm long; Cordillera Central, Dominican Republic	**18. *Miconia pedunculata***
7	Stem indumentum of descending bulla-based hairs with the apices recurved upwards, the hairs long and shaggy	**8**
–	Stem indumentum of ascending or spreading bulla-based hairs, the hairs long and shaggy or granulate	**9**
8	Areoles of adaxial leaf surface completely filled by bulla-based hairs; abaxial leaf surface completely covered in erect to slightly spreading bulla-based hairs; flowers sessile or subsessile; petals not clawed; anthers lacking dorso-basal appendage (or very poorly developed to 0.1mm long); calyx teeth erect or spreading, not reflexed in fruit; restricted to southern Hispaniola, i.e., Massif de la Selle/Sierra de Bahoruco	**14. *Miconia limoides***
–	Areoles of adaxial leaf surface not completely filled by bulla-based hairs; abaxial leaf surface visible, bulla-based hairs mostly restricted to veins, appressed or slightly spreading; flowers pedicellate; petals clawed; anthers with dorso-basal appendage to 0.3 mm long; calyx teeth reflexed in fruit; restricted to Massif du Nord, Haiti	**15. *Miconia phrynosomaderma***
9	Stem indumentum mostly granulate, the hairs mostly spreading or appearing so, their apices truncate or short attenuate, with longest hairs to 0.2–0.5 mm long	**10**
–	Stem indumentum of longer hairs, their apices long attenuate, mostly ascending or occasionally spreading, with longest hairs 0.6–2.2 mm long	**15**
10	Bulla-based hairs on adaxial leaf surface rounded with conspicuously large and small hairs, not meeting at the bases, thus areoles not completely filled	**12**
–	Bulla-based hairs on adaxial leaf surface angled, the bases expanded, appearing to meet at the bases, thus completely filling the areoles	**14**
12	Petiole with two hairs types, adaxial surface with long attenuate hairs, abaxial surface with granulate hairs; domatia very well developed in the axils of the primary and secondary, primary and tertiary veins, and secondary and tertiary veins; Haiti	**7. *Miconia hybophylla***
–	Petiole with one hair type, granulate on both surfaces or only slightly elongated; domatia poorly developed, if present restricted to the axils of the primary and secondary veins, Cuba	**13**
13	Leaf apices narrowly acute to acuminate; domatia absent; leaves 3-veined, narrowly ovate to narrowly elliptic	**8. *Miconia granulata***
–	Leaf apices widely acute to obtuse; domatia present at least at the junction of primary and secondary veins; leaves 5-veined, broadly elliptic to broadly ovate	**5. *Miconia norlindii***
14	Stem indumentum generally with apices attenuate and strongly recurved upwards; abaxial leaf surface hairs erect throughout the lamina and along veins; innermost pair of secondary veins produced 2–6 mm from the leaf base; floral buds globose; calyx teeth 1.75–2.2 mm long; mountains of Guantánamo province	**11. *Miconia bullotricha***
–	Stem indumentum generally granulate with apices truncate or only short attenuate and recurved upwards or not; abaxial leaf surface hairs mostly spreading or appressed throughout the lamina, appressed to spreading along the veins; innermost pair of secondary veins produced 0.8–25 mm from the leaf base; floral buds quadrangular; calyx teeth 0.4–0.8 mm long; widespread on Cuba	**12. *Miconia ottoschmidtii***
15	Abaxial leaf surface covered in dense, strongly appressed hairs mostly obscuring the epidermis; inflorescence 0.9–2.8 × 1.2–2.8 cm, stout; restricted to the Cordillera Central, Dominican Republic	**17. *Miconia paralimoides***
–	Abaxial leaf surface covered in sparse, erect, spreading or moderately appressed hairs, not obscuring the epidermis; inflorescence 1.9–7.6 × 1–9.8 cm, delicate; Cuba, Hispaniola, Jamaica	**16**
16	Abaxial leaf surfaces with bulla-based hairs mostly restricted to veins, thus the lamina clearly visible; petals with 2–4 subapical hairs; ovaries 5 locular; leaves ovate to broadly elliptic; eastern Cuba and Jamaica	**17**
–	Abaxial leaf surfaces with bulla-based hairs not restricted to veins, thus the lamina partially or almost entirely obscured; petals with 1–2 subapical hairs; ovaries 2–4 (5) locular; leaves mostly elliptic, narrowly elliptic, elliptic-rhomboid, or occasionally ovate; Cuba and Hispaniola	**18**
17	Leaves drying silver to bronze; adaxial leaf surface covered in basally strongly angular, well-formed bulla-based hairs of mostly one size, these usually with long attenuate apices; tertiary veins inconspicuous on adaxial surface; hypanthium hairs to 2.7 mm long, thus concealing the hypanthium; ovary not or only weakly lobed; eastern Cuba	**10. *Miconia argentimuricata***
–	Leaves drying green, yellow or brown, adaxial leaf surface covered in basally rounded bulla-based hairs of conspicuously small and large sizes, the large hairs usually surrounded by the smaller hairs, apices either truncate or short attenuate; tertiary veins conspicuous on adaxial surface; hypanthium hairs to 0.4 mm long, not concealing the hypanthium; ovary strongly 5-lobed; Jamaica	**6. *Miconia asperifolia***
18	Leaves narrowly ovate to elliptic-rhomboid, apices narrowly acute; abaxial leaf surface hairs appressed; carpels 2	**16. *Miconia marigotiana***
–	Leaves elliptic, narrowly elliptic or ovate, apices acute or truncate; abaxial leaf surface hairs appressed, spreading or erect; carpels 4–5	**19**
19	Shrubs 1.5–5 m tall; leaf blades 0.7–5.7 × 0.42–3.2 cm, apices acute to acuminate; petioles 3–21 mm long; widespread on Hispaniola	**13. *Miconia lima***
–	Shrubs 0.5–0.9 m tall; leaf blades 0.9–2.3 × 0.5–1.2 cm, apices obtuse to acute; petioles 1.5–4.5 mm long; restricted to Massif de la Hotte, Haiti	**19. *Miconia pagnolensis***

### Taxonomic treatment

#### 1. Miconia jashaferi

Taxon classificationPlantaeMyrtalesMelastomataceae

Majure & Judd, J. Bot. Res. Inst. Texas. 7: 268. 2013.

Figs 3 , 9A–D

Ossaea shaferi Britton & Wilson, Mem. Torrey Club 16: 92. 1920. Type: CUBA. Camp La Gloria, South of Sierra Moa, 24–30 Dec 1910, *J.A. Shafer 8152* (holotype: NY! [NY00099714]; isotypes: A! [A00073129], CAS [CAS0003716]).

##### Type.

Based on *Ossaea shaferi* Britton & Wilson

##### Description.

Evergreen shrub, 1–1.5 m tall; stems round in cross section, not ridged, the internodes 0.4–9.5 cm long, stem indumentum of spreading to descending bulla-based hairs, the apices recurved upwards, hairs to 2.3 mm long; nodal line absent. Leaves opposite, decussate, ovate or elliptic, 2.9–12.8 × 1.8–4.7 cm, slightly anisophyllous, apex acute to acuminate, leaf base acute to rounded, venation acrodromous, 5-veined, the midvein and 2 pairs of arching secondary veins, the outermost usually intramarginal, secondary veins mostly basal, the innermost pair suprabasal, produced 2–13 mm from leaf base, positioned 3–7 mm in from margin at widest point of blade, tertiary veins percurrent, more or less perpendicular to midvein, 2–6 mm apart at midleaf, impressed, but clearly visible on the adaxial leaf surface, intertertiary veins present, usually prominent, tertiary veins often joined by quaternary veins; adaxial leaf surface densely covered in bulla-based hairs giving the upper leaf a velvety appearance, these mostly filling the leaf areoles, widest hair bases to 1.1 mm, apices of bulla-based hairs mostly recurved, young leaf adaxial surface not producing long-stemmed, clavate-dentritic hairs, sessile, glandular hairs produced along the primary, secondary, tertiary, and quaternary veins between the bulla-based hairs; abaxial leaf surface covered in bulla-based hairs, these erect, those along the primary, secondary, and tertiary veins larger than hairs on the clearly visible lamina, lamina with a series of pits from depressions of the bulla-based hairs produced from the upper leaf surface, sessile to short stipitate, glandular hairs produced primarily along major and minor veins but also occasionally throughout the lamina; petioles 0.4–1.3 cm long, covered in bulla-based hairs on both surfaces, these spreading with apices recurved upwards. Inflorescences terminal, pendant, forming mostly 5–6 flowered glomerules, only occasionally branching, 1.2–3.9 × 1.4–2.7 cm, the peduncle 0.2–2.3 cm long, proximal inflorescence branches absent to 5.5 mm long, pedicels absent; bracts foliaceous, oblong to ovate, 5.7–11 mm long; bracteoles flat, foliaceous, ovate to broadly ovate or rotund, 2.9–3.7 × 1–1.7 mm, the margins and apex with long bulla-based hairs and filiform gland-headed hairs, with sessile, glandular hairs along the adaxial surface and bulla-based hairs covering the abaxial surface, the adaxial surface black in herbarium specimens. Flowers 4–6-merous, sessile; hypanthium 3.4–6.5 mm long, short-oblong to globose, unlobed, slightly constricted below the torus; free portion of the hypanthium 1–1.3 mm long, abaxial surface covered in bulla-based hairs to 2.5 mm long, and occasional, sessile, glandular hairs near the bases of the bulla-based hairs, adaxial surface (i.e., free portion) with few, short, linear hairs and sessile, glandular hairs; calyx teeth 4–6, 6.8–7.5 × 0.2–0.4 mm, ascending or spreading, covered in bulla-based hairs; calyx lobes more or less triangular, apex acute to rounded, 0.9–1.7 × 1–1.2 mm, covered in bulla-based hairs abaxially and sessile, sparse, glandular hairs, as well as filiform hairs adaxially, these often expanded and flattened at the apex or gland-headed; calyx tube not tearing, 0.2–0.4 mm long with bulla-based hairs abaxially and sessile, glandular hairs adaxially, filiform hairs produced from the apex of the tube; petals 4–6, white, broadly elliptic to obovate, 5.3–5.6 × 2.1–2.7 mm, with an acute or acuminate apex, without or with one slightly bulla-based hair produced abaxially just below the apex to 0.1 mm long; stamens 8–12, filaments 2.2–2.6 mm long, glabrous, anthers 1.8–2 mm long, with one dorsally oriented pore, anther thecae 1.5–1.7 mm long, without a dorso-basal appendage; style 5.4–6 mm long, glabrous, not or only slightly dilated in the middle, collar absent, style subtended by a crown of multicellular, linear to elongate-triangular (needle-like) hairs, which are notably longer than the surrounding bulla-based hairs of the ovary apex, stigma punctate; ovary 2–5.3 × 1.9–4.3 mm, apex flat (not upraised), with bulla-based hairs, except for the linear or elongate-triangular hairs forming crown, placentation axile with deeply intruded placenta, 3-locular; berries globose, slightly 4-lobed, purple at maturity, 7.5 mm long (including calyx tube), 7.3 mm wide; seeds 0.8–1.6 mm long, obpyramidal, often falcate, testa smooth, light brown, raphe black, smooth, extending the length of the seed.

##### Phenology.

*Miconia jashaferi* has been collected flowering and in immature fruit from July through August. Individuals have been collected in mature fruit in November and December.

##### Distribution

**Figure 4.**
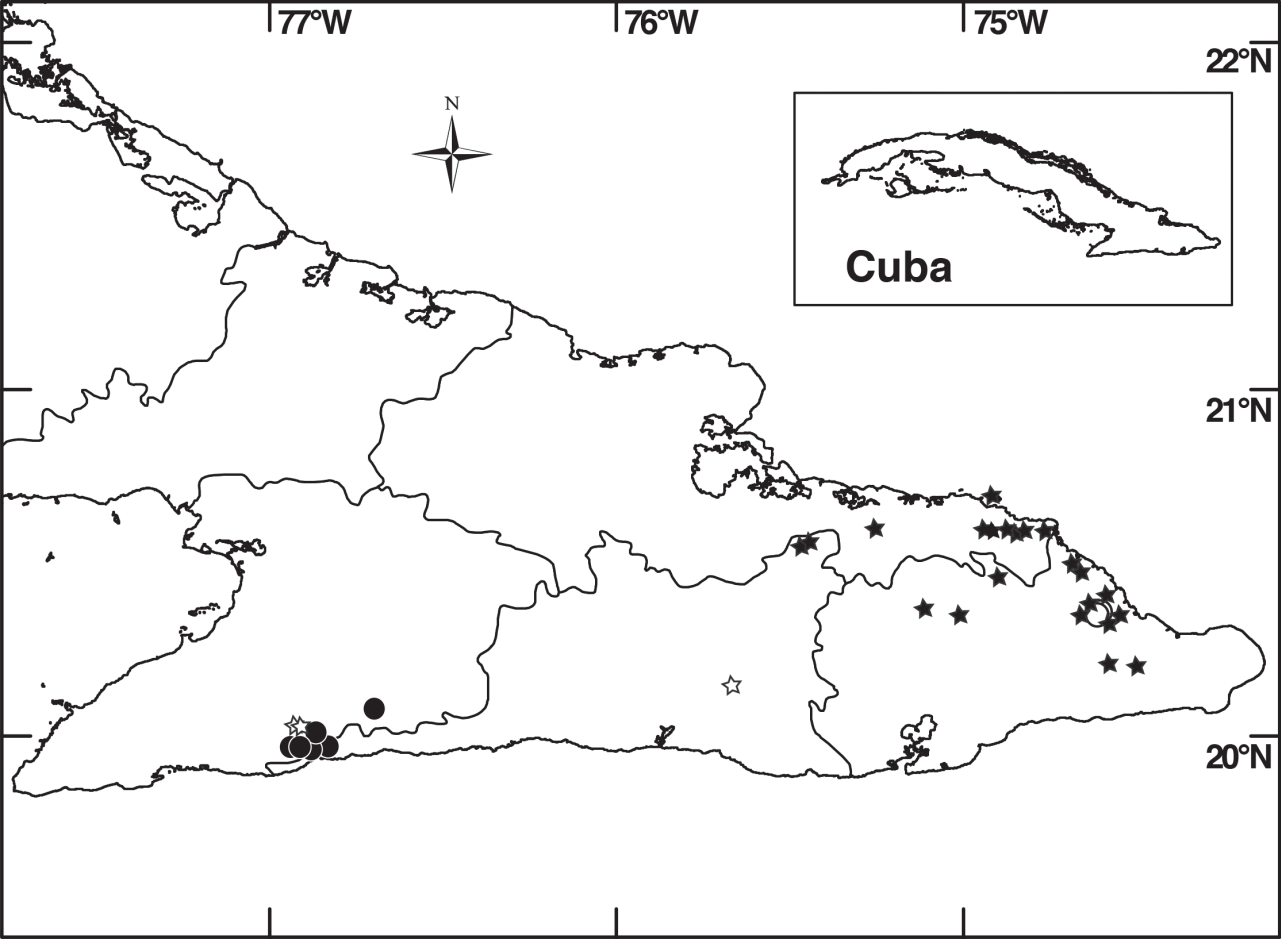
Distribution map of *Miconia hirtistyla* (closed circles), *Miconia jashaferi* (closed stars), *Miconia cubacinerea* (open circle), and *Miconia tentaculicapitata* (open stars).

(Fig. 4). *Miconia jashaferi* is known from the mountains of northeastern Cuba, including the Baracoa, Moa and Toa regions, as well as the Sierra de Cristal.

##### Ecology.

*Miconia jashaferi* grows in thorny, xerophytic scrub and semidry montane rainforest on serpentine soils from 110 – 800 m in elevation. Some associated melastomes include *Miconia baracoensis* Urb., *Pachyanthus reticulatus* Britton & P.Wilson, *Miconia moensis* (Britton) Alain, Mecranium integrifolium Triana subsp. alainii Skean and *Miconia walterjuddii* Bécquer & Michelangeli.

##### Conservation status.

*Miconia jashaferi* is a locally widespread species in eastern Cuba (Fig. 4) and is known from numerous locations from a range of elevations and forest types. Bécquer (2007) regarded the species as threatened; the species is found in both Parque Nacional Alejandro de Humboldt and Parque Nacional Pico Cristal. Hoewever, widespread mining practices around the Moa-Toa region may negatively affect localized populations of this species. Likewise, populations are highly fragemented and occupy less than 500 km^2^, so we suggest this species have a preliminary conservation assessment of endangered.

##### Discussion.

*Miconia jashaferi* is resolved as sister to the rest of the *Lima* clade (Fig. 2) in our phylogenetic analyses. However, three other species likely form a clade with *Miconia jashaferi* (*Miconia cubacinerea*, *Miconia hirtistyla*, and *Miconia tentaculicapitata*). All four of these species share the characters of large foliaceous bracts subtending the flowers, long calyx teeth, long, filiform hairs produced on the adaxial surface of the calyx tube, 3-carpellate ovaries and relatively large fruit compared with the rest of the members of the clade. *Miconia jashaferi* has often been confused with *Miconia ovatifolia* (Urb.) Judd, Bécquer & Majure (=*Ossaea ovatifolia* Urb.) of the *Paralima* clade (Majure et al. 2015), as the adaxial leaf indumentum is somewhat similar, consisting of poorly developed bulla-based hairs (Figs 3, 9A–D). These two taxa are easily separated by hair type, in that *Miconia ovatifolia* has globular-stellate hairs on most surfaces of the plant and axillary, as well as obviously terminal inflorescences.

The stem indumentum of *Miconia jashaferi* initially is reddish or purplish and then quickly turns white, in what appears to be the death of the hairs produced, which are eventually shed entirely.

##### Specimens examined.

**CUBA. Prov. Guantánamo**: near Laguna del Galano, Sierra del Frijol, La Alegría, Toa, 2 Jan 1954, *Alain 3838* (GH, HAC, NY); 19 km S of Baracoa, Vía Azul, 14 Jan 1956, *Alain 5139* (HAC, GH); Sierra Azul, Quibiján, Baracoa, ca. 500 m, 4 Jan 1960, *Alain & López-Figueiras 7294* (HAC); Sierra Azul, Quibiján, Baracoa, 4 Jan 1960, *Alain & López-Figueiras 7345* (HAJB); Baracoa. Meseta de la Iberia, camino entre el antiguo campamento minero hasta la laguna, 700 msm, 15 Apr 1985, *Álvarez & al. HFC-55897* (B, HAJB, JE); Baracoa. Pluvisilva al sur de la loma del Yunque, 300-400 msm, Jun 1967, *Bisse & Rojas HFC-2668* (HAJB, JE); Baracoa. Cerca del aserrío Nuevo Mundo, 28 Aug 1971, *Bisse HFC-19574* (HAJB, JE); Palenque. Cuchillas de Toa. Cayo Fortuna, pluvisilva cerca del arroyo Manajú, 30 Mar 1972, *Bisse & Berazaín HFC-22697* (HAJB, JE); Palenque. Cuchillas de Toa, Cayo Fortuna, charrascos cerca del arroyo Manajú, 5 Apr 1972 *Bisse & Berazaín HFC-22018* (HAJB, JE); Baracoa. Valle al noroeste del Yunque de Baracoa, Feb 1968, *Bisse & Köhler HFC-5206* (HAJB, JE); Orillas del río Baez, cerca del campamento “Los Naranjo” 1-3 Aug 1975, *Bisse & al. HFC-26994* (HAJB, JE); Baracoa. Camino de Los Naranjos a la loma de Buenavista, 21 Jan 1977, *Bisse & al. HFC-33804* (B, HAJB, JE); Baracoa. Quibiján. Orilla norte del Toa entre la desembocadura del Quibiján y del Jaguaní, 19 Feb 1978, *Bisse & al. HFC-37099* (B, HAJB, JE); Baracoa: orillas del río Duaba cerca de Vega de la Palma, 21 Feb 1978, *Bisse & al. HFC-37176* (B, HAJB, JE); Sierra de Imías. Loma de la Maestra cerca de Yamagua, 16 Feb 1979, *Bisse & al. HFC-39514* (B, HAJB, JE); Imías. Sierra de Imías. Charrascos y pinar en la cima de la loma Majagua hueca, 700 msm, 16 Apr 1984, *Bisse & al. HFC-53233* (B, HAJB, JE); Sierra Maestra (*sic*), Minas de Iberia (ad Taco Bay), ca. 800 m, 7–8 Dec 1914, *Ekman 3849* (S); Sierra de Iberia, Taco Bay, 11 Apr 1960, *López-Figueiras UO-684* (HAC, HAJB); Sierra de Iberia, Taco Bay, 11 Apr 1960, *López-Figueiras UO-705* (HAC); Sierra de Iberia, entre la base y el río Iberia, Taco Bay, 25 Jul 1960, *López-Figueiras UO-2191* (HAC, HAJB); Entre el río y la cumbre de La Iberia, Taco bay, 25 Jul 1960, *López-Figueiras UO-2237* (HAC, HAJB); Camp Toa to Camp La Barga, 400–450 m, 22–26 Feb 1910, *Shafer 4143* (US); Baracoa, Alturas de Baracoa, Mina Amores, ca. 24 km from Baracoa, bridge at entrance to mine area near where río Camarones & río Baez meet; 20°25.484'N, -74°37.202'W, 110 m, 21 Jun 2002, *Skean 4165* (FLAS). **Prov. Holguín**: Cayo Chico (Coco), Moa, 15 Apr 1945, *Acuña 12633* (HAC, NY); Monte La Breña, Moa, Oriente, 5 Nov 1945, *Acuña 13275* (HAC, NY); Monte La Breña, Moa, Oriente, 5 Nov 1945, *Acuña 13276* (HAC); Monte La Breña, Moa, Oriente, 5 Nov 1945, *Acuña 13277* (HAC); Near Cayoguan River, Moa region, 13–14 Jul 1949, *Alain 871* (GH, NY); Charrascos de la mina La Melba, 27 Apr 1973, *Álvarez & Berazaín HFC-24074* (HAJB); Moa: La Melba, charrascales cerca del aserrío, Nov 1969, *Bisse HFC-15391* (HAJB, JE); Moa: camino desde Moa hacia La Melba, 21 Dec 1968, *Bisse & Lippold HFC-11382* (HAJB, JE); Moa: La Melba, pluvisilva de montaña cerca del aserrío, 500 msm, 27 Dec 1968, *Bisse & Lippold HFC-11532* (HAJB, JE); Moa: Charrascales al oeste de Yamaniguey, Jun 1967, *Bisse & Rojas HFC-3208* (HAJB, JE); Moa: La Breña camino cerca del río Limones, 400 msm, 24 Apr 1981, *Bisse & al. HFC-45062* (B, HAJB, JE); Mina Delta, Moa region, 21 Jul 1944, *Clemente NSC-4056* (HAC, IJ, NY); Monte La Breña, Moa, Aug 1945, *Clemente et al. 4706b* (HAC); Camino nuevo de las minas de Cayoguán, Jul 1949, *Clemente et al. 6778* (HAC); Baracoa, valle del río Toa, arriba de Baracoa, 30 Jan 1971, *Grudzinskaya 764* (HAC); La Breña Woods, Moa region, Oriente, 1 Aug 1945, *León LS-22578* (GH, HAC, NY); Moa. Parque Nacional Alejandro de Humboldt, carretera a La Melba, km 28, 14 nov 2013, *Michelangeli et al. 2262* (NY); Moa. Subida a Santa Teresita, 2 km E de Yamaniguey y de alli subida hacia el S-SW 3.5-5.5 km, 20.32.13.N -74.45.46 W, 390-440 msm, 16 Nov 2013, *Michelangeli & al. 2284* (HAJB, NY); Moa, 1 Dec 1942, *Montero 21295* (HAC, NY); Moa. Alrededores de la Melba, 2 Apr 1990, II Expedición Bot. Nac. “J. Bisse” *HPR-6398* (HAJB); Moa-Baracoa, Nov 1965, *Yeno 1087* (HAC). **Prov. Santiago de Cuba.** Falda sur de la Sierra de Cristal, 28 Dec 1955, *Alain & López-Figueiras 4726* (HAC, HAJB). South of Sierra de Cristal, 28 Dec 1955, *Alain 4774* (GH, HAC, HAJB); S slopes of El Cristal, 2–7 Apr 1956, *Alain 5489* (GH, HAC, HAJB); Sierra del Cristal: falda sur de la sierra, cabezadas del río San Miguel, 600-800 msm, Apr 1968, *Bisse & Köhler HFC-8179* (HAJB, JE); Mayarí Arriba: Sierra Cristal, orillas del arroyo Cristal, 640 msm, 20 Feb 1976, *Bisse & al. HFC-30339* (B, HAJB, JE).

#### 2. Miconia hirtistyla

Taxon classificationPlantaeMyrtalesMelastomataceae

Majure & Judd, PhytoKeys 33: 69. 2014.

Fig. 5

##### Type.

CUBA. Southern Oriente and Pico Turquino, high [Sierra] Maestra, July 1922, *Hno. León LS-10923* (holotype: NY! [NY01101267]; isotypes: GH!, HAC!).

##### Description.

Evergreen shrub (height unknown); stems round in cross section, not ridged, the internodes 0.4–3.3 cm long, stem indumentum of bulla-based hairs to 1.6 mm long, these shaggy, spreading to slightly descending; nodal line absent. Leaves opposite, decussate, ovate to elliptic, not falcate, 1.6–8.2 × 1.4–3.9 cm, slightly to strongly anisophyllous (larger leaves at a node to twice as large as the smaller leaf), dark brown when dried, apex broadly acute, base broadly acute to rounded, margin dentate, the dentations obscure, each covered in one large bulla-based hair, venation acrodromous, 7-veined, the midvein and 3 pairs of arching secondary veins, secondary veins mostly basal, the innermost pair, suprabasal, produced 3–9 mm from leaf base, positioned 2.5–11 mm in from margin at widest point of blade, tertiary veins percurrent, more or less perpendicular to midvein, 1.5–4.1 mm apart at midleaf, intertertiary veins present, tertiary veins often joined by quaternary veins; adaxial leaf surface with primary, secondary and tertiary veins impressed, quaternary veins obscure, abaxial surface with all veins conspicuously raised; adaxial leaf surface covered in well developed but narrow bulla-based hairs mostly but not entirely covering the leaf areoles, widest hair bases to 0.8 mm, apices of bulla-based hairs mostly erect to recurved, sessile, glandular, hairs produced along the primary, secondary, tertiary, and quaternary veins between the bulla-based hairs; abaxial leaf surface covered in bulla-based hairs, these mostly erect with undulate apices, those along the primary, secondary, and tertiary veins spreading and larger than hairs produced throughout the lamina, lamina appearing as a series of pits from depressions of the bulla-based hairs produced from the upper leaf surface, sessile, black, glandular hairs produced along all major and minor veins, domatia of multicellular, elongate hairs, abundant in axils of primary and secondary veins, as well as the axils of the primary and secondary with tertiary veins; petioles 0.4–1.8 cm long, covered in spreading, bulla-based hairs on both surfaces. Inflorescences terminal, cymose, 2–5 flowered, flowers mostly produced in glomerulate clusters, 1.3–2.4 × 1.2–3.8 cm, the peduncle 0.6–1.3 cm long, proximal inflorescence branches 0.8–1.1 mm long, pedicels absent; bracts ovate to elliptic, foliaceous, 5–17 mm long; bracteoles foliaceous, elliptic, 2.8–4.3 × 1.7–2.1 mm, covered in bulla-based hairs marginally and abaxially and glabrous abaxially or with filiform hairs towards the base. Flowers 6-merous, sessile. Hypanthium 2.6–3.2 mm long, short-oblong to globose, unlobed, slightly constricted below the torus, free portion of the hypanthium 1–1.4 mm long, abaxial surface covered in bulla-based hairs to 2.3 mm long, and occasional, sessile, glandular hairs near the bases of the bulla-based hairs; adaxial surface (i.e., free portion) covered in small, bulla-based hairs; calyx teeth 6, 4.5–4.6 × 0.2–0.4 mm, ascending or spreading, covered in bulla-based hairs; calyx lobes 6, more or less triangular, apex acute, 1–1.4 × 1–1.5 mm, covered in bulla-based hairs abaxially and gland-headed, filiform hairs adaxially; calyx tube not tearing, 0.3–0.5 mm long with bulla-based hairs abaxially and sessile, glandular hairs, as well as filiform, gland-headed hairs adaxially and along the apex of the tube; petals 6, most likely white, elliptic to obovate, 5.7–6.6 × 2.7–3.1 mm, with an acuminate apex, only slightly to conspicuously clawed, with one slightly bulla-based hair produced abaxially, subapically, or in some cases, marginally, to 0.1 mm long; stamens 12; filaments 3.8–4.1 mm long, glabrous, anthers 2.2–2.6 mm long, ovate, with one apically oriented pore, anther thecae 2–2.5 mm long, anthers without a dorso-basal appendage; style 3.8–4.4 mm long, pubescent with slightly bulla-based hairs, oblong to only slightly dilated in the middle, collar absent, style subtended by multicellular, linear to elongate-triangular (needle-like) hairs, which grade into the surrounding bulla-based hairs of the ovary apex, stigma punctate; ovary 1.2–2.8 × 1.5–2.5 mm, apex convex, pubescent with bulla-based hairs, placentation axile, placenta apparently not deeply intruded, 3-locular; berries not seen, mature seeds not seen.

**Figure 5.**
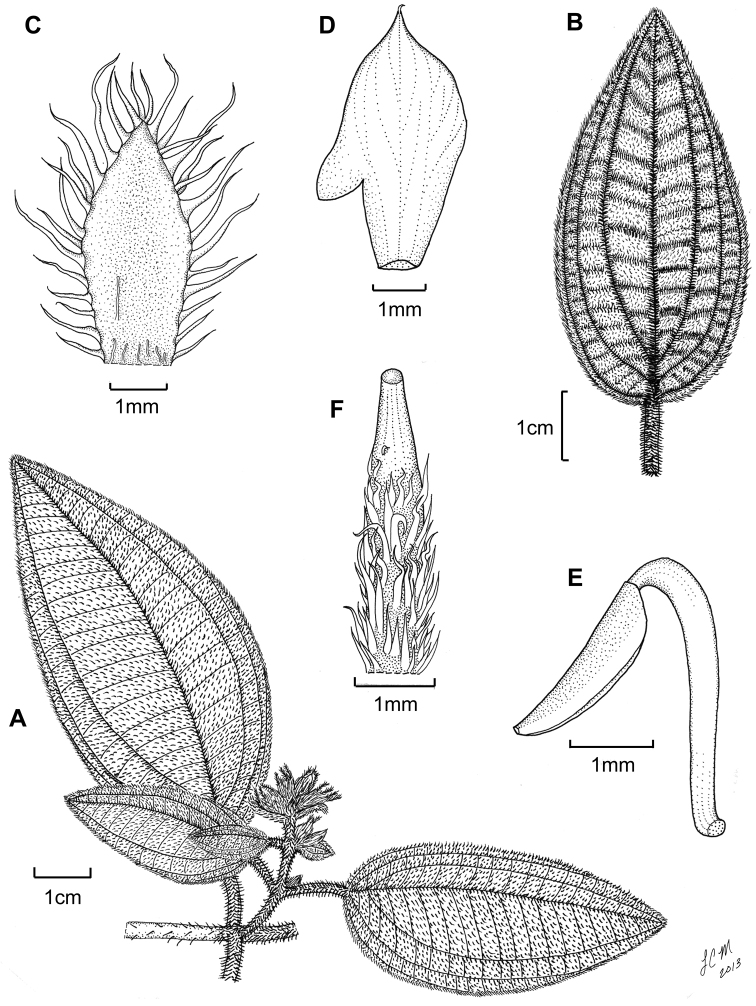
*Miconia hirtistyla* **A** habit (*Ekman 14617*) **B** leaf abaxial surface (*León LS-10923*) **C** bracteole (*León LS-10923*) **D** petal (*Ekman 14617*) **E** stamen (*Ekman 14617*) **F** style (*Ekman 14617*). Reproduced with permission from Majure et al. (2014a).

##### Phenology.

*Miconia hirtistyla* was collected in bud, at anthesis, and in immature fruit in March and July.

##### Distribution

(Fig. 4). *Miconia hirtistyla* is only known from the western Sierra Maestra, Cuba.

##### Ecology.

*Miconia hirtistyla* occurs in montane rainforest, pine forest and elfin forest on rocky soils at elevations of 700–1800 m. Associated melastomes include *Miconia argentimuricata* Majure & Judd, *Miconia norlindii* (Urb.) Majure & Judd and *Miconia nystroemii* Urb.

##### Conservation status.

*Miconia hirtistyla* is mostly known from the very, well protected forests of Parque Nacional Pico Turquino. Although the species has not been collected recently, and we know nothing regarding its reproductive biology or population numbers, it is most likely not threatened by anthropogenic disturbance and habitat loss. However, at present we must categorize the species as data deficient, owed to the lack of data. Fieldwork will be necessary to appropriately assess the conservation status of this species.

##### Discussion.

Although generally confused with *Miconia jashaferi*, *Miconia hirtistyla* is most phenetically similar to *Miconia cubacinerea*, and the two species may be sister taxa. The two species differ in abaxial leaf surface indumentum and the degree of pitting on the abaxial leaf surface, calyx teeth length (i.e., 4.5–4.6 mm in *Miconia hirtistyla* vs. 5.7–6.2 mm in *Miconia cubacinerea*), petal form (i.e., clawed in *Miconia hirtistyla*), and style indumentum (i.e., pubescent styles in *Miconia hirtistyla*).

##### Specimens examined.

**CUBA. Prov. Granma**: A lo largo del camino de Minas del Frio a Montpie, 23 Apr 1978, *Bisse et al. HFC-37347* (B, HAJB, JE); Valle del arroyo Escondido, 700–1000 msm, 26 Apr 1978, *Bisse et al. HFC-37628* (B, HAJB, JE); Bartolomé Masó. Estribo del Pico Turquino, 20 Apr 1979, *Bisse et al. HFC-40517* (B, HAJB, JE); Manguito, pinares de la loma La Botella, 1200–1400 msm, 22 Mar 1970, *Lippold HFC-16283* (HAJB, JE). **Prov. Santiago de Cuba**: Oriente, Pico Turquino, 12–26 Jul 1936, *Acuña SV-10189* (HAC); Oriente, Sierra Maestra, Cima del Pico Turquino, 10 July 1936, *Acuña SV-22705* (HAC); Oriente, Sierra Maestra, steep rocks of Loma Regino, 25 Jul 1922, *E.L. Ekman 14617* (S); southern Oriente and Pico Turquino, high [Sierra] Maestra, Jul 1922, *Fre. León LS-10927* (GH, NY). **Prov. Granma/Santiago de Cuba.** Oriente, date not given, *Acuña SV-15144* (HAJB).

#### 3. Miconia cubacinerea

Taxon classificationPlantaeMyrtalesMelastomataceae

Majure & Judd, J. Bot. Res. Inst. Texas 7: 268. 2013.

Fig. 6A

Clidemia cinerea Griseb., Cat. Pl. Cub. [Grisebach] 97. 1866. Type: CUBA. [Guantánamo]. Yunque de Baracoa, 11 Jun 1860–1864, *C. Wright 2483* (holotype: GOET! [GOET007034]; isotypes: BM! [BM000884493], BR! [BR0000005185191], G! [G00353604], GDC! [G00316293], GH! [GH00072059], K! [K000535607], MO! [MO-2049513], YU! [YU065014]).
Oxymeris cinerea (Griseb.) Triana, Trans. Linn. Soc. London 28: 92. 1871. Type: Based on Clidemia cinerea Griseb.
Leandra cinerea (Griseb.) Cogn., Fl. Bras. (Martius) 14: 71. 1886. Type: Based on Clidemia cinerea Griseb.
Maieta cinerea (Griseb.) M.Gómez, Anales Hist. Nat. 23: 71. 1894. Type: Based on Clidemia cinerea Griseb.

##### Type.

Based on *Clidemia cinerea* Griseb. (non *Miconia cinerea* Cogn. in Mart., Fl. Bras. (Martius) 14, pt. 4: 290. 1887).

##### Description.

Evergreen shrub, 2 m tall; stems round in cross section, not ridged, the internodes 0.4–2.4 cm long, stem indumentum of spreading, bulla-based hairs to 1.7 mm long; nodal line absent. Leaves opposite, decussate, elliptic, 3.9–7.2 × 1.7–3.6 cm, slightly anisophyllous, apex acute to obtuse, base acute to rounded, venation acrodromous, 5-veined, the midvein and 2 pairs of arching secondary veins, secondary veins mostly suprabasal, the innermost pair produced 0.4–0.8 mm from leaf base, positioned 0.25–0.6 mm in from margin at widest point of blade, tertiary veins percurrent, more or less perpendicular to midvein, 0.2–0.48 mm apart at midleaf, impressed and inconspicuous on the upper leaf surface, intertertiary veins present, conspicuous on lower leaf surface, tertiary veins often joined by quaternary veins; adaxial leaf surface covered in poorly developed, bulla-based hairs, widest hair bases to 0.5 mm, apices of bulla-based hairs mostly recurved, young leaf adaxial surface producing long-stemmed, clavate-dentritic hairs at the leaf base and apex, sessile, glandular hairs produced throughout the lamina between the bulla-based hairs; abaxial leaf surface sparsely covered in poorly developed bulla-based hairs, these erect, those along the primary, secondary, and tertiary veins larger than hairs produced throughout the lamina, the lamina easily visible, sessile, glandular hairs produced throughout the lamina between the bulla-based hairs; domatia of multicellular, elongate hairs prominent in the axils of the primary and secondary and primary and tertiary veins; petioles 0.3–1.1 cm long, covered in spreading, bulla-based hairs on both surfaces. Inflorescences terminal, 1–3 flowered, flowers produced in glomerulate clusters, 0.8–1.2 × 1–1.4 cm, the peduncle absent to 0.1 cm long, pedicels absent; bracts elliptic to rotund, 3–6.5 mm long; bracteoles elliptic to rotund, 3–4 × 2–2.4 mm, covered abaxially in bulla-based hairs, mostly glabrous abaxially but with bulla-based hairs produced at the base. Flowers 5-merous, sessile; hypanthium ca. 3.4 mm long, oblong, unlobed, barely constricted below the torus, free portion of the hypanthium ca. 1.7 mm long, abaxial surface covered in bulla-based hairs to 3.2 mm long; adaxial surface (i.e., free portion) covered in small, bulla-based hairs; calyx teeth 5, 5.7–6.2 × 0.6–0.7 mm, spreading, covered in bulla-based hairs, as well as a few clavate-dendritic hairs; calyx lobes triangular, apex rounded, ca. 0.6 × 1.5 mm, covered in bulla-based hairs abaxially and filiform hairs at the apex adaxially; calyx tube not tearing, ca. 0.3 mm long with bulla-based hairs abaxially and filiform hairs at the apex adaxially; petals 5, size at anthesis unknown, only seen in bud, white (according to *Wright 2483*), with one slightly bulla-based hair produced abaxially, subapically, hairs to 0.4 mm long; stamens 10; filaments (immature) ca. 2.5 mm long, anthers (immature) ovate, 2–2.1 mm long, with one apically oriented pore, anther thecae 1.7–1.9 mm long, anthers lacking dorso-basal appendage; style (immature) ca. 3.5 mm long, glabrous, only slightly dilated in the middle, collar absent, style subtended by multicellular, linear (needle-like) hairs, which are the same as the hairs of the ovary apex, stigma punctate; ovary ca. 2.3 × 2.2 mm, apex convex, pubescent with linear hairs, placentation axile with shallowly intruded placenta, 3-locular; berries globose, 3.8 mm long (immature and including calyx tube), 7 mm wide; seeds not seen.

**Figure 6.**
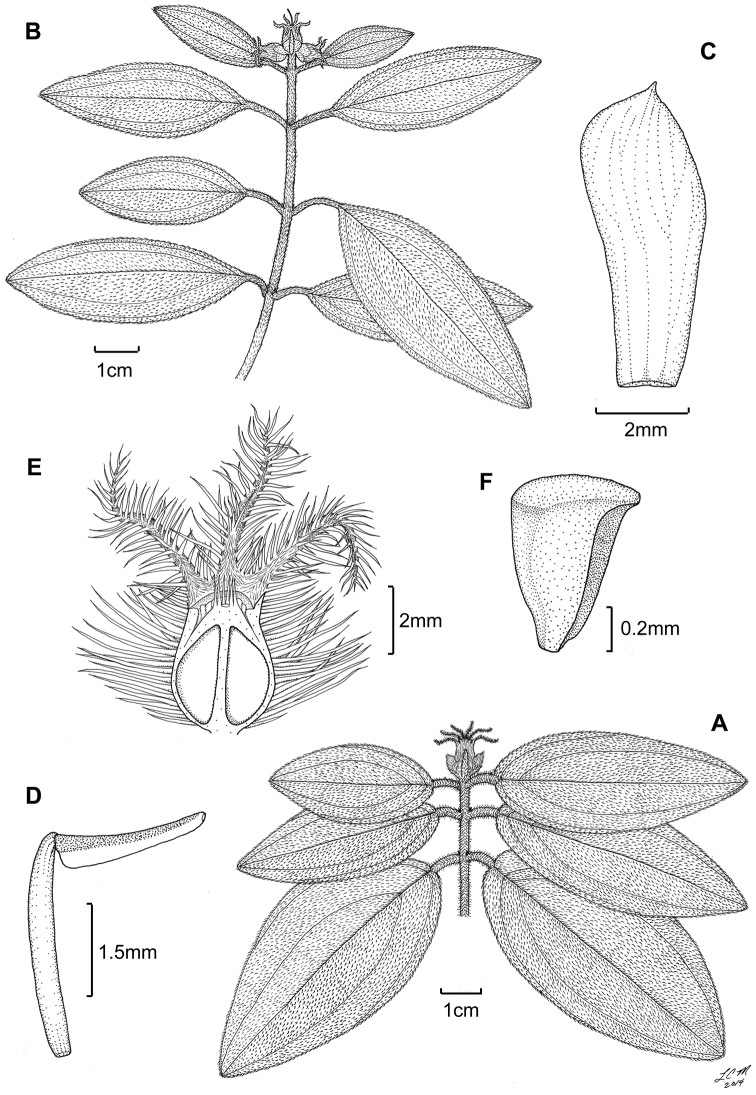
Illustration of *Miconia cubacinerea* (**A**) and *Miconia tentaculicapitata* (**B–F**). **A** Habit of *Miconia cubacinerea* from the type specimen (*Wright 2483*) **B** habit of *Miconia tentaculicapitata* (*Ekman 14823*) **C** petal (*Hioram 7218*) **D** stamen (*Hioram 7218*) **E** fruit longitudinal section (*Hioram 7218*), and **F** seed (*León LS-12337*).

##### Phenology.

The type collection of *Miconia cubacinerea* was gathered in bud in June.

##### Distribution

(Fig. 4). *Miconia cubacinerea* is only known from the Elemento Natural Destacado Yunque de Baracoa region of Cuba, based upon the type gathering and one other collection from that area.

##### Ecology.

Little information is available regarding the ecology of this species. However, the vegetation of Yunque de Baracoa is primarily evergreen sclerophylous shrubland over limestone. *Miconia cubacinerea* likely forms part of the very humid interior karstic formation of Yunque de Baracoa, according to *Alain & Acuña 7538*. Associated melastomes include *Clidemia wrightii* Griseb., *Conostegia lindenii* Cogn., *Miconia yunquensis* Judd, Bécquer & Majure and *Miconia heterophylla* (Naudin) M. Gómez.

##### Conservation status.

This species in only known from the type specimen and one other gathering from 1960, thus we consider that is it data deficient, as it has not been collected from the Yunque de Baracoa area since that time. More fieldwork will be necessary to fully evaluate the conservation status of *Miconia cubacinerea*.

##### Discussion.

*Miconia cubacinerea* is most phenetically similar to *Miconia hirtistyla*, but the two species differ in leaf indumentum, the degree of pitting on the abaixal leaf surface, calyx teeth length, petal form (i.e., non-clawed in *Miconia cubacinerea*), and style indumentum (i.e., glabrous in *Miconia cubacinerea*); see also discussion under *Miconia hisrtistyla*.

##### Specimens examined.

**CUBA. Prov. Guantánamo**: Interior del Yunque de Baracoa, Yunque de Baracoa, 500 m, 14 Jan 1960, *Alain & Acuña 7538* (HAC, HAJB).

#### 4. Miconia tentaculicapitata

Taxon classificationPlantaeMyrtalesMelastomataceae

Majure & Judd, J. Bot. Res. Inst. Texas. 7: 269. 2013.

Fig. 6B–F

Ossaea capitata Urb., Repert. Spec. Nov. Regni Veg. 22: 237. 1926. Type: CUBA. Arroyo del Cristo (tributary of Yara), Sierra Maestra, south of Nagua, 7 Aug 1922, *E L. Ekman 14748* (lectotype: S! [S05-3771], designated here; isolectotypes: G! [G00353943], NY! [NY00099635, NY00099636]).

##### Type.

Based on *Ossaea capitata* Urb.

##### Description.

Evergreen shrub, height unknown; stems round in cross section, not ridged, the internodes 0.7–5.3 cm long, stem indumentum of ascending, bulla-based hairs to 0.9 mm long; nodal line present. Leaves opposite, decussate, elliptic, 2.2–7.3 × 1–3.1 cm, slightly anisophyllous, apex acute or rounded, base acute to rounded, margins dentate, but dentations obscured by large bulla-based hair, venation acrodromous, 5-veined, the midvein and 2 pairs of arching secondary veins, secondary veins basal to suprabasal, the innermost pair suprabasal, produced 1.5–6 mm from leaf base, positioned 1.9–5 mm in from margin at widest point of blade, tertiary veins percurrent, more or less perpendicular to midvein, 1.1–3.5 mm apart at midleaf, intertertiary veins present, tertiary veins often joined by quaternary veins; adaxial leaf surface covered in well-developed bulla-based hairs, these dense, meeting at their bases and covering the leaf areoles, widest hair bases to 1.5 mm, apices of bulla-based hairs mostly recurved towards the leaf apex or margin, young leaf adaxial surface producing occasional long-stemmed, clavate-dentritic hairs along the primary an secondary veins, sessile, glandular hairs produced along the primary, secondary, tertiary, and quaternary veins between the bulla-based hairs; abaxial leaf surface covered in bulla-based hairs, these erect, spreading or ascending, those along the primary, secondary, and tertiary veins larger than hairs produced throughout the lamina, the lamina clearly visible, appearing as a series of pits from depressions of the bulla-based hairs produced from the upper leaf surface, sessile, glandular hairs produced throughout the lamina and along veins; petioles 0.4–1.5 cm long, covered in ascending, bulla-based hairs on both surfaces. Inflorescences terminal, 3–5 flowered, flowers produced in glomerulate clusters, 0.8–1.4 × 1.5–2.5 cm, the peduncle absent or to 0.4 cm long, proximal inflorescence branches absent, pedicels absent; bracts oblong or elliptic, 4.2–11 mm long; bracteoles ovate, rotund to broadly elliptic, 3–4 × 4.3–6.5 mm, covered in bulla-based hairs abaxially. Flowers 6–7-merous, sessile; hypanthium 4–5 mm long, short-oblong to globose, unlobed, slightly constricted below the torus, free portion of the hypanthium 0.8–1.5 mm long, abaxial surface covered in bulla-based hairs from 2.7–4 mm long, and occasional, sessile, glandular hairs near the bases of the bulla-based hairs; adaxial surface (i.e., free portion) covered in bulla-based hairs; calyx teeth 6–7, 5–8 × 0.5–0.8 mm, spreading to recurved, covered in bulla-based hairs; calyx lobes more or less triangular, apex acute, 2.2–2.7 × 0.9–1 mm, covered in bulla-based hairs abaxially and long, filiform hairs adaxially; calyx tube not tearing, 0.2–1.4 mm long with bulla-based hairs abaxially and long, filiform hairs adaxially; petals 6–7, color unknown, oblong to obovate, 5.7–6.7 × 2.6–2.7 mm, with an acuminate apex and membranous margin, without bulla-based hairs produced abaxially; stamens 8; filaments 3.2–4 mm long, glabrous, anthers 2.1–2.8 mm long, with one apical to ventrally oriented pore, anther thecae 2.3–2.6 mm long, anthers without a dorso-basal appendage; style 6.2–6.6 mm long, glabrous, not dilated in the middle, collar absent, style subtended by a crown of multicellular, linear to elongate-triangular (needle-like) hairs, which are slightly longer than the surrounding bulla-based hairs of the ovary apex, stigma punctate; ovary 4–4.9 × 3.5–4.5 mm, apex convex, glabrous, except for the linear or elongate-triangular hairs forming crown, placentation axile with shallowly intruded placenta, 3-locular; berries globose, purple at maturity, 6–7 mm long (including calyx tube), 5–6 mm wide, seeds 0.7–0.8 mm long, obpyramidal, testa smooth, light brown, raphe black, smooth, extending the length of the seed.

##### Phenology.

Specimens flowering, with immature and mature fruit have been collected in July and August.

##### Distribution

(Fig. 4). *Miconia tentaculicapitata* is restricted to the Sierra Maestra of Cuba. It has been collected primarily in two localities, surrounding the Yara River (along Arroyo el Cristo) in the Granma province and from Loma del Gato in the Santiago de Cuba province.

##### Ecology.

Very little information is available regarding the ecology of *Miconia tentaculicapitata*, but collections of Ekman (*14748*, *14823*) were both from along streams (“on rocks along the river”), so this species likely inhabits moist, rocky outcrops in riparian habitats.

##### Conservation status.

Insufficient data are available for a critical evaluation of the conservation status of *Miconia tentaculicapitata*, thus we suggest a designation of data deficient (DD) according to IUCN (2012) criteria. However, the areas of the Sierra Maestra from where the type collection was made (*Ekman 14748*) are contained within Turquino National Park, and the species also is found with the Reserva Ecológica (RE) Loma del Gato-Monte Líbano. Therefore, this species is likely protected from habitat loss and any major anthropogenic disturbance.

##### Discussion.

*Miconia tentaculicapitata* is most likely closely related to *Miconia jashaferi*, *Miconia hirtistyla*, *Miconia cubacinerea*, as the four species share several morphological characters (e.g., foliaceous bracts and bracteoles, long, filiform hairs on the adaxial calyx tube surface, and 3 locular ovaries). The specimens *Linden 2102* (BR, K, P), which are the basis for *Miconia lima* in Cuba (Alain 1957), were misidentified and actually are *Miconia tentaculicapitata*. However, one of the two specimens of *Linden 2102* at BR (13239718) actually contains a fragment of a leaf of *Miconia lima* in the packet, most likely a contaminant from a specimen of *Miconia lima* during specimen preparation or investigation.

##### Specimens examined.

**CUBA. Prov. Granma**: Sierra Maestra, Arroyo el Cristo, one of the tributaries of Rio Yara, on rock along the river, 10 Aug 1922, *Ekman 14823* (S, US). **Prov. Santiago de Cuba**: Loma, San Juan, 1 Jul 1925, *Hioram 7218* (HAC, IJ, NY); Loma del Gato, Cobre Range of Sierra Maestra, 1 Aug 1924, *León LS-12331* (HAC, NY); Nimanima, 1843–1844, *Linden 2102* (BR, K, P). **Prov. Granma/Santiago de Cuba**. Sierra Maestra (Oriente), 10 Oct 1922, *Ekman s.n.* (HAJB).

#### 5. Miconia norlindii

Taxon classificationPlantaeMyrtalesMelastomataceae

(Urb.) Majure & Judd, J. Bot. Res. Inst. Texas. 7: 269. 2013.

Figs 7F–H , 9E–J

Ossaea norlindii Urb., Symb. Antill. (Urban) 9(1): 124. 1923. Type: CUBA. Oriente, Sierra Maestra, La Gran Piedra, supra Daiquiri, ca. 1000 m, 29 Oct 1916, *Ekman 8136* (lectotype: S! [S05-3782], designated here; isolectotypes: L! [LD1669114], US! [US00123696]).
Ossaea turquinensis Urb. Symb. Antill. (Urban) 9(1): 122. 1923. Type: CUBA. Oriente, Sierra Maestra, in silva jugo septentr. mont. Pico Turquino, ca. 1750 m, 8 Apr 1915, *Ekman 5303* (lectotype: S! [S05-3789], designated here).

##### Type.

Based on *Ossaea norlindii* Urb.

##### Description.

Evergreen shrub, 2–4 m tall; stems round in cross section, not ridged, the internodes 0.3–8.1 cm long, stem indumentum of granulate, bulla-based hairs to 0.5 mm long, these ascending, appressed, and clavate-dendritic hairs at the nodes; nodal line present. Leaves opposite, decussate, elliptic, 2.1–7.1 × 1–3.4 cm, slightly anisophyllous, apex acute to rounded, base acute to cuneate, margins dentate, the dentations covered in one large bulla-based hair, venation acrodromous, 5-veined, the midvein and 2 pairs of arching secondary veins, the outermost intramarginal or not, secondary veins mostly basal to suprabasal, the innermost pair, suprabasal, produced 2.2–16 mm from leaf base, positioned 2.1–6.1 mm in from margin at widest point of blade, tertiary veins percurrent, more or less perpendicular to midvein, 1.8–4.6 mm apart at midleaf, intertertiary veins present, tertiary veins occasionally joined by quaternary veins; adaxial leaf surface covered in dorsally compressed, bulla-based hairs, widest hair bases to 1.1 mm, apices of bulla-based hairs mostly erect, young leaf adaxial surface producing long-stemmed, clavate-dentritic hairs along the primary and secondary veins, and sessile, glandular hairs produced along the primary, secondary, tertiary, and quaternary veins between the bulla-based hairs; abaxial leaf surface covered in bulla-based hairs, these mostly ascending, those along the primary, secondary, and tertiary veins larger than hairs produced throughout the lamina, the lamina clearly visible, sessile, glandular hairs produced throughout the lamina and along all veins, domatia of tufts of long, multicellular hairs usually produced in the axils of the primary and secondary veins; petioles 0.4–1.4 cm long, covered in ascending, appressed, bulla-based hairs on both surfaces. Inflorescences terminal, 3–15 flowered, expanded cymes, 2.2–6 × 1.1–4 cm, the peduncle 0.5–3 cm long, proximal inflorescence branches 2.1–26 mm long; bracts oblong to ovate, 1–3 mm long; bracteoles ovate, 0.8–4.1 × 0.3–0.6 mm, appearing as large bulla-based hairs. Flowers 4-merous, pedicels 0.3–1 mm long; hypanthium 2.2–3 mm long, short-oblong to globose, 3–4-lobed, slightly constricted below the torus, free portion of the hypanthium 0.5–0.6 mm long, abaxial surface covered in bulla-based hairs to 0.8 mm long, and occasional, sessile, glandular hairs near the bases of the bulla-based hairs; adaxial surface (i.e., free portion) covered in small, bulla-based hairs; calyx teeth 0.7–1.6 × 0.4–0.6 mm, ascending or spreading, covered in bulla-based hairs; calyx lobes more or less triangular, apex acute, 0.8–1.5 × 1.5 mm, covered in bulla-based hairs abaxially and sessile, sparse, glandular hairs adaxially; calyx tube not tearing, 0.5–0.6 mm long with bulla-based hairs abaxially and sessile, glandular hairs adaxially, clavate dendritic hairs produced at calyx tube apex; petals 4, white, elliptic, 5.8–6.2 × 2.1–3.1 mm, with an acute apex and membranous margin, with 2–6 slightly bulla-based hairs produced abaxially, just below the apex, to 1 mm long; stamens 8; filaments 2.2–3 mm long, glabrous, anthers 1.9–2.6 mm long, with one dorsally oriented pore, anther thecae 1.2–1.8 mm long, anthers with a dorso-basal appendage 0.3–0.7 mm long; style 8.2–8.9 mm long, glabrous, not dilated in the middle, collar absent, style subtended by a crown of multicellular, linear to elongate-triangular (needle-like) hairs, which are slightly longer than the surrounding bulla-based hairs of the ovary apex, stigma punctate; ovary 1.5–1.6 × 2.2–4.6 mm, apex flat, pubescent with bulla-based hairs, except for the linear or elongate-triangular hairs forming crown, placentation axile with deeply intruded placenta, 3–4 locular; berries globose, 4-lobed to unlobed, purple at maturity, 3.1–4 mm long (including calyx tube), 3.4–4 mm wide, seeds 0.6–0.7 mm long, obpyramidal, testa smooth, light brown, raphe dark brown, smooth, extending the length of the seed.

**Figure 7.**
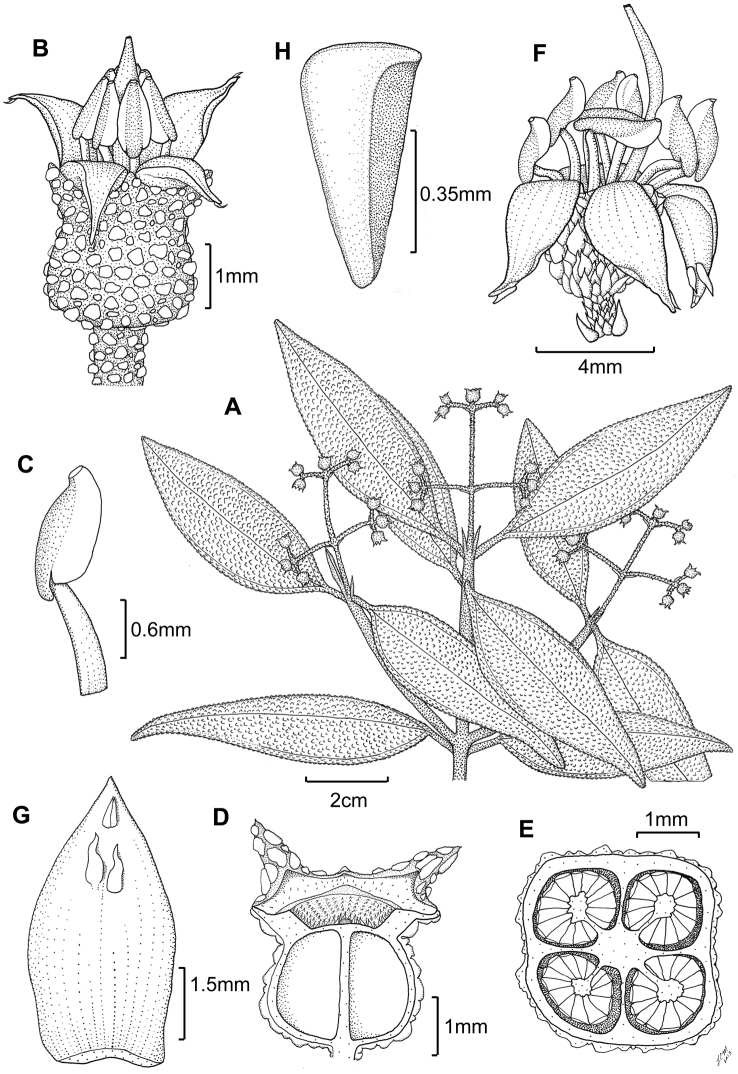
Illustration of *Miconia granulata* (**A–E**) and *Miconia norlindii* (**F–H**). **A** Habit of *Miconia granulata* from the type specimen (*Ekman 3789*) **B** flower **C** stamen **D** fruit longitudinal section **E** fruit cross section (all from *Acuña 13288*) **F** flower of *Miconia norlindii* (*Pipoly 24478*) **G** petal (*Pipoly 24478*), and **H** seed (*Clemente NSC-5814*).

##### Phenology.

*Miconia norlindii* has been collected in flower from November through February.

##### Distribution

**Figure 8.**
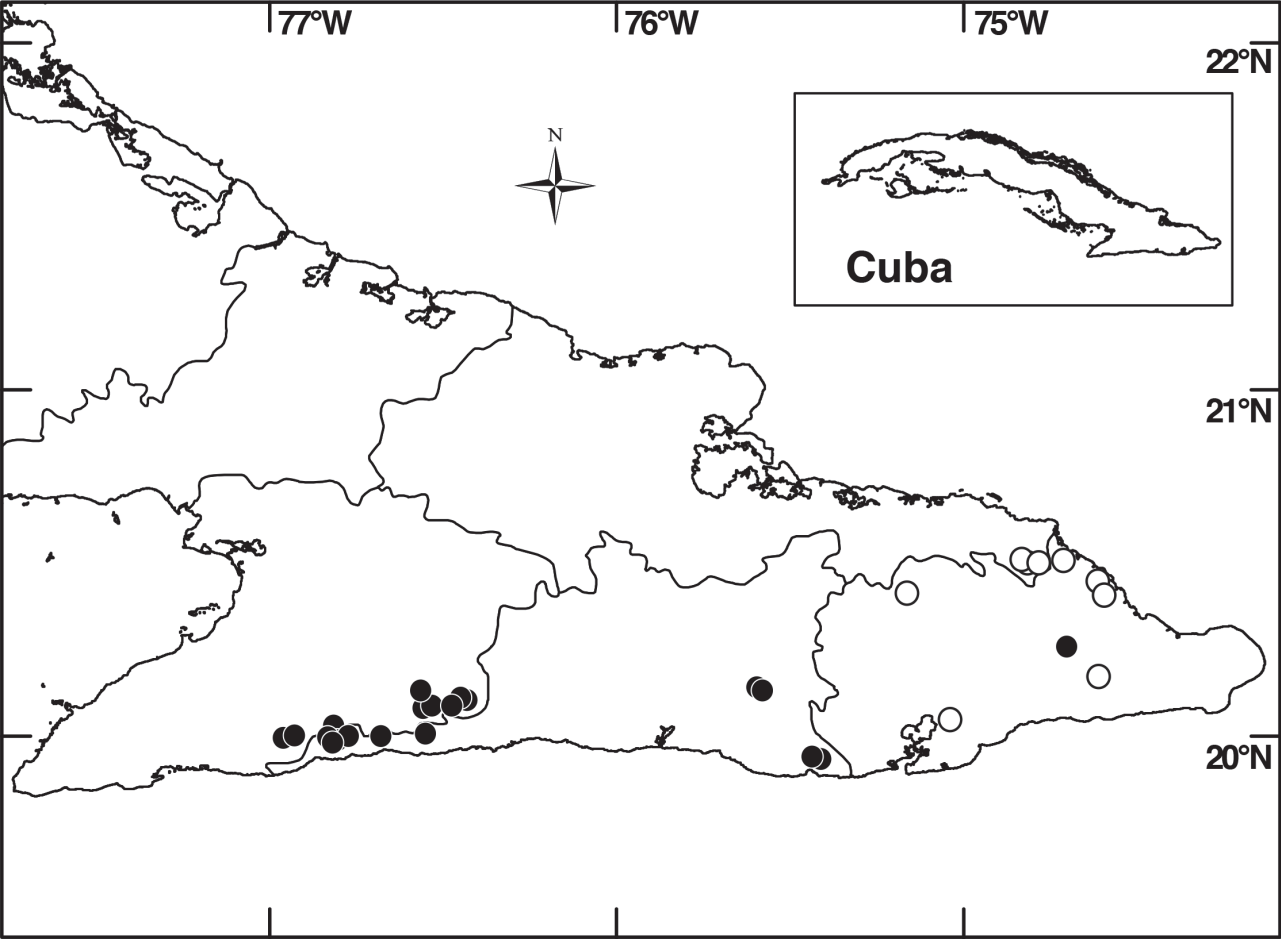
Distribution of *Miconia norlindii* (closed circles) and *Miconia granulata* (open circles).

(Fig. 8). *Miconia norlindii* is restricted to the Sierra Maestra and Sierra de Imías of eastern Cuba.

##### Ecology.

*Miconia norlindii* occurs in wet montane rainforest and cloud forests occasionally along streams from 800–1800 m. Some associated melastomes include *Pachyanthus pedicellatus* Urb., *Miconia nystroemii* Urb., *Miconia rufa* (Griseb.) Triana, *Miconia turquinensis* Urb. & Ekman, *Graffenrieda rufescens* Britton & P.Wilson and *Meriania albiflora* Carmenate & Michelangeli

##### Conservation status.

Bécquer (2007) considered *Miconia norlindii* to be threatened, however, regarding the number of localities for the species and its presence in protected areas, such as Parque Nacional Pico Turquino, Parque Nacional Pico Bayamesa, Paisaje Natural Protegido La Gran Piedra and la Reserva Ecológica Loma del Gato-Monte Líbano, it may be best to categorize this species as near threatened. Although the species is widespread, it occurs in areas highly vulnerable to climate change (high montane forests) and populations are few.

##### Discussion.

Urban described *Ossaea turquinensis* from sterile material. Based on morphological comparisons, *Ossaea turquinensis* falls into the range of variation of *Ossaea norlindii* and the two are here considered conspecific. The name *Miconia norlindii* was selected and used for this species by Majure and Judd (2013a), as both species were described in the same publication and *Miconia turquinensis* Urb. & Ekman was already occupied.

##### Specimens examined.

**Cuba. Prov. Granma**: Buey Arriba. Alrededores del poblado de Barrio Nuevo, 1400 msm, 10 May 1988, *Álvarez & al. HFC-63823* (B, HAJB, JE); IBID *HFC-63699* (B, HAJB, JE); Buey Alto de Rondón, 22 May 1988, *Álvarez & al. HFC-64989* (B, HAJB, JE); Buey Arriba. Alrededores del poblado Barrio Nuevo, 1400 msm, 15 May 1988, *Álvarez & al. HFC-64545* (B, HAJB, JE); Buey Arriba. Alto del Escudero, 20 May 1988, *Álvarez & al. HFC-64791* (B, HAJB, JE); Sierra Maestra: falda norte del Pico Bayamesa, 900–1200 msm, May 1968, *Bisse & Duek HFC-9337* (B, HAJB, JE); Bartolomé Masó. A lo largo del camino de Minas del Frio a Mont Pie, 23 Apr 1978, *Bisse & al. HFC-37324* (HAJB, JE); Bartolomé Masó: Firme de la Sierra Maestra entre Lagunitas y Aguada de Joaquín, 300-900 msm, 19 Apr 1974, *Bisse & al. HFC-40419* (HAJB); Guisa. Subida y firme del pico El Gigante, 21 Apr 1989, *Dietrich & al. HFC-67133* (B, HAJB, JE); Sierra Maestra, ad La Bayamesa, in “fangales” ca. 1300 m, 3 May 1916, *Ekman 7082* (S); Sierra Maestra, on the divide between Rio Yara and Rio Palmamocha, on the slope towards one of the highest tributaries of Rio Yara, ca. 1100 m, 16 Jul 1922, *Ekman 14343* (S); Sierra Maestra, in high mt. forest above Loma Bambí, ca. 1000 m, 8 Nov 1922, *Ekman 15656* (S, US); Sierra Maestra, El Gigante on the ridge, 1100 m, 4 Jan 1923, *Ekman 16076* (NY, S); Sierra Maestra: Pico bayamesa, falda norte, 800-1100 msm, 19 Mar 1970, *Lippold HFC-16088* (HAJB, JE); En la Maestrica, cercanias del batey del “Alto de la Valenzuela”, Sierra Maestra, 4500’, 5–8 Apr 1955, *López-Figueiras 2030* (HAC, HAJB); Zona boscosa de la Sierra Maestra, entre los arroyos Peladero e Indio, 3000-4500 pies alt., 27 Nov 1959, *López-Figueiras UO-430* (HAC, HAJB, US); Granma/Santiago de Cuba. Bartolome Masó/Guamá. Parque Nacional Turquino, sendero Alto el Naranjo-Pico Turquino, alrededor de Palma Mocha, entre Km 3 y 4, 8 Nov 2013, *Michelangeli et al. 2213* (NY); cruce de Lima, Sierra Maestra, 20 Aug 1982, *Moncada s.n.* (HAC); cruce de Lima, Sierra Maestra, 21 Aug 1982, *Moncada s.n.* (HAC); slopes of La Bayamesa, crest of the Sierra Maestra near Aserradero San Antonio de los Cumbres, 1500–1800 m, 21–24 Jan 1956, *Morton 9272* (US); Guisa, Pico El Gigante, 800–1100 m, 8 Dec 2002, *Pipoly 24466* (FTG); Guisa, Pico El Gigante, 8 Dec 2002, *Pipoly 24478* (FTG); Bartolomé Maso, St. Domingo, camino desde el Alto del Naranjo hasta La Aguada de Joaquín, Sierra Maestra, 22.371388°N, -76.848056°W, 850–1200 m, 8 Dec 2002, *Pipoly 24522* (FTG); Bartolomé Maso, St. Domingo, camino desde el Alto del Naranjo hasta La Aguada de Joaquín, Sierra Maestra, 22.371388°N, 76.848056°W, 850–1200 m, 8 Dec 2002, *Pipoly 24554* (FTG). **Prov. Guantánamo**: Imías. Sierra de Imías. Cabezadas del arroyo Los Cacaos. 600-700 msm, 7 Apr 1984, *Bisse & al. HFC-52409* (B, HAJB, JE); **Prov. Santiago de Cuba**: summit of Pico Turquino, Sierra Maestra, 1960 ft, 1–2 Aug 1935, *Acuña 6756* (HAC, NY); Pico Turquino, 10–26 Jun 1936, *Acuña 7714* (HAC); cima del Pico Turquino, Sierra Maestra, 1–2 Aug 1935, *Acuña 9645* (HAC); Pico Turquino, 12–26 Jul 1936, *Acuña 10193* (HAC); cima del Pico Turquino, Sierra Maestra, 1 Aug 1935, *Acuña 13934* (HAC); Gran Piedra, 27–29 Sept 1959, *Acuña 9914* (HAC); La Gran Piedra, 26 Sept 1959, *Acuña et al. 21122* (HAC); Gran Piedra, Sierra Maestra, 11 Jan 1960, *Alain et al. 7438* (HAC, HAJB); Guamá. Camino entre Pico Joaquín y El Turquino, 1800-1900 msm, 19 Nov 2003, *Bécquer HFC-81644* (HAJB); Santiago de Cuba: falda este de Gran Piedra, 26 Apr 1969, *Bisse & Lippold HFC-14699* (HAJB, JE); Sierra Maestra: Pico cardero, 1000-1300 msm, 13 May 1971, *Bisse & Lippold HFC-19431* (HAJB, JE); Sierra Maestra. El Uvero, pluvisilva de la loma La Francia, 800-1000 msm, 3-5 Feb 1972, *Bisse HFC-21323* (HAJB, JE); IBID *HFC-21335* (HAJB, JE); Sierra Maestra: Pico Cuba, cima, 1800 msm, 12 May 1971, *Bisse & Lippold HFC-18815* (HAJB, JE); Sierra Maestra: subida desde Pico Cardero hasta Pico Cuba, 1300-1800 msm, 10 may 1971, *Bisse & Lippold HFC-19016* (HAJB, JE); IBID *HFC-19091* (HAJB, JE); Sierra Maestra: El Uvero, Loma Siberia, 900-1100 msm, 29 Mar 1969, *Bisse & Lippold HFC-13735* (HAJB, JE); Gran Piedra, loma al sur del cafetal francés, 1000-1100 msm, Jun 1967, *Bisse & Rojas HFC-3794* (HAJB, JE); Sierra Maestra, Loma del Gato, 1 Jan 1948, *Chrysogone NSC-5814* (HAC); Loma del Gato, Cobre Range, Sierra Maestra, 900 m, 1 Jan 1949, *Chrysogone 6374* (HAC, IJ, NY); Sierra de Cobre, Loma del Gato, Hongolosongo, Jan 1933, *Clemente 207* (HAC); Sierra Maestra, Loma del Gato, 1 Jan 1948, *Clemente NSC-5814* (GH); Santiago de Cuba. Entre Motel La Gran Piedra y La Isabelica, 26 Apr 1989, *Dietrich & al. HFC-67420* (B, HAJB, JE); Sierra Maestra, supra Firmeza, 1000 m, 10 Nov 1917, *Ekman 8890* (GH, NY, S); Sierra Maestra, on the water divide between Río Yara and Río Palmamocha, between the last of the picachos and the foot of Loma Joaquín, 1300 m, 19 Jul 1922, *Ekman 14447* (NY, S); Gran Piedra, la Isabelica, 1150 msm, 20 00 20N, 75 37 10W, 15, Nov 2001, *Greuter & al. 25752* (B, HAJB); Gran Piedra, 5 Feb 1971, *Grudzinskaya 759* (HAC); Loma del Gato, Cobre Range of Sierra Maestra, 900–1000 m, 11 Jul-14 Aug 1921, *León LS-10114* (HAC, NY); southern Oriente and Pico Turquino, Sierra Maestra, 1 Jul 1922, *León LS-10920* (HAC, IJ, NY); Cordillera de la Gran Piedra, Sierra Maestra, ca. 1200 m, 18 Mar 1956, *López-Figueiras 2610* (HAC, HAJB, US); Cordillera de la Gran Piedra, Sierra Maestra, ca. 1200 m, 25 Mar 1956, *López-Figueiras 2657* (HAC, HAJB); Alto de la Francia, Uvero, 8 Feb 1971, *Stuchlik 771* (HAC); Sierra Maestra, Alto de la Francia, 8 Feb 1971, *Tinchanitzkaja 757-758* (HAC).

#### 6. Miconia asperifolia

Taxon classificationPlantaeMyrtalesMelastomataceae

(Naudin) Majure & Judd, J. Bot. Res. Inst. Texas. 7: 268. 2013.

Figs 10E–H , 15G–J

Clidemia asperifolia Naudin, Ann. Sci. Nat., Bot. sér. 3, 17: 342. 1852. Type: JAMAICA. “1843–1844, *Purdie s.n.* In insula Jamaica, loco haud indicato. Planta a celeberrimo Hooker communicate”. (lectotype: K![K000812434], designated here; isolectotype: TCD! [TCD0005264]).
Oxymeris asperifolia (Naudin) Triana, Trans. Linn. Soc. London 28: 96. 1871–72. Type. Based on Clidemia asperifolia Naudin
Ossaea asperifolia (Naudin) Triana, Trans. Linn. Soc. London 28: 147. 1871–72. Type. Based on Clidemia asperifolia Naudin
Leandra eggersiana Cogn., Monogr. Phan. [A.DC. & C.DC.] 7: 641. 1891. Type: JAMAICA. Quashi Hill, 5000 ft, 27 Jan 1888, *H.F.A. von Eggers 3759* (lectotype: BR! [BR0000005188772], designated here; isolectotype: BR! [BR0000005188116]).
Ossaea eggersiana (Cogn.) Urb., Repert. Spec. Nov. Regni Veg. 17: 406. 1921. Type. Based on Leandra eggersiana Cogn.

##### Type.

Based on *Clidemia asperifolia* Naudin

##### Description.

Evergreen shrub, 2–5 m tall; stems round in cross section, not ridged, the internodes 0.5–3.5 cm long, stem indumentum of ascending, appressed bulla-based hairs to 0.7 mm long; nodal line present. Leaves opposite, decussate, ovate to elliptic, 1.8–11.6 × 1.05–4.7 cm, slightly anisophyllous, apex acute to acuminate, base acute, cuneate, to slightly rounded, the margins dentate, dentations covered in one large bulla-based hair, venation acrodromous, 5-veined, the midvein and 2 pairs of arching secondary veins, secondary veins mostly basal, the innermost pair suprabasal, produced 1.1–18 mm from leaf base, positioned 2.1–9.8 mm in from margin at widest point of blade, tertiary veins percurrent, more or less perpendicular to midvein, 1.8–5.5 mm apart at midleaf, intertertiary veins occasionally present, tertiary veins often joined by conspicuous, quaternary veins; adaxial leaf surface covered in dorsally compressed, bulla-based hairs, widest hair bases to 1.2 mm, apices of bulla-based hairs mostly recurved towards the leaf margin, young leaf adaxial surface producing long-stemmed, clavate-dentritic hairs along the primary, secondary, and tertiary veins from between the bulla-based hairs, sessile, glandular hairs produced along the primary, secondary, tertiary, and quaternary veins between the bulla-based hairs; abaxial leaf surface covered in bulla-based hairs, these ascending, appressed, those along the primary, secondary, and tertiary veins larger than hairs produced throughout the lamina, the lamina clearly visible, lamina appearing as a series of pits from depressions of the bulla-based hairs produced from the upper leaf surface, sessile, glandular hairs produced throughout the lamina and along veins, domatia of tufts of multicellular hairs produced in the axils of the primary and secondary, primary and tertiary and secondary and tertiary veins; petioles 0.5–3.3 cm long, covered in ascending, appressed, bulla-based hairs on both surfaces. Inflorescences terminal, 19–57 flowered, flowers mostly produced in glomerulate clusters, 2.4–8.2 × 1.6–6.4 cm, the peduncle 0.3–3.2 cm long, proximal inflorescence branches 4–23 mm long; bracts oblong to ovate, 0.6–1.8 mm long; bracteoles narrowly ovate with an attenuate apex, 0.6–0.7 × 0.2–0.3 mm, appearing as enlarged bulla-based hairs, occasionally with smaller bulla-based hairs towards the base of the bracteoles. Flowers 5-merous, sessile or with pedicels to 0.3–0.8 mm long; hypanthium 1.3–2 mm long, short-oblong to globose, 5-lobed, strongly constricted below the torus, free portion of the hypanthium 0.5 mm long, abaxial surface covered in bulla-based hairs to 0.4 mm long, and sessile, glandular hairs between the bulla-based hairs; adaxial surface (i.e., free portion) covered in small, bulla-based hairs; calyx teeth 0.3–1.3 × 0.2–0.5 mm, ascending or spreading, covered in bulla-based hairs; calyx lobes triangular, apex acute, 0.5–1 × 0.7–1.8 mm, covered in bulla-based hairs abaxially and sessile, sparse, glandular hairs adaxially; calyx tube not tearing, 0.4–0.8 mm long with bulla-based hairs abaxially and sessile, glandular hairs adaxially; petals 5, white, elliptic, 5.5–5.7 × 1.3–3.3 mm, with an acute apex and membranous margin, with one to four slightly bulla-based hairs produced abaxially, just below the apex, to 0.7 mm long; stamens 10; filaments 2–2.2 mm long, glabrous, anthers 1.3–2.4 mm long, with one dorsally oriented pore, anther thecae 1.1–2 mm long, anthers with a dorso-basal appendage 0.1–0.2 mm long; style 5.2–5.4 mm long, glabrous, slightly dilated in the middle, collar absent, style subtended by a crown of multicellular, linear to elongate-triangular (needle-like) hairs, which are slightly longer than the surrounding bulla-based hairs of the ovary apex, stigma punctate; ovary 1.8–4.5 × 2.9–5.6 mm, apex flat, pubescent with bulla-based hairs, except for the linear or elongate-triangular hairs forming crown, placentation axile with deeply intruded placenta, 5 locular; berries globose, 5-lobed, purple at maturity, 3–5.5 mm long (including calyx tube), 4.1–5.8 mm wide, seeds 0.5–0.7 mm long, obpyramidal, often falcate, testa smooth, light brown, raphe light brown, smooth, extending the length of the seed.

**Figure 9.**
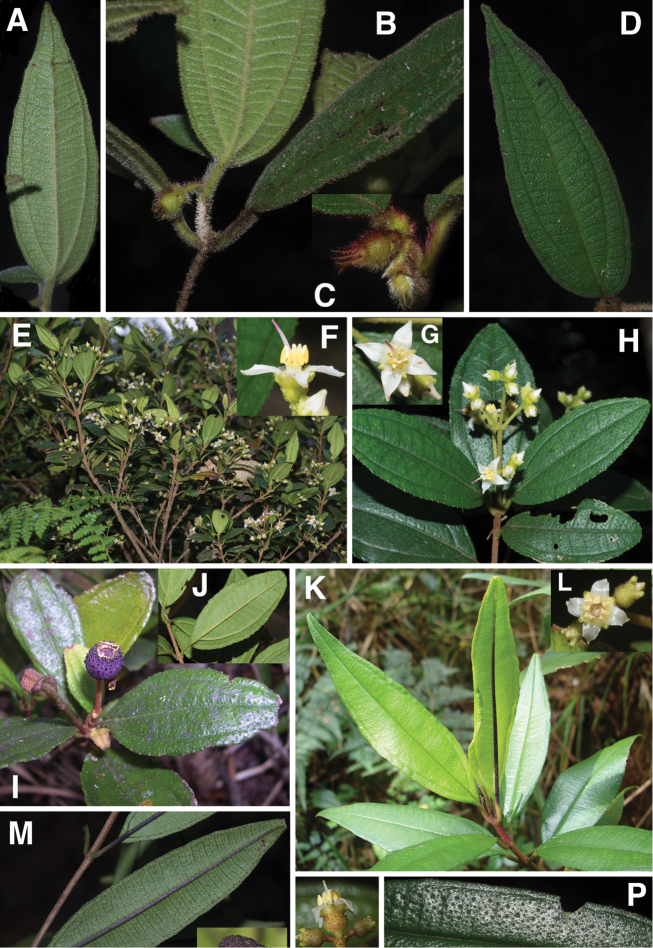
Photographs of *Miconia jashaferi* (**A–D**), *Miconia norlindii* (**E–J**), and *Miconia granulata* (**K–P**). **A** Abaxial leaf surface **B** habit of *Miconia jashaferi* showing pendant infloresence **C** close-up of immature fruit showing long clayx lobes **D** adaxial leaf surface (all from *Michelangeli 2284*) **E** habit of *Miconia norlindii* **F** close-up of flower, side view **G** close-up of flower, frontal view **H** close-up of infloresence (all from *Michelangeli 2213*) **I** mature fruit (*Bécquer s.n.*) **J** leaf abaxial surface (*Michelangeli 2213*) **K** habit of *Miconia granulata* (*Bécquer HFC-82266*) **L** close-up of flower (*Michelangeli 2269*) **M** leaf abaxial surface, showing purple primary vein (*Michelangeli 2265*) **N** mature fruit (*Michelangeli 2269*) **O** close-up of branch of inflorescence (*Michelangeli 2269*) **P** leaf adaxial surface showing reduced bulla-based hairs (*Michelangeli 2265*). Photos **I** & **K** taken by E. Bécquer and **A–H**, **J**, & **L–P** by F. Michelangeli.

**Figure 10.**
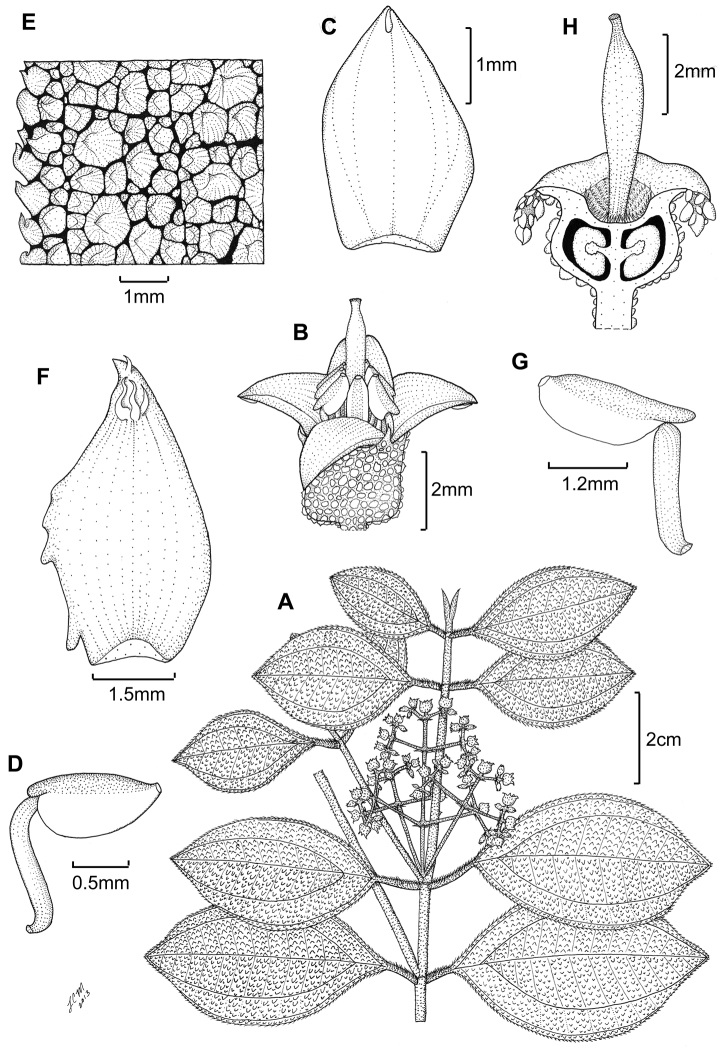
Illustration of *Miconia hybophylla* (**A–D**) and *Miconia asperifolia* (**E–H**). **A** Habit of *Miconia hybophylla* **B** flower **C** petal abaxial surface showing small, slightly bulla-based hair **D** stamen (all from *Ekman H3440* **E**) close-up of adaxial leaf surface of *Miconia asperifolia* (*Judd 5443*) **F** petal abaxial leaf surface showing multiple, slightly bulla-based hairs **G** stamen, and **H** longitudinal section of fruit showing medially expanded style (**G–H** from *Judd 5477*).

##### Phenology.

*Miconia asperifolia* has been collected in flower and fruit from February through August.

##### Distribution

**Figure 11.**
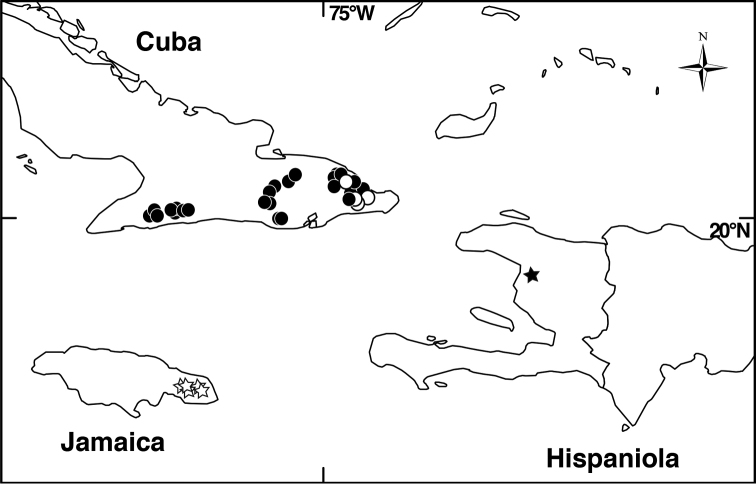
Distribution of *Miconia asperifolia* (open stars), *Miconia hybophylla* (closed star), *Miconia argentimuricata* (closed circles), and *Miconia bullotricha* (open circles).

(Fig. 11). *Miconia asperifolia* is found in the Blue and John Crow Mountains of Jamaica.

##### Ecology.

*Miconia asperifolia* occurs in rainforests or montane, broadleaf cloud forests from 300–1500 m in elevation [see Shreve (1914) for a detailed account of these forests]. Some associated melastomes include *Blakea trinervia* L., *Conostegia montana* D.Don, *Miconia dodecandra* (Desr.) Cogn., *Micona quadrangularis* Naudin, *Miconia laevigata* (L.) DC., *Miconia tetrandra* (Sw.) D.Don ex G.Don, and *Mecranium virgatum* (Sw.) Triana.

##### Conservation status.

*Miconia asperifolia* is a widespread species, which also occurs in the protected Blue Mountains National Park, so the species should be considered stable.

##### Discussion.

**Figure 12.**
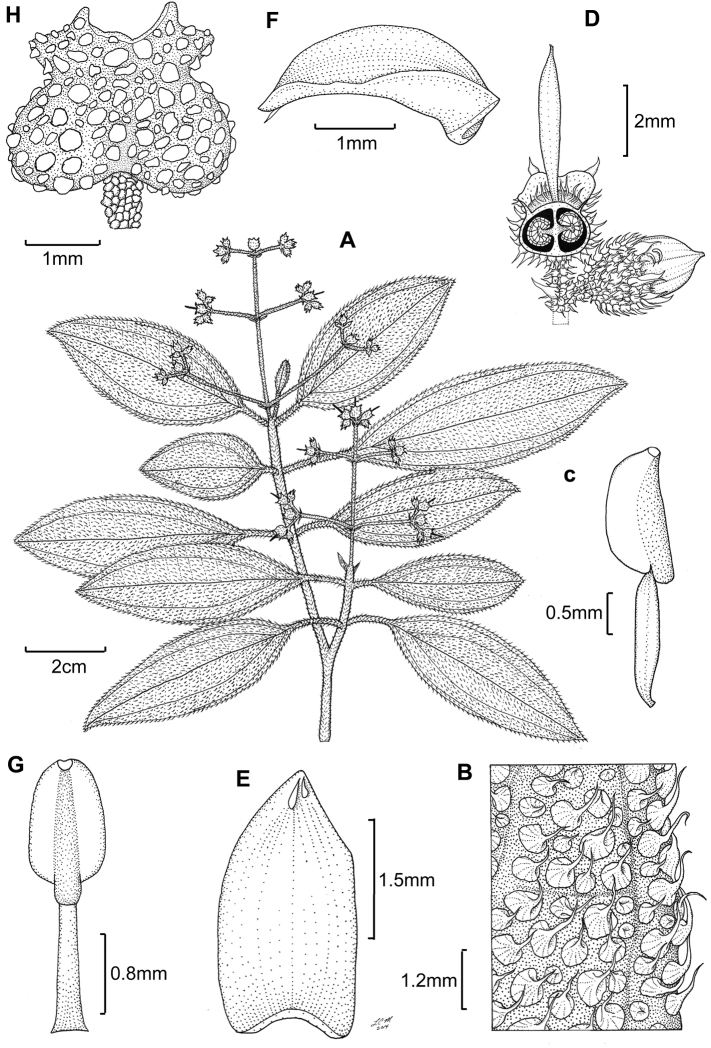
Illustration of *Miconia cubana* (**A–D**) and *Miconia ottoschmidtii* (**E–H**). **A** Habit of Miconia cubana **B** close-up of leaf adaxial surface showing bulla-based hairs not entirely covering areoles **C** stamen **D** fruit longitudinal section and flower bud (all from type *Wright 189*) **E** petal abaxial surface of *Miconia ottoschmidtii* showing small, slightly bulla-based hairs **F** petal side view **G** stamen showing dorso-basal appendage (**E–G** from *Ekman 6926*) **H** fruit showing granulate bulla-based hairs on the hypanthium (*León LS-10046*).

*Miconia asperifolia* is resolved as sister to *Miconia granulata* in a subclade consisting of Cuban species. However based on morphological similarity, *Miconia asperifolia* appears likely to be sister to *Miconia hybophylla*, a rare Haitian species not included in our phylogenetic analysis (see below under discussion about *Miconia hybophylla*). Both species have similar adaxial leaf surfaces with well-formed by slightly compressed bulla-based hairs composed of larger hairs generally surrounded by smaller hairs. Both species also have numerous well-formed domatia in the axils of the primary and secondary, primary and tertiary, and secondary and tertiary veins, as well as large, expanded, cymose inflorescences (Figs 10, 15G–J). Populations of *Miconia asperifolia* differ slightly between the John Crow and Blue Mountains with those in the John Crow Mountains tending to have larger leaves and less well developed bulla-based hairs on the upper leaf surface, however, these characters form a gradient from one geographic area to the next and represent only minor intraspecific variation. Shade forms of *Miconia asperifolia* often have poorly formed hairs on the upper leaf surface (Fig. 15I), which resemble a putatively closely related Cuban species, *Miconia cubana* (Fig. 12).

William Purdie was assigned by J.D. Hooker to collect plants in Jamaica during the years 1843–1844 (Sinha 1972). Naudin (1852) states that his new species, *Clidemia asperifolia*, was brought to his attention by Hooker based on material sent from Jamaica (“In insula Jamaica, loco haud indicato. Planta a celeberrimo Hooker communicate.”). Thus, we conclude that the specimens collected by Purdie are the type material and have been used as the lectotype (see above).

##### Specimens examined.

**JAMAICA. Portland Parish**: Trail from Morce’s Gap north toward Vinegar Hill, Elev. 4700–5000 ft, 19 Aug 1966, *Anderson & Sternberg 3478* (US, MICH, MO); southeastern foothills of the John Crow Mts., 2 Mar 1909, *Harris 10682* (NY, F); John Crow Mts., 11 Mar 1909, *Harris 10773* (NY, US, F); John Crow Mts., ca. 2 mi SW of Ecclesdown following forestry road W and then trail into slopes of mts., 18.041961°N, -76.352628°W, 21 Jan 2011, *Ionta 2005* (FLAS, IJ); Blue Mountians. Green Hills, just N of Hardwar Gap, 1150 m, 23 May 1987, *Judd 5443* (F, FLAS, IJ, MO, NY); ca. 5 mi SW of Priestmans River, ca. 1500 ft, 6 Feb 1953, *Proctor 7620* (IJ, US); northwest slope of Joe Hill, 1000–2250 ft, 20 Apr 1955, *Proctor 10090* (FLAS, HAC, IJ, NY, US); upper N slope of Silver Hill Gap, along track toward Big Level, ca. 3600’, 24 Feb 1963, *Proctor 23256* (IJ, MICH); Moore Town, 500 ft, 10 Aug 1965, *Proctor 26600* (IJ); gorge of the Stony River below jct of the Macunga River, 1200 ft, 23 Jul 1967, *Proctor 28226* (IJ). **St. Andrew Parish**: Vicinity of Moody’s Gap, 10 Sep 1908, *Britton 3397* (NY); Silver Gap, 3500 ft, 15 May 1896, *Harris 6292* (NY, US, F); Just below Hardwar Gap on rd. to Newcastle, 1160 m, 17 May 1987, *Judd 5359* (FLAS, F, IJ, JBSD, MO, S); Blue Mountains. Catherines Peak (along road from Newcastle to radio towers at peak itself), 1150–1500 m, 25 May 1987, *Judd 5477* (FLAS, IJ); Blue Mountains, 1.3 to 2 mi N of Newcastle on rd. to Hollywell and Hardwar Gap, from 18°4.68'N -76°43.156'W to 18°4.955'N -76°43.531'W, 1200–1231 m, 11 Jan 2011, *Judd 8300* (FLAS, IJ); 0.5 mi N of Hardwar Gap, near the waterfall, 3900 ft, 29 Apr 1956, *Proctor 10163* (HAC, IJ, NY, US); West slope of Mt. Horeb above Hardwar Gap, 4200 ft, 11 May 1955, *Proctor 10192* (IJ, NY); Blue Mountains, along road from Newcastle to Catherine’s Peak, 1330 m, 15 Jul 1986, *Skean 1869* (FLAS, IJ, NY); Blue Mountains, along road from Newcastle to Catherine’s Peak, 15 Jul 1986, *Skean 1869* (FLAS, IJ, NY, US); along the trail to Mt. Horeb originating where Woodcutter’s Gap trail intersects the paved road to Catherine’s Peak, 1430 m, 15 Jul 1986, *Skean 1877* (FLAS, IJ, NY). **St. Thomas Parish**: Corn Puss Gap, N slope, 2000 ft, 6 Jan 1945, *Barry s.n.* (IJ); Corn Puss Gap, 2100 ft, 25 Apr 1946, *Barry s.n.* (IJ); Slopes, Cuna Cuna Gap, 1–13 Mar 1909, *Britton 4046* (US, NY); ridge above highest point of Cuna-Cuna Pass, leading towards Lookout Bump, 17 deg 60'N, 76 deg 23'W, 800–900 m, 19 Jan 2004, *Christenhusz & Tuomisto 3132* (IJ); eastern end of the Blue Mountains (near jct. with John Crow Mts.) NE of Hayfield, along Cuna Cuna trail, 17°59'24.7"N, -76°22'52.7"W, 22 Jan 2011, *Judd 8337* (FLAS, IJ); along the Wild Cane River 1.1 miles due southeast of Macungo Hill, 1500 ft, 14 May 1968, *Proctor 28711* (FLAS, IJ). **Portland/St. Thomas Parish**: Quashi Hill, 5000 ft, 27 Jan 1888, *Eggers 3757* (BR).

#### 7. Miconia hybophylla

Taxon classificationPlantaeMyrtalesMelastomataceae

(Urb.) Majure & Judd, J. Bot. Res. Inst. Texas. 7: 268. 2013.

Fig. 10A–D

Ossaea hybophylla Urb., Ark. Bot. 21A(5): 51. 1927. Type: HAITI. Massif des Cahos, Petite-Riviére de l’Artibonite, Pérodin, at Ingram, 7 Mar 1925, *E.L. Ekman H3440* (lectotype: S! [S-R-10017], designated here; isolectotypes EHH n.v., G! [G00353948], NY! [NY00099692], US! [US00123686], K! [K000535605]).
Leandra hybophylla (Urb.) Alain, Sida 18: 1026. 1999. Type. Based on Ossaea hybophylla Urb.

##### Type.

Based on *Ossaea hybophylla* Urb.

##### Description.

Small evergreen tree (height unknown); young stems purplish, round to slightly quadrangular with rounded angles in cross section, not ridged, the internodes 0.6–4.3 cm long, stem indumentum of granulate, bulla-based hairs to 0.3 mm long, these spreading; nodal line present but inconspicuous. Leaves opposite, decussate, broadly elliptic, 2.2–4.5 × 1.6–2.6 cm, slightly anisophyllous, margins with conspicuous spine-tipped hairs, these spreading (especially at base of leaf) to appressed or recurved along the leaf margin, apex acute, base acute often asymmetrical, venation acrodromous, 5-veined, the midvein and 2 pairs of arching secondary veins, the outermost pair of secondary veins ocasionally intramarginal, mostly basal, the innermost pair, suprabasal, asymmetrical or symmetrical, produced 2.5–6.5 mm from leaf base, positioned 2.2–4.3 mm in from margin at widest point of blade, tertiary veins percurrent, more or less perpendicular to midvein, 1.8–3.3 mm apart at midleaf, intertertiary veins rarely present and inconspicuous when present, tertiary veins often joined by quaternary veins; adaxial leaf surface covered in bulla-based hairs, widest hair bases to 0.7 mm, apices of bulla-based hairs mostly erect or recurved towards the leaf margin, young leaf adaxial surface producing long-stemmed, clavate-dentritic hairs along the primary, secondary, and tertiary veins from between the bulla-based hairs, sessile, glandular hairs produced along the primary, secondary, tertiary, and quaternary veins between the bulla-based hairs, especially toward the base of the leaf; abaxial leaf surface with sparse bulla-based hairs, these mostly erect to spreading, those along the primary, secondary, and tertiary veins larger than hairs produced throughout the lamina, the lamina clearly visible, olive green or occasionally purplish, not deeply pitted, sessile, glandular hairs produced throughout the lamina, conspicuous domatia produced as tufts of hairs at the junction of the primary and secondary veins, as well as at the junctions of the primary and secondary veins with the tertiary veins; petioles 0.5–1.1 cm long, purplish, adaxial surface of petiole with spreading, multicellular hairs to 1.2 mm long, the rest of the petiole covered in short 0.1–0.4 mm spreading, bulla-based hairs. Inflorescences terminal, pyramidal, purplish, with up to 41 flowers, flowers produced in cymose clusters, 1.7–4 × 2–3.1 cm, the peduncle 0.1–1.4 cm long, proximal inflorescence branches 6–13 mm long, bracts oblong to narrowly ovate, 0.6–1.5 mm long; bracteoles narrowly ovate, 0.4–0.8 × 0.2–0.25 mm, appearing as large bulla-based hairs. Flowers 4-merous, pedicels 0.5–0.8 mm long; hypanthium 1.3–2.7 mm long, short-oblong to globose, 4-lobed, slightly constricted below the torus; free portion of the hypanthium ca. 0.5 mm long, abaxial surface covered in dorsi-ventrally compressed or erect bulla-based hairs to 0.1 mm long, and sessile, glandular hairs between the bulla-based hairs, adaxial surface (i.e., free portion) covered in small, bulla-based hairs; calyx teeth 0.5–1 × 0.3–0.6 mm, spreading with straight or recurved apices, having the general appearance of a large bulla-based hair and often with a large bulla-based hair at the base; calyx lobes triangular, apex acute, 0.9–1.2 × 1.4–1.7 mm, covered in bulla-based and sessile, glandular hairs abaxially and sessile, glandular hairs adaxially; calyx tube not tearing, 0.3–0.5 mm long with bulla-based hairs abaxially and sessile, glandular hairs adaxially, clavate-dendritic hairs produced at the apex of the calyx tube; petals 4, most likely white, 2–3 × 1.7–1.8 mm, ovate, apex acute, with one slightly bulla-based hair produced just below the apex on the abaxial surface, to 0.3 mm long; stamens 8; filaments 1.2–1.3 mm long, glabrous, anthers 1.1–1.3 mm long, with one dorsally oriented pore, anther thecae 0.9–1.1 mm long, anthers with a dorso-basal appendage 0.2 mm long; style 3.5–4.3 mm long, glabrous, not or only slightly dilated in the middle (mostly oblong), collar absent, style subtended by an inconspicuous crown of triangular hairs (longer than those on the rest of the ovary apex), which are slightly longer than the surrounding bulla-based hairs of the ovary apex, stigma punctate; ovary 1.3–4 × 1.9–2.4 mm, 4-lobed, apex truncate, pubescent with triangular, bulla-based hairs, placentation axile with deeply intruded placenta, 4-locular; immature berries globose, 4-lobed, color at maturity unknown, but probably more or less purple, 2.5–2.7 mm long (including calyx tube), 3.1– 3.3 mm wide, seeds 0.4–0.5 mm long, obpyramidal, testa smooth, raphe smooth, extending the length of the seed.

##### Phenology.

Flowers at anthesis, as well as buds and immature fruit were present on the type collection, which was gathered in March.

##### Distribution.

(Fig. 11). Haiti, Massif du Cahos, Petite-Riviére de l´Artibonite, Pérodin, at Ingram; known only from the type collection.

##### Ecology.

Nothing is known regarding the ecology of this species.

##### Conservation status.

Insufficient data are available for determining the conservation status of this species, although *Miconia hybophylla* is very likely endangered as a result of forest clearing for subsistence agriculture and charcoal production in west-central Haiti.

##### Discussion.

*Miconia hybophylla* is likely sister to *Miconia asperifolia* and can be easily recognized as a smaller, more compact version of *Miconia asperifolia*, as compared with other species in the *Lima* clade. The two species also share very, well developed domatia in the axils of the primary and secondary veins, as well as the axils of the tertiary with primary and tertiary with secondary veins.

##### Specimens examined.

This species is only known from the type specimen.

#### 8. Miconia granulata

Taxon classificationPlantaeMyrtalesMelastomataceae

(Urb.) Majure & Judd, J. Bot. Res. Inst. Texas. 7: 268. 2013.

Figs 7A–E , 9K–P

Ossaea granulata Urb., Symb. Antill. (Urban) 9(1): 125. 1923. Type: CUBA. Provincia Oriente [Guantánamo], Baracoa at Minas de Yberia (pr. Taco Bay) in “charrascales,” 800 m, 7–8 Dec 1914, *E.L. Ekman 3789* (lectotype: S! [S05-3777], designated here; isolectotype: NY! [NY00099690]).

##### Type.

Based on *Ossaea granulata* Urb.

##### Description.

Evergreen shrub, to 2 m tall; young stems round in cross section, purplish, not ridged, the internodes 0.7–5.5 cm long, stem indumentum of granulate hairs (dorsi-ventrally compressed bulla-based hairs) to 0.2 mm long, these spreading; nodal line present but inconspicuous, composed of larger granulate hairs than those of the internodes. Leaves opposite, decussate, narrowly ovate to narrowly elliptic, 2.5–9.4 × 1–2.8 cm, slightly to moderately anisophyllous, apex long acute to slightly acuminate, base acute, venation acrodromous, 3-veined, the midvein and 1 pair of arching secondary veins, secondary veins only occasionally intramarginal, suprabasal or basal, produced 0.5–5.1 mm from leaf base, positioned 0.5–2.7 mm in from margin at widest point of blade, tertiary veins percurrent, more or less perpendicular to midvein, 1.1–2.8 mm apart at midleaf, intertertiary veins present, tertiary veins only rarely joined by quaternary veins; adaxial leaf surface covered in bulla-based hairs, these dorsi-ventrally compressed, widest hair bases to 0.8 mm, young leaf adaxial surface producing long-stemmed, clavate-dentritic hairs along the primary, secondary, and tertiary veins from between the bulla-based hairs, sessile, glandular hairs absent; abaxial leaf surface covered in sparse, bulla-based hairs, these strongly dorsi-ventrally compressed and inconspicuous, those along the primary, secondary, and tertiary veins larger than hairs produced throughout the lamina, the lamina clearly visible, with a series of pits resulting from depressions of the bulla-based hairs produced from the upper leaf surface, sessile, glandular hairs absent; petioles 0.3–1.3 cm long, covered in granulate, bulla-based hairs on both surfaces. Inflorescences terminal, 12–>30 flowered, flowers produced in cymose clusters, 1.2–2.7 × 2.5–3 cm, the peduncle 0.1–1.9 cm long, proximal inflorescence branches 4–10.1 mm long; bracts oblong to ovate, 0.4–1.3 mm long; bracteoles ovate, 0.4–0.5 × 0.2–0.4 mm, covered in granulate bulla-based hairs. Flowers 4-merous, with pedicels to 0.1–1 mm long; hypanthium 1.8–2.3 mm long, globose, strongly 4-lobed, constricted below the torus, free portion of the hypanthium 0.5–0.6 mm long, abaxial surface covered in granulate bulla-based hairs to 0.1 mm long, and sessile, glandular hairs near the bases of the bulla-based hairs; adaxial surface (i.e., free portion) covered in granulate, bulla-based hairs; calyx teeth 0.5–1.4 × 0.3–0.6 mm, ascending or spreading, covered in bulla-based hairs; calyx lobes more or less triangular, apices acute, 0.2–0.6 × 1.2–1.5 mm, covered in bulla-based hairs abaxially and sessile, sparse, glandular hairs adaxially; calyx tube not tearing, 0.3–0.4 mm long with bulla-based hairs abaxially and sessile, glandular hairs adaxially; petals white, narrowly ovate, 2.5–3.2 × 1.3–1.5 mm, apex acute, with one slightly bulla-based hair produced abaxially, just below the apex, to 0.2 mm long; stamens 8; filaments 1.1–1.2 mm long, glabrous, anthers 1.25–1.3 mm long, yellow, with one dorsally oriented pore, anther thecae 1.1–1.2 mm long, anthers with a dorso-basal appendage 0.1–0.15 mm long; style 3.1–3.2 mm long, glabrous, notably or only slight dilated just below the apex, collar absent, crown of very short, bulla-based hairs slighty longer than surrounding hairs of the ovary, stigma punctate; ovary 0.9–1.4 × 1.7–2.4 mm, apex with bulla-based hairs, except for the linear or elongate-triangular hairs forming crown, placentation axile with deeply intruded placenta, 4-locular; berries globose, 4-lobed, purple-black at maturity, ca. 4 mm long (including calyx tube), 4 mm wide, seeds 0.5 mm long, obpyramidal, often falcate, testa smooth, light brown, raphe dark brown, smooth, extending the length of the seed.

##### Phenology.

This species has been collected in bud in August, in flower and mature fruit in November, immature fruit in December, as well as mature fruit in April.

##### Distribution

(Fig. 8). *Miconia granulata* is restricted to the northern portion of eastern Cuba in the Guantánamo and Holguín provinces in the areas with moderate elevation, from Baracoa to Moa.

##### Ecology.

*Miconia granulata* occurs in “charrascales”, more or less thorny, xerophytic scrub and semidry montane rainforest on serpentine soils, from 100 to roughly 800 m in elevation. Associated melastome species are *Miconia walterjuddii*, *Ossaea rufescens* (Griseb.) C.Wright, *Meriania angustifolia* (Cogn.) Carmenate & Michelangeli and *Calycogonium bissei* Bécquer.

##### Conservation status.

Bécquer (2007) considered *Miconia granulata* to be threatened, as it is known from relatively few locations and only occurs in one protected area, Parque Nacional Alejandro de Humboldt. We propose a preliminary conservation status of Critically Endangered owed to ongoing and future mining practices in the zone where it occurs, as well as the fact that *Miconia granulata* is not an abundant species in any part of its range.

##### Discussion.

Based on phylogenetic analyses, *Miconia granulata* is resolved in a clade wtih *Miconia argentimuricata* and *Miconia asperifolia* and is sister to *Miconia asperifolia* (Fig. 2), a Jamaican species. It is the only member of this clade with 4-merous flowers and occasionally pendulous inflorescences (Figs 7B, 9K–P), however, those characters are also shared with *Miconia bullotricha*, which may be closely related to *Miconia granulata* (see below under *Miconia bullotricha*). *Miconia granulata* also has very poorly developed bulla-based hairs on the upper leaf surface, as compared to its closest relatives (Fig. 9 K, P).

##### Specimens examined.

**CUBA: Guantánamo.** Alto entre loma del Mirador y loma de Buena Vista, 500 msm, 6 Aug 1975, *Álvarez & al. HFC-27126* (B, HAJB, JE); IBID *HFC-27130* (HAJB); falda suroeste de la Loma del Mirador, 500 msm, 9 Aug 1975, *Álvarez & al. HFC-27212* (B, HAC, HAJB, JE); Baracoa, Río Báez hacía el campamento los Naranjos, 21 Jan 1977, *Álvarez et al. HFC-33783* (HAC); Aserrío Nuevo Mundo, pluvisilva de montaña 2-4 km al sur del aserrío, 400 msm, Apr 1975, *Areces & al. HFC-25759* (HAJB); Baracoa, loma Los Guineos, 400-500 msm, 12 apr 1986, *Arias & al. HFC-58603* (B, JE); Baracoa, loma Los Guineos, 14 Apr 1986, *Arias & al. HFC-58639* (B, HAJB, JE); IBID *HFC-58700* (B, HAJB, JE); Baracoa: cerca del aserrío Nuevo Mundo, 28 Aug 1971, *Bisse HFC-19585* (HAJB, JE); Baracoa: Pluvisilva al sur de la loma del Yunque, 300-400 msm, 9-10 Feb 1972, *Bisse HFC-21469* (HAJB, JE); Baracoa: valle al noroeste del Yunque de Baracoa, Feb 1968, *Bisse & Köhler HFC-5211* (HAJB, JE); Baracoa: subida a la Mina Iberia, 300-700 msm, Mar 1968, *Bisse & Köhler HFC-6162* (HAJB, JE); Baracoa: pluviosilva al sur de la Loma del Yunque, 300-400 msm, Jun 1967, *Bisse & Rojas HFC-2725* (HAJB, JE); Baracoa. Camino de Los Naranjos a la Loma de Buenavista, 21 Jan 1977, *Bisse & al HFC-33783* (B, HAC, HAJB, JE); **Holguín.** Moa, Monte La Breña, Oriente, 5 Nov 1945, *Acuña 13288* (HAC, HAJB, NY); Moa. Pluviosilva Km 8-10 del camino de La Melba, 100 msm, 1 May 1980, *Álvarez & al. HFC-42557* (B, HAJB, JE); Moa. Piloto, 6 May 1973, *Álvarez & Berazaín HFC-24386* (HAJB); Moa, camino a La Melba, entre el km 10 y el arroyo Las Comadres, 27 Apr 2004, *Bécquer HFC-82266* (FLAS, HAJB, NY); Moa. En el camino del aserrío La Melba, 20 Jan 1988, *Berazaín & al. HFC-63314* (HAJB); Moa: Cayo Probado, orillas de las cabezas del Río Jiguani, 3 Apr 1972, *Bisse & Berazaín HFC-21871* (HAJB, JE); Moa, charrascales en el altiplano de la Sierra de Moa, Mar 1968, *Bisse & Köhler HFC-6739* (HAJB, JE); Moa: La Melba, falda este de la Sierra de Moa, 800-1000 msm, 23 Dec 1968, *Bisse & Lippold HFC-11454* (HAJB, JE); Moa: La Melba, pluviosilva de montaña cerca del aserrío, 500 msm, 22 Dec 1968, *Bisse & Lippold HFC-11630* (HAJB, JE); Moa: La Melba, charrascal cerca del aserrío, 400-500 msm, 22 Dec 1968, *Bisse & Lippold HFC-11640* (HAJB, JE); Moa: charrascales en el altiplano de la Sierra de Moa, 600-900 msm, 7 Jan 1969, *Bisse & Lippold HFC-11903* (HAJB, JE); La Melba, charrascal cerca del aserrío, 400-500 msm, Jun 1967, *Bisse & Rojas HFC-3242* (HAJB, JE); Alto de la Iberia, 700 m, 23 Mar 1970, *Borhidi et al. 121/8* (HAC); Monte Breña, Moa, Aug 1945, *Clemente et al. 4705* (HAC); La Breña Woods, Moa region, Oriente, 1 Aug 1945, *León LS-22577* (HAC, IJ, NY); La Breña woods, Moa, 1 Aug 1945, *León LS-22594* (GH, HAC, NY); Moa, Parque Nacional Alejandro de Humboldt, carretera a La Melba, km 28, 14 Nov 2013, *Michelangeli et al. 2265* (NY); Moa, Parque Nacional Alejandro de Humboldt, carretera a La Melba, km 16–18, 15 Nov 2013, *Michelangeli et al. 2269* (NY); Moa-Baracoa, Nov 1965, *Yeno 1088* (HAC).

#### 9. Miconia cubana

Taxon classificationPlantaeMyrtalesMelastomataceae

(Alain) Majure & Judd, J. Bot. Res. Inst. Texas. 7: 268. 2013.

Fig. 12A–D

Ossaea cubana Alain, Contr. Ocas. Mus. Hist. Nat. Col. “de la Salle” 14: 11. 1955. Type: CUBA. [Pinar del Río], Isabel María, 16 Mar, 1860–1864, *C. Wright 189* (holotype: NY! [NY00099639]; isotypes: HAC (4 sheets)!, GH! [GH00713104, branch in upper left hand corner], S (2 sheets)! [S12-26503, S12-26504]).

##### Type.

Based on *Ossaea cubana* Alain

##### Description.

Evergreen shrub (height unknown); stems round in cross section, not ridged, the internodes 0.6–7 cm long, stem indumentum of previous season’s growth bright white, contrasting with purplish hairs of current season’s growth, as well as purplish petioles, indumentum of bulla-based hairs, 0.2–1 mm long, these spreading to ascending with the apices recurved towards the stem axis; nodal line absent. Leaves opposite, decussate, oblong to elliptic, 1.5–9.1 × 0.8–3.3 cm, slightly to moderately anisophyllous, apex broadly or narrolwly acute, base acute, venation acrodromous, 5-veined, the midvein and 2 pairs of arching secondary veins, outermost pair of secondary veins mostly basal, the innermost pair, suprabasal, produced 2.1–9 mm from leaf base, positioned 3.8–6.6 mm in from margin at widest point of blade, tertiary veins percurrent, more or less perpendicular to midvein, 2.8–5.2 mm apart at midleaf, intertertiary veins absent, tertiary veins often joined by quaternary veins; adaxial leaf surface with sparse, bulla-based hairs, the lamina clearly visible, widest hair bases to 0.7 mm, apices of bulla-based hairs mostly recurved, young leaf adaxial surface producing sessile, glandular hairs produced along the primary, secondary, tertiary, and quaternary veins between the bulla-based hairs, especially toward the base of the leaf; abaxial leaf surface covered in sparse narrow, bulla-based hairs, these to 0.1 mm wide, those along the primary, secondary, and tertiary veins larger than hairs produced throughout the lamina, the lamina clearly visible, appearing as a series of pits from depressions of the bulla-based hairs produced from the upper leaf surface, sessile, glandular hairs produced throughout the lamina, domatia absent; petioles 0.7–2.8 cm long, purplish, covered in spreading to ascending, bulla-based hairs on both surfaces. Inflorescences terminal, expanded cymes, purplish, 19–32 flowered, 3.7–4.8 × 3.7–4.4 cm, the peduncle 0.15–0.9 cm long, proximal inflorescence branches 8–25 mm long, bracts oblong to narrowly ovate, 0.8–1.1 mm long; bracteoles narrowly ovate, 0.5–0.6 × 0.15–0.3 mm, covered in bulla-based hairs. Flowers 4-merous, pedicels 0.4–1.1 mm long; hypanthium 1.5–1.8 mm long, globose, 4-lobed, slightly constricted below the torus, free portion of the hypanthium 0.2–0.25 mm long, abaxial surface covered in bulla-based hairs 0.4–1 mm long, and abundant, sessile, glandular hairs near the bases of the bulla-based hairs; adaxial surface (i.e., free portion) covered in small, bulla-based hairs and sessile, glandular hairs; calyx teeth 0.6–1.1 × 0.15–0.2 mm, spreading with the apices recurved upwards, essentially appearing as a large bulla-based hair; calyx lobes 0.9–1 × 1.3–1.8 mm, rounded apically, covered in bulla-based hairs abaxially and sessile, glandular hairs adaxially; calyx tube not tearing, 0.7–0.8 mm long with bulla-based hairs abaxially and sessile, glandular hairs adaxially; petals 4, white (?), ovate with acute to acuminate apices, 2 mm long (according to Alain 1955), with several slightly bulla-based hairs produced abaxially, these sometimes bent in the middle or at the base, 2–3 of these hairs produced at the center of the petal and 1 hair produced just below the apex, hairs to 0.7 mm long; stamens 8; filaments 1.5–1.6 mm long, glabrous, anthers 1.3–1.5 mm long, with one dorsally oriented pore, anther thecae 1.2–1.3 mm long, anthers with a dorso–basal appendage 0.1–0.2 mm long; style 4.2–4.4 mm long, glabrous, dilated in the middle, collar absent, style not subtended by a crown, stigma punctate; ovary 1.6–1.8 × 1.9–2 mm, apex rounded, with bulla-based hairs, placentation axil with deeply intruded placenta, 4-locular; immature berries globose, slightly 4-lobed, color at maturity unknown, but likely purple, 1.6–1.7 mm long (including calyx tube), 1.8–2.2 mm wide, immature seeds obpyramidal, testa smooth, with dark raphe extending the entire length.

##### Phenology.

*Miconia cubana* was collected in bud, flower and immature fruit on March 16, if our interpretation of the label data is correct. However, no petals were seen on the type specimens (other than in bud), where only styles and a couple of stamens were present.

##### Distribution

**Figure 13.**
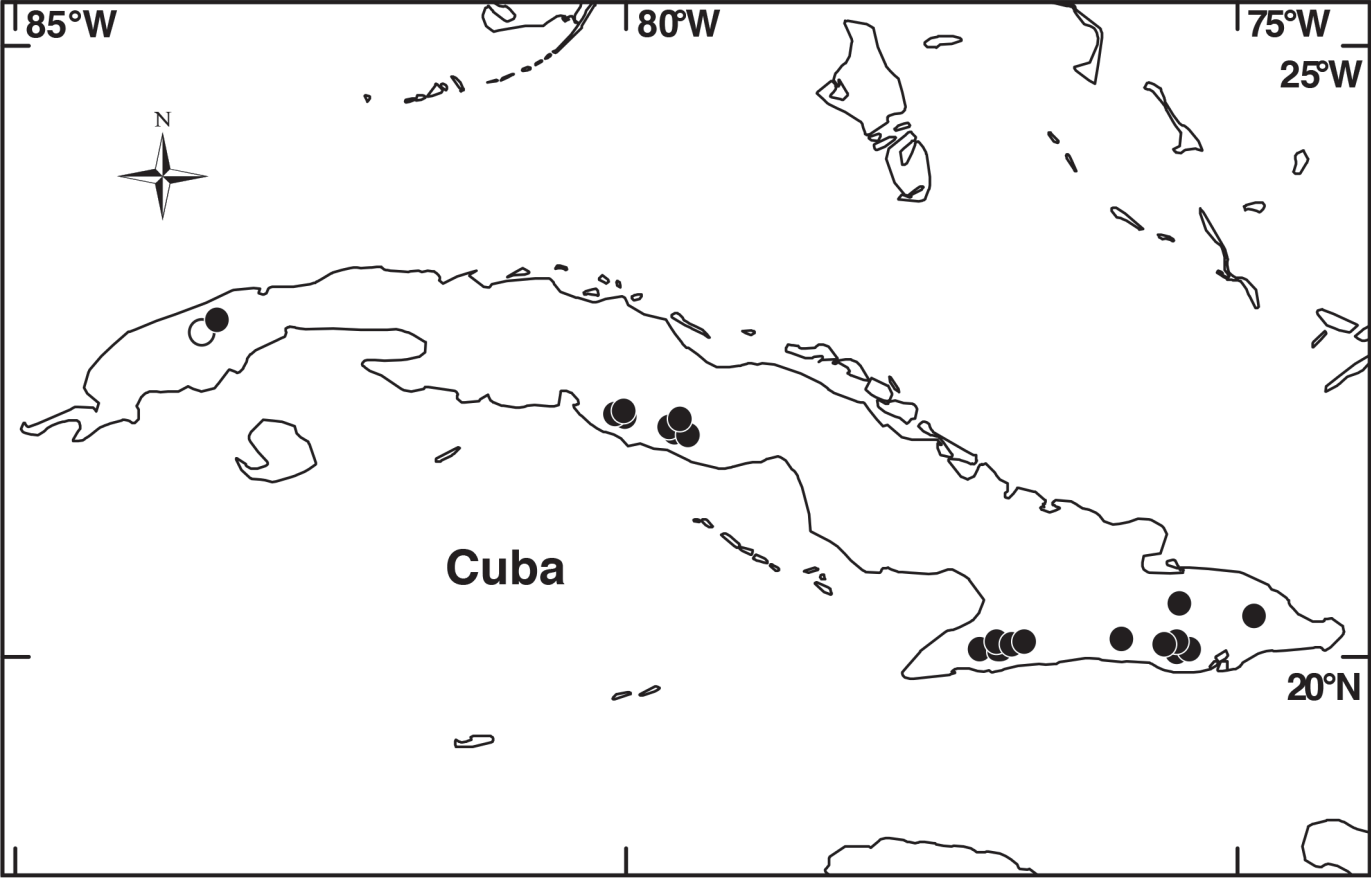
Distribution of *Miconia cubana* (open circle) and *Miconia ottoschmidtii* (close circles).

(Fig. 13). *Miconia cubana* may be restricted to Pinar del Río, Cuba, if our interpretation of Wright’s collections is correct. Three separate labels were distributed by A. Gray of the type material collected by Wright, which at the time was considered to be another species, *Miconia asperifolia* (see Alain 1955). The sheet of the isotype of *Miconia cubana* at GH (GH00713104) actually is composed of branches from two species, *Miconia cubana* and *Miconia norlindii*. *Miconia cubana* is the branch in the upper left hand corner on that sheet. It is presumed that the two other labels associated with that specimen actually pertain to *Miconia norlindii*, as they both refer to eastern Cuba (i.e., Loma del Gato and Cuba Orientali), where *Miconia norlindii* is found. The third label contains the inscription, Isabel María, which may refer to a valley in western Cuba (Pinar del Río); Wright collected for a time in western Cuba (provinces of La Habana and Pinar del Río) during 1863 and 1864, including trips to Elemento Natural Destacado (END) Pan de Guajaibón in Pinar del Río (Underwood 1905) just north of what is now Reserva Ecológica Sierra la Guira and Retiro. During March of 1860–1862, Wright was in southern Cuba (Monte Verde, Sagua de Tánamo), as well as west-central Cuba (Ciénaga) but apparently not in the Sierra Maestra around Pico Turquino (Underwood 1905). So the specimens of *Miconia norlindii* associated with the type of *Miconia cubana* must have been collected at a different geographical locality and date from the material of *Miconia cubana*. Therefore, we interpret the Isabel María locality to actually pertain to *Miconia cubana*, contrary to Howard and Wright (1988) who placed the species around Pico Turquino. Duplicates of *Wright 189* at P (P052311330 and BR (BR0000013239626) are specimens of *Miconia norlindii*, not *Miconia cubana*, likely a result of Gray’s mixing of Wright’s labels and specimens, as described above.

##### Ecology.

Nothing is known regarding the ecology of *Miconia cubana*.

##### Conservation status.

Data are insufficient for determing the conservation status of *Miconia cubana*.

##### Discussion.

*Miconia cubana* may be closely related to *Miconia asperifolia* considering the large, expanded, cymose inflorescences and the reduced bulla-based hairs (not filling the areoles) that are seen in some specimens of *Miconia asperifolia*.

##### Specimens examined.

*Miconia cubana* is only known from the type gathering by Charles Wright.

#### 10. Miconia argentimuricata

Taxon classificationPlantaeMyrtalesMelastomataceae

Majure & Judd, J. Bot. Res. Inst. Texas. 7: 268. 2013.

Figs 14 , 15A–F

Calycogonium muricatum Griseb., Cat. Pl. Cub. 95. 1866. Type: CUBA. Guantánamo, Cuchillas de Baracoa, 14 May, 1860–1864, *C. Wright 2485* (lectotype: GOET! [GOET007038], designated here; isolectotypes: GDC! [G00328229], GH! [GH00073119], GOET! (as *C. Wright 682*), HAC!, K! [K000329532], MO! [MO-2049514], YU! [YU065051]).
Ossaea muricata (Griseb.) C. Wright, Anal. Acad. Ci. Habana 5: 434. 1868. Type. Based on Calycogonium muricatum Griseb.

##### Type.

Based on *Calycogonium muricatum* Griseb.

##### Description.

Evergreen shrub, to 1.5 m tall; stems round in cross section, not ridged, the internodes 0.5–10.9 cm long, stem indumentum of ascending, bulla–based hairs to 2.2 mm long; nodal line absent. Leaves opposite, decussate, broadly to narrowly elliptic, 2.7–9.3 × 1.4–4.5 cm, slightly anisophyllous, apex acute to acuminate, base rounded to acute, margins dentate, dentations covered in well-developed bulla-based hair, venation acrodromous, 5–7-veined, the midvein and 3 pairs of arching secondary veins, the outermost intramarginal, secondary veins mostly basal to suprabasal, the innermost pair suprabasal, produced 2.5–17 mm from leaf base, positioned 2.3–10 mm in from margin at widest point of blade, tertiary veins percurrent, more or less perpendicular to midvein, 1.9–5.6 mm apart at midleaf, intertertiary veins present, tertiary veins often joined by quaternary veins; adaxial leaf surface covered in well-developed bulla-based hairs completely covering the leaf areoles, widest hair bases to 2.1 mm, apices of bulla-based hairs mostly recurved toward the leaf margin, young leaf adaxial surface producing long-stemmed, clavate-dentritic hairs along the primary, secondary, and tertiary veins from between the bulla-based hairs, sessile, glandular hairs produced along the primary, secondary, tertiary, and quaternary veins between the bulla-based hairs; abaxial leaf surface covered in sparse bulla-based hairs, these spreading to erect, often crisped, those along the primary, secondary, and tertiary veins larger than hairs produced throughout the lamina, the lamina clearly visible, lamina appearing as a series of pits from depressions of the bulla-based hairs produced from the upper leaf surface, sessile, glandular hairs produced throughout the lamina and along veins; petioles 0.2–2.1 cm long, covered in ascending to spreading, bulla-based hairs on both surfaces. Inflorescences terminal, flowers mostly produced in glomerulate clusters, 3–42 flowered, 3.3–7.6 × 1.5–9.8 cm, the peduncle 0.4–2.2 cm long, proximal inflorescence branches 8–26 mm long; bracts narrowly ovate with a long attenuate apex, 1.2–2.2 mm long; bracteoles narrowly ovate with a long attenuate apex, 1.4–2 × 0.3–0.4 mm, glabrous. Flowers 5-merous, sessile or with pedicels to 0.5–0.9 mm long; hypanthium 3.2–3.4 mm long, short-oblong to globose, unlobed to slightly 5-lobed, but lobing mostly obscured by bulla-based hairs, slightly constricted below the torus, free portion of the hypanthium 0.5–0.6 mm long, abaxial surface covered in bulla-based hairs to 2.7 mm long, and sessile, glandular hairs; adaxial surface (i.e., free portion) covered in small, bulla-based hairs developing into androecial fringe; calyx teeth 2.2–2.7 × 0.4–0.6 mm, ascending or spreading, covered in bulla-based hairs; calyx lobes triangular, acute to rounded at apex, 0.4–2.2 × 0.8–2.5 mm, covered in bulla-based hairs abaxially and sessile, sparse, glandular hairs adaxially; calyx tube not tearing, 0.2–0.8 mm long with bulla-based hairs abaxially and sessile, glandular hairs adaxially; petals 5, 3.6–4.5 × 1.9–2.7 mm, white, ovate or elliptic with an acute apex and membranous margin, with 3–4 slightly bulla-based hairs produced abaxially, just below the apex, to 2.7 mm long; stamens 10; filaments 2.2–2.9 mm long, glabrous, anthers 1.2–2 mm long, with one dorsally oriented pore, anther thecae 1.1–1.8 mm long, anthers with a dorso-basal appendage 0.2–0.25 mm long; style 3.3–3.8 mm long, glabrous, slightly dilated in the middle, collar absent, style subtended by a crown of multicellular, linear to elongate-triangular (needle-like) hairs, which are slightly longer than the surrounding bulla-based hairs of the ovary apex, stigma punctate; ovary 2.4–3 × 3.8–4.5 mm, apex flat to slightly rounded, pubescent with bulla-based hairs, except for the linear or elongate-triangular hairs forming crown, placentation axile with deeply intruded placenta, 5-locular; berries globose, purple to violet at maturity, 5–6 mm long (including calyx tube), 5.3–7 mm wide, seeds 0.6–0.65 mm long, obpyramidal, slightly falcate, testa smooth, light brown, raphe dark amber, smooth, extending the length of the seed.

**Figure 14.**
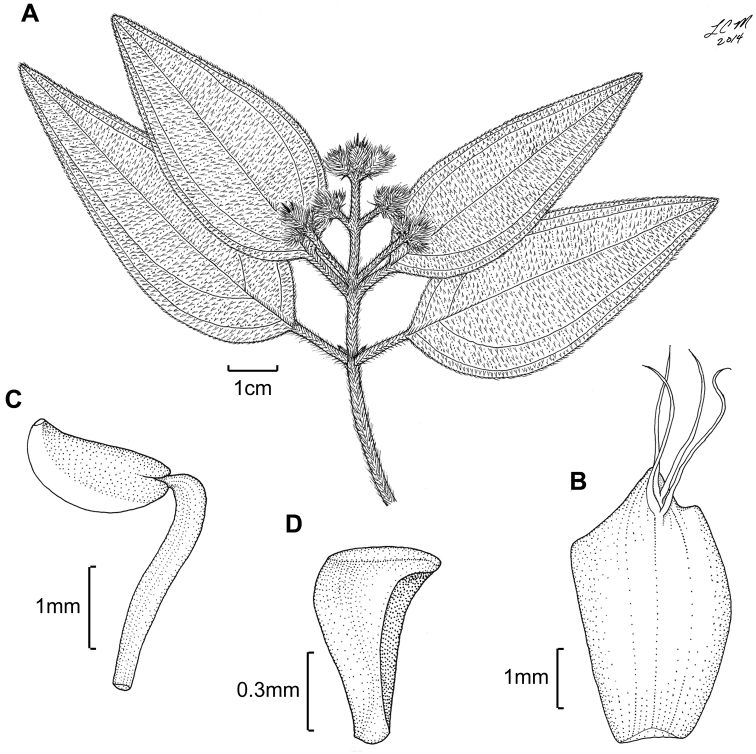
Illustration of *Miconia argentimuricata*. **A** Habit (*Ekman 3702*) **B** petal abaxial surface showing multiple, long, slightly bulla-based hairs (*Ekman 15940*) **C** stamen (*López-Figueiras 2180*), and **D** seed (*Ekman 15940*).

**Figure 15.**
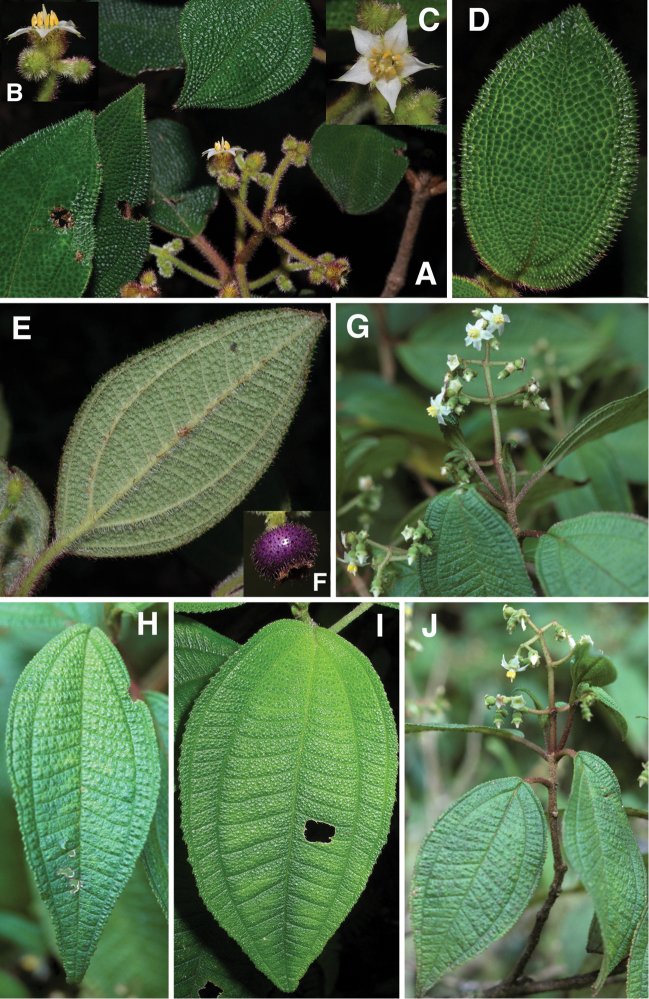
Photos of *Miconia argentimuricata* (**A–F**) and *Miconia asperifolia* (**G–J**). **A** Inflorescence of *Miconia argentimuricata* **B** side view of flower showing long, slightly bulla-based hairs on the petal abaxial surface **C** flower frontal view **D** leaf adaxial surface showing striking bulla-based hairs **E** leaf abaxial surface showing pitting **F** mature fruit (all from *Michelangeli 2219*) **G** inflorescence and flower frontal view of *Miconia asperifolia* (*Judd 5357*) **H** leaf adaxial surface showing well-developed bulla-based hairs (*Judd 5357*) **I** leaf adaxial surface showing poorly developed bulla-based hairs (*Ionta 2005*) **J** habit and inflorescence of *Miconia asperifolia* (*Judd 5357*). Photos **A–F** taken by F. Michelangeli and **G–J** by W.S. Judd.

##### Phenology.

*Miconia argentimuricata* has been collected in fruit from December through July and in flower from November through April.

##### Distribution

(Fig. 11). *Miconia argentimuricata* is restricted to eastern Cuba and can be found in both the Sierra Maestra (including Loma del Gato) and the Baracoa-Moa-Sierra de Cristal regions.

##### Ecology.

*Miconia argentimuricata* occurs from 600-1400 m in elevation and can be found on granitic soils (Sierra Maestra) or on serpentine soils in the northern part of its distribution (Moa region). The species occurs in lower elevation “manacales” (wet, montane rainforest; see Borhidi 1996) in the northern part of its range and higher elevation “monte frio” in the southern part of its range.

##### Conservation status.

Considering the wide range of *Miconia argentimuricata* and that it occurs in protected areas in both parts of its range (Parque Nacional Alejandro de Humboldt, Parque Nacional Pico Turquino, Reserva Ecológica Loma del Gaato-Monte Líbano), this species should be considered stable and possibly of least concern. However, some populations in the Moa region may be under threat from mining operations, and Bécquer (2007) suggested the species was near threatened.

##### Discussion.

Populations of *Miconia argentimuricata* from the Sierra Maestra are readily distinguished from those of the Baracoa and Moa regions by their more robust growth form (broadly elliptic leaves, larger inflorescences), as well as their darker, copper rather than silver color of the leaf adaxial surface (in herbarium specimens). The leaves are narrowly elliptic to often ovate with narrowly acute to acuminate apices in those specimens from the Baracoa-Moa-Sierra de Cristal region. However, morphological characters are sufficiently cohesive between the two regions to place those geographically separated populations within a single species.

Although Howard and Wright (1988) provided a microfiche of the lectotype of *Miconia argentimuricata* (as *Calycogonium muricatum*), he did not lectotypify the species explicitly in writing, and thus his lectotypification was incomplete. We have fully lectotypified the species with the specimen from GOET here. As specimens collected by Wright were often assigned species numbers by Asa Gray, as well as Wright’s collection numbers, one of the isolectotypes here subsequently has a different number on the label (*Wright 682*) than that given in the protologue (*Wright 2485*) for the species (see also discussion by Bécquer 2012).

##### Specimens examined.

**CUBA. Prov. Granma**. Buey Arriba: margenes del Arroyo Barrio Nuevo, 16 May 1988, *Álvarez & al. HFC-64613* (B, HAJB, JE); Buey Arriba. Alto del Escudero, 20 May 1988, *Álvarez & al. HFC-64782* (B, HAJB, JE); Buey Arriba. Alto del Rondón, 22 May 1988, *Álvarez & al. HFC-64999* (B, HAJB, JE); Oriente, Sierra Maestra, N slope of Punta de Palmamocha, 1250 m, 14 Jul 1922, *Ekman 14291* (S); Oriente, Sierra Maestra, on the divide between Río Yara and Río Palmamocha, towards one of the highest tributaries of Río Yara, 16 Jul 1922, *Ekman 14342* (US, S); Granma/Santiago de Cuba, Bartolome Masó/Guamá, Parque Nacional Turquino, sendero Alto de Naranjo-Pico Turquino, entre campamento de La Aguada y Pico Joaquìn, 9 Nov 2013, *Michelangeli et al. 2219* (NY). **Prov. Guantánamo.** Baracoa, Via Sur en las montañas prox. Fajá, 10 Feb 1952, *Acuña & Diaz-Barreto 17520* (HAC); Baracoa, Cañadas en la carretera de Quibiján, 1 Jan 1960, *Alain & López-Figueiras 7123* (HAC, HAJB); Baracoa, cañadas a lo largo Via Muluta, 1 Jan 1960, *Alain & López-Figueiras 7142* (HAC); Baracoa, cañadas a lo largo Via Muluta, 1 Jan 1960, *Alain & López-Figueiras 7145* (HAC); Baracoa, cañadas a lo largo Via Muluta, 1 Jan 1960, *Alain & López-Figueiras 7164* (HAC); Baracoa, carretera de Quibiján, en cañada, 1 Jan 1960, *Alain & López-Figueiras 7142* (HAC); Baracoa, carretera de Quibiján, en cañada, 1 Jan 1960, *Alain & López-Figueiras 7145* (HAC); Baracoa, carretera de Quibiján, en cañada, 1 Jan 1960, *Alain & López-Figueiras 7164* (HAC); Sierra de Imías. Lomas Jubal, 19 Aug 1975, *Álvarez & al. HFC-27511* (B, HAJB, JE); IBID *HFC-27598* (B, HAC, HAJB, JE); Baracoa. Loma Los Guineos, 400-500 msm, 14 Mar 1986, *Arias & al. HFC-58668* (B, HAJB, JE); Baracoa: Sierra de Imías, 800-1100 msm, 29 Aug 1971, *Bisse HFC-19543* (HAJB, JE); Baracoa. Montes al sur del Yunque, 9-10 Feb 1072, *Bisse HFC-21467* (B, HAJB, JE); Baracoa: pluviosilva al sur de la Loma del Yunque, 300-400 msm, Feb 1968, *Bisse & Köhler HFC-5025* (HAJB, JE); Baracoa: valle al noroeste del Yunque de Baracoa, Feb 1968, *Bisse & Köhler HFC-5197* (HAJB, JE); Baracoa: Mina Iberia, en las orillas del arroyo Iberia, 300 msm, Mar 1968, *Bisse & Köhler HFC-6363* (HAJB, JE); Baracoa: Sierra de Imías, 800-1000 msm, May 1968, *Bisse & Köhler HFC-8858* (B, HAJB, JE); IBID *HFC-9455* (HAJB, JE); Guantánamo: Monte Cristi, altiplano, 700 msm, May 1968, *Bisse & Köhler HFC-9335* (HAJB, JE); Aserrío Nuevo Mundo, falda oeste de la mina de Iberia, 200-300 msm, Apr 1975, *Bisse & González HFC-25753* (HAJB); Baracoa: Sierra de Imías, Loma de la Ciguapa, 1100 msm, Jun 1967, *Bisse & Rojas HFC-2560* (HAJB, JE); Baracoa, alrededores del poblado de Báez, 24 Jan 1977, *Bisse & al. HFC-33951* (B, HAJB, JE); Baracoa, Quibiján, Pluviosilva de la zona de Arroyo Blanco en el camino a Vega de la Palma, 16 Feb 1978, *Bisse & al. HFC-36916* (B, HAJB, JE); Baracoa, Quiviján, pluviosilva de la parte occidental de la Sierra Azul, 400-500 msm, 17 Feb 1978, *Bisse & al. HFC-37002* (B, HAJB, JE); Baracoa, Vega de La Palma, alrededores del río Duaba, 20 Feb 1979, *Bisse & al. HFC-39655* (B, HAJB, JE); Imías. Sierra de Imías, loma de Tres Piedras, 1000-1100 msm, 6 Apr 1984, *Bisse & al. HFC-52325* (HAJB); Yateras Palenque. Sierra del Frijol, cerca de Bernardo, 800 m, 17 May 1983, *Bisse & al.*, *HFC-49721* (B, HAJB, JE); Imías. Sierra de Imías, falda norte de la Loma de Tres Piedras, 900-1000 msm, 9 Mar 1984, *Bisse & al. HFC-52629* (B, HAJB, JE); Imías. Sierra de Imías. La Yamagua. Loma maestra de Yamagua, 750-850 msm, 14 Apr 1984, *Bisse & al. HFC-52878* (B, HAJB, JE); Imías, loma al oeste de las cabezadas del río Jojo, Sierra de Imias, 900-1060 msm, 19 Apr 1984, *Bisse & al. HFC-53491* (B, HAJB, JE); Oriente, Baracoa in collibus ad Taco Bay, 2 Dec 1914, *Ekman 3702* (NY, S); Baracoa, valle del río Joa, arriba de Baracoa, 30 Jan 1971, *Grudzinskaya 737* (HAC); Upper river valley of Río Navas, Oriente, 22 Mar 1910, *Shafer 4405* (NY); Side and top of El Yunque, Oriente, 20 Dec 1910, *Shafer 8006* (A, NY); El Yunque, Mt. Baracoa, 1 Mar 1903, *Underwood 1020* (NY). **Prov. Holguín.** Monte La Breña, Moa, Oriente, 5 Nov 1945, *Acuña 13274* (HAC, NY); Piloto, Moa, 6 May 1973, *Álvarez & Berazaín HFC-24388* (HAJB); Moa: Pluvisilva Km 8-10 del camino de La Melba, 1 May 1980, *Álvarez & al. HFC-42530* (B, HAJB, JE); Frank País. Falda norte de la Sierra Cristal, al suroeste de El Culebro, subida al Pico Cielo, 13 Apr 1987, 13 Apr 1987, *Bässler & al. HFC-61109* (B, HAJB, JE); Cuchillas de Toa: Sierra de Maguey, 700 msm, Apr 1970, *Bisse HFC-16787* (HAJB, JE); Moa: La Melba, pluviosilva de montaña cerca del aserrío, 500 msm, 27 Dec 1968, *Bisse & Lippold HFC-11144* (HAJB, JE); Moa, charrascales en el altiplano de la Sierra de Moa, 600-900 msm, Mar 1968, *Bisse & Köhler HFC-6651* (HAJB, JE); Moa: en las orillas del río Jiguaní, cerca del segundo aserrío de la Melba, Apr 1968, *Bisse & Köhler HFC-6772* (HAJB, JE); Km 10 de la carretera de La Melba, 100 msm, 19 Apr 1981, *Bisse & al. HFC-44528* (B, HAJB, JE); Aserrío Palenque, entre el río Cabonico, 400-800 msm, 2 May 1981, *Dietrich & al. HFC-45400* (B, HAJB, JE); Oriente, Sierra de Mícara, in manacales, 700 m, 13 Dec 1922, *Ekman 15927* (HAC, NY, S, US); Moa: camino desde Moa hacia La Melba, 30 Jan 1969, *Lippold HFC-12491* (HAJB, JE); IBID *HFC-12543* (HAJB, JE); Moa: La Melba, falda este de la Sierra de Moa, 500-800 msm, 5 Apr 1970, *Lippold HFC-16566* (HAJB, JE); Moa, Cuchillas de Moa, along road from Moa to La Melba, 24 Jun 2002, *Skean 4285* (FLAS, HAJB); **Prov. Santiago de Cuba.** Cima del Pico Turquino, Sierra Maestra, 10–26 Jun 1936, *Acuña 7712* (HAC); Mayarí, bosque húmedo en la falda sur del Cristal, 2–7 Abr 1956, *Alain et al. 5570* (HAC); Sierra Maestra: El Uvero, pluviosilva de la loma de La Francia, 800-1000 msm, 3 May 1972, *Bisse HFC-21318* (HAJB, JE); IBID, 3-5 Feb 1972, *HFC-21332* (HAJB, JE); Sierra Maestra: El Uvero, Loma Siberia, 900-1100 msm, 29 Mar 1969, *Bisse & Lippold HFC-13543* (HAJB, JE); Santiago de Cuba: falda este de Gran Piedra, 26 Apr 1969, *Bisse & Lippold HFC-14664* (HAJB, JE); Sierra Maestra: subida desde Pico Cardero hasta Pico Cuba, 1300-1800 msm, 10 May 1971, *Bisse & Lippold HFC-19017* (HAJB, JE); Sierra Maestra: firme de la Sierra entre Alcarraza y Punta de Lanza, 800-1000 msm, 28 Apr 1969, *Bisse & Lippold HFC-19685* (HAJB, JE); Loma del Gato, 8–10 Jul 1931, *Bucher 249* (HAC, NY); Oriente, Sierra de Cristal, in manacales at the headwaters of Rio Lebisa, 600–700 m, 14 Dec 1922, *Ekman 15940* (S); (HAC); Loma del Gato and vicinity, Cobre Range of Sierra Maestra, 1000 m, 11 Jul–14 Aug 1921, *León LS-10416* (HAC, NY); southern Oriente and Pico Turquino, Sierra Maestra, 1200–1400 m, 1 Jul 1922, *León LS-10948* (HAC, NY); márgenes del Arroyo Peladero Arriba, Finca la Valenzuela, 5–8 Apr 1955, *López-Figueiras 2179* (HAC); margenes del Arroyo Peladero Arriba, alto de la Valenzuela, Sierra Maestra, 5–8 Apr 1955, *López-Figueiras 2180* (HAC, HAJB, US); Sierra Maestra, Cordillera de Gran Piedra, 18 Mar 1956, *López-Figueiras 2613* (HAC); Sierra Maestra, Entre los arroyos Peladero e Indio, costa sur de Oriente, 900–1350 m, 27 Nov 1959, *López-Figueiras UO-390* (HAC); Sierra Maestra, Entre los arroyos Peladero e Indio, costa sur de Oriente, 3000–4500 ft, 27 Nov 1959, *López-Figueiras UO-396* (HAC, US).

#### 11. Miconia bullotricha

Taxon classificationPlantaeMyrtalesMelastomataceae

Bécquer & Majure, PhytoKeys 33: 65. 2014.

Fig. 16

##### Type.

CUBA. Prov. Guantánamo: Palenque. Bernardo. Sierra del Frijol, loma Bernardo, 800–900 m, 21 May 1983, *J. Bisse, C. Beurton, H. Dietrich, J. Gutiérrez, L. Lepper, R. Dolmus, E. Köhler, R. Rankin, I. Arias, HFC-49930* (holotype: HAJB! [HAJBG000701]; isotypes: B! [B100362845], HAJB! [HAJBG000700], JE! [JE00022358], NY! [NY01796819]).

##### Description.

Evergreen shrub (height unknown); stems round in cross section, not ridged, the internodes 1.1–2.4 cm long; stems densely covered in bulla-based hairs with strongly to narrowly dilated bases, to 0.3 mm long, the hairs spreading to descending with apices recurved upwards, young stem hairs often dark purple in color; nodal line inconspicuous, present. Leaves opposite, decussate, elliptic to ovate-elliptic, often slightly falcate, 4.2–6 × 1–2.2 cm, often slightly anisophyllous, yellowish when dried; apex narrowly acute; base rounded to broadly cuneate or abruptly cuneate; margin revolute, dentate, the dentations obscure, each covered in one large, bulla-based hair, venation acrodromous, 3 (–5)-veined, 1 primary vein and 1 (rarely 2) pairs of suprabasal secondary veins, often asymmetrical at union with midvein, produced 2–6 mm from the leaf base, positioned 0.7–3 mm in from margin at widest part of blade, the tertiary veins percurrent, more or less perpendicular to midvein, 2–3 mm apart at mid-leaf, intertertiary veins present, often joined by quaternary veins; adaxial leaf surface with primary and secondary veins impressed, tertiary veins flat to slightly impressed, remaining veins flat, abaxial surface with primary, secondary and tertiary veins raised, the higher order veins more or less flat to slightly raised (i.e., clearly visible to more or less obscure); adaxial leaf surface completely covered in erect bulla-based hairs, these fully expanded at the base, thus the lamina obscured, widest hair bases to 1.5 mm wide, hair apices acute to truncate, sometimes slightly recurved toward the leaf margin, sessile, glandular hairs occurring between the bases of bulla-based hairs; abaxial leaf surface nearly completely covered with bulla-based hairs with strongly to narrowly dilated bases, the lamina areoles not completely filled, the hairs along the epidermis erect with apices recurved or not, veins completely covered by spreading to erect hairs mostly with narrowly dilated bases and recurved apices, sessile, glandular hairs occurring throughout the lamina, as well as along veins; acarodomatia inconspicuous, of multicellular, linear hairs present in the axils of the primary and secondary, as well as primary and tertiary veins; petiole 5–8 mm long, covered in spreading bulla-based hairs, those of the adaxial surface slightly longer and narrower than those of the abaxial surface and recurved towards to the leaf blade. Inflorescences terminal, well-developed to reduced cymes of 3–13 flowers, 2–3.5 × 1.8–3.4 cm, the peduncle 0.7–1.4 cm long, usually conspicuously reflexed at base, thus the entire inflorescence pendant, the proximal inflorescence branches 0.5–1 cm long; bracts oblong to narrowly ovate, 1.1–2 mm long; bracteoles narrowly ovate, ca. 0.5–0.7 × 0.2–0.3 mm. Flowers 4-merous, pedicels 0–1 mm long. Hypanthium ca. 1.6 × 2.8 mm, globose, slightly constricted below torus, abaxial surface covered in granulate, bulla-based hairs with dilated bases and attenuate to truncate apices, to 0.5 mm long, and sessile, glandular hairs, the free portion of hypanthium 0.5–0.7 mm long, adaxial surface longitudinally ridged and covered by bulla-based hairs; calyx teeth 1.75–2.2 × 0.5 mm, linear and terete, recurved upon maturation, covered in bulla-based hairs; calyx lobes ca. 1 × 1.3 mm, triangular, apices acute, with bulla-based hairs abaxially and sessile, glandular hairs produced adaxially; calyx tube not tearing, ca. 0.4 mm long, with bulla-based hairs abaxially, sessile, glandular hairs adaxially and clavate-dendritic hairs produced at the apex; petals 4, immature (i.e., only seen in bud), ovate to elliptic, with acute apices, apices with one, slightly bulla-based hair produced subapically, hair to 0.5 mm long; stamens 8 (immature), filaments glabrous, anthers ovate, with a well-developed dorso-basal appendage and one apically-oriented pore (the pore position could be an artifact of level of maturity); style (immature) dilated in the middle, subtended by a short crown of multicellular hairs, these only slightly longer than the surrounding bulla-based hairs on the ovary apex; stigma punctate; ovary ca. 1.4 × 2.4 mm, apex flat, covered in bulla-based hairs, 4 locular, with axillary placentation, the placenta deeply intruded into locule; berries (immature) globose, ca. 3–3.4 × 3 mm, color at maturity unknown, but probably more or less purple; seeds (immature) 0.2–0.6 mm long, obpyramidal, testa smooth, light brown, raphe extending the length of the seed, dark brown.

**Figure 16.**
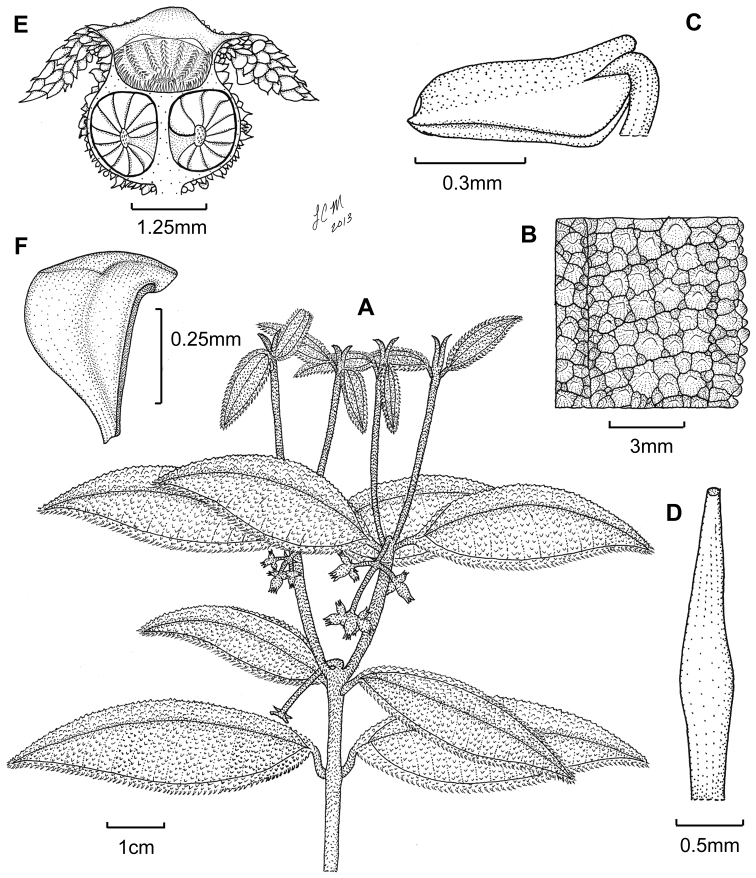
Illustration of *Miconia bullotricha*. **A** habit **B** close-up of leaf adaxial surface **C** immature stamen **D** style **E** fruit longitudinal section **F** seed (all from *Bisse et al. HFC-49930*). Reproduced with permission from Majure et al. (2014a).

##### Phenology.

Plants with buds and young fruits have been collected in May.

##### Distribution

(Fig. 11). *Miconia bullotricha* is restricted to the mountains of the Guantánamo province, Cuba.

##### Ecology.

*Miconia bullotricha* occurs in semi-dry, montane and elfin forest on serpentine soils at elevations of 500–1000 m. Associated melastomes include *Calycogonium grisebachii* Triana, *Miconia baracoensis* Urb. and *Miconia echinata* Judd et al.

##### Conservation status.

We do not have extensive knowledge of numbers of individuals per population or the reproductive biology of this species, so the conservation status of *Miconia bullotricha* cannot be critically evaluated at this time and should be considered data deficient. More fieldwork is imperative before its status can be assessed.

##### Discussion.

**Figure 17.**
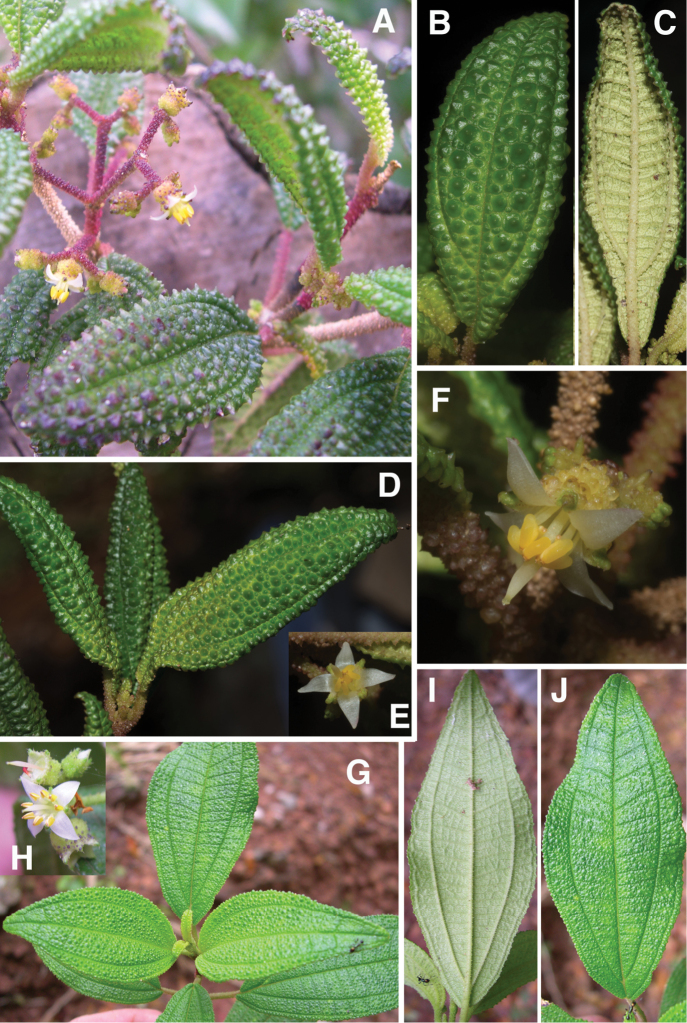
Photos of *Miconia ottoschmidtii*. **A** Inflorescence (*Bécquer s.n.*) **B** leaf adaxial surface (*Michelangeli 2234*) **C** leaf abaxial surface (*Michelangeli 2228*) **D** developing leaves showing adaxial surface (*Michelangeli 2228*) **E** flower frontal view (*Michelangeli 2234*) **F** close-up of flower (**E–F** from *Michelangeli 2234*) **G** *Miconia ottoschmidtii* from Pinar del Río showing poorly developed bulla-based hairs **H** flower and immature fruit **I** leaf abaxial surface **J** leaf adaxial surface (**G–J** from *Bécquer HFC-82434*). Photo **A** taken by E. Bécquer, **B–F** taken by F. Michelangeli, and **G–J** taken by J.R. Abbott.

Although Majure et al. (2014) compared *Miconia bullotricha* to the very phenetically similar *Miconia ottoschmidtii* (Figs 12E–H, 17), and several characters overlap with that species, it is possible that *Miconia bullotricha* may be more closely related to members of the *Acuminata* subclade (Majure et al. 2015), specifically *Miconia granulata*. *Miconia bullotricha* shares the inflorescence structure (Fig. 9O, 16) of *Miconia granulata*, which also may have pendant inflorescences in some specimens, as well as 4-merous flowers. Vegetatively, material of *Miconia bullotricha* can be sometimes confused with *Miconia argentimuricata*, also part of the *Acuminata* subclade. Likewise, Majure et al. (2014a) mistakenly cited a specimen of *Miconia argentimuricata* as *Miconia bullotricha* (*Bisse et al. HFC-49721*; B, HAJB, JE). Material of this poorly known species will need to be incorporated into phylogenetic analyses in order to confirm its placement.

##### Additional specimens examined.

**CUBA. Prov. Guantánamo**: Baracoa. Imías, Sierra de Imías, loma Jubal (al sur de Los Lechugos), 900–1000 m, 19 Aug 1975, *Álvarez & al. HFC-27626* (B, HAC, HAJB, JE); Baracoa. Sierra de Purial, La Gurbia, 700 m, May 1968, *Bisse & Rojas HFC-8562* (HAJB, JE); IBID, *HFC-9389* (HAJB, JE); Baracoa. Falda suroeste de la loma del Mirador, 500 m, 9 Aug 1975, *Bisse & Meyer HFC-27230* (B, HAC, HAJB, JE); Yateras Palenque. Sierra del Frijol, cerca de Bernardo, 800 m, 17 May 1983, *Bisse et al. HFC-49731* (B, HAJB, JE).

#### 12. Miconia ottoschmidtii

Taxon classificationPlantaeMyrtalesMelastomataceae

(Urb.) Majure & Judd, J. Bot. Res. Inst. Texas. 7: 269. 2013.

Figs 12E–H , 17

Ossaea ottoschmidtii Urb., Repert. Spec. Nov. Regni Veg. 24: 6. 1927. Type: CUBA. Guantánamo, La Prenda, [day not given] April 1889, *H.F.A. von Eggers 5332* (lectotype: M! [M0165772], designated here; isolectotypes: BR! [BR0000013239633, all but branch at top center of sheet with yellow leaves], HAC!).

##### Type.

Based on *Ossaea ottoschmidtii* Urb.

##### Description.

Evergreen shrub, 1–1.5 m tall; stems round in cross section, not ridged, the internodes 0.5–4.9 cm long, stem indumentum of spreading, granulate, bulla-based hairs to 0.4 mm long; nodal line present. Leaves opposite, decussate, rarely ovate to mostly narrowly elliptic, 2.4–7.9 × 1–3.1 cm, slightly anisophyllous, apex acute to slightly acuminate, base acute, venation acrodromous, 5-veined, the midvein and 2 pairs of arching secondary veins, the outermost sometimes intramarginal, secondary veins mostly basal to suprabasal, the innermost pair suprabasal, produced 0.8–25 mm from leaf base, positioned 1.6–4.7 mm in from margin at widest point of blade, tertiary veins percurrent, more or less perpendicular to midvein, 1.4–4.3 mm apart at midleaf, intertertiary veins rarely present, tertiary veins usually joined by conspicuous quaternary veins; adaxial leaf surface covered in well-developed, dorsally compressed bulla-based hairs, widest hair bases to 1.8 mm, these mostly to entirely covering the leaf areoles, apices of bulla-based hairs mostly erect, young leaf adaxial surface producing long-stemmed, clavate-dentritic hairs along the primary, secondary, and tertiary veins from between the bulla-based hairs, sessile, glandular hairs produced along the primary, secondary, tertiary, and quaternary veins between the bulla-based hairs; abaxial leaf surface covered in bulla-based hairs, these erect along the lamina, spreading along the primary and secondary veins, and recurved towards the leaf margin along the tertiary veins, those along the primary, secondary, and tertiary veins larger than hairs produced throughout the lamina, the lamina visible, appearing as a series of pits from depressions of the bulla-based hairs produced from the upper leaf surface, sessile, glandular hairs produced throughout the lamina and along the veins, domatia present, consisting of tufts of bulla-based hairs, at the axils of the primary and secondary, as well as primary and tertiary veins; petioles 0.3–2.1 cm long, covered in granulate, bulla-based hairs on the abaxial surface and long, spreading, bulla-based hairs on the adaxial surface. Inflorescences terminal, cymose, flowers mostly produced in glomerulate clusters, 19–25 flowered, 2.2–6.5 × 1.3–4 cm, the peduncle 0.1–1.9 cm long, proximal inflorescence branches 3–20 mm long; bracts oblong to ovate, 0.6–1.7 mm long; bracteoles ovate, 0.3–0.7 × 0.2–0.6 mm. Flowers 4-merous, with pedicels 0.2–2 mm long; hypanthium 1.4–2.3 mm long, short-oblong to globose, strongly 4-lobed, clearly constricted below the torus, free portion of the hypanthium 0.5–0.9 mm long, abaxial surface covered in bulla-based hairs to 0.3 mm long, and sessile, glandular hairs near the bases of the bulla-based hairs; adaxial surface (i.e., free portion) covered in small, bulla-based hairs; calyx teeth 0.4–0.8 × 0.2–0.4 mm, erect to spreading, covered in bulla-based hairs; calyx lobes 0.2–1.1 × 1.2–1.5 mm, triangular, broadly acute to rounded at apex, covered in bulla-based hairs abaxially and sessile, glandular hairs adaxially; calyx tube not tearing, 0.4–0.8 mm long with bulla-based hairs abaxially and sessile, glandular hairs adaxially; petals 4, 2.9–3 × 1.4–1.7 mm, white, ovate to elliptic, with an acute apex, with 1–2 slightly bulla-based hairs produced abaxially, just below the apex, to 0.5 mm long; stamens 8; filaments 1.3–1.6 mm long, glabrous, anthers 1.4–1.5 mm long, with one dorsally oriented pore, anther thecae 1.1–1.2 mm long, anthers with a dorso-basal appendage 0.3 mm long; style 3.5–3.7 mm long, glabrous, not or only slightly dilated in the middle, collar absent, style subtended by a crown of multicellular, linear to elongate-triangular (needle-like) hairs, which are slightly longer than the surrounding bulla-based hairs of the ovary apex, stigma punctate; ovary 1.4–2 × 2.2–2.5 mm, strongly 4-lobed, apex flat, pubescent with bulla-based hairs, except for the linear or elongate-triangular hairs forming crown, placentation axile with deeply intruded placenta, 4-locular; berries globose, 4-lobed, purple at maturity, 2.7–3.6 mm long (including calyx tube), 3–4.8 mm wide, seeds 0.4–0.6 mm long, obpyramidal, testa smooth, light brown, raphe dark brown to black, smooth, extending the length of the seed.

##### Phenology.

*Miconia ottoschmidtii* has been collected in flower from January through July and in fruit from April through August.

##### Distribution

(Fig. 13). *Miconia ottoschmidtii* occurs in northern Cuba in Pinar del Río (Pan de Guajaibón), Mountain of Guamuhaya of central Cuba, the Sierra Maestra of southern/eastern Cuba and Sierra Cristal.

##### Ecology.

This species occurs on montane evergreen forest, wet montane rainforest, from 400–1050 m in elevation on fersialitic soils or rarely on serpentine soils (e.g., Sierra Cristal). Some associated melastomes are *Clidemia hirta* (L.) D.Don, *Conostegia icosandra* (Sw. ex Wikstr.) Urb., Mecranium integrifolium (Naudin) Triana subsp. integrifolium, *Meriania albiflora*, *Miconia matthaei* Naudin and *Miconia prasina* (Sw.) DC.

##### Conservation status.

This species is widespread throughout Cuba occurring in Parque Nacional Pico Turquino, Parque Nacional Pico Cristal, Reserva Ecológica Lomas de Banao, Reserva Ecológica Pico San Juan, Parque Nacional Topes de Collantes and Elemento Natural Destacado Pan de Guajaibón, among other non-protected areas. However, the species is not abundant throughout most of its distribution, and is only known from one collection each from Pico Cristal and Pan de Guajaibón. The habitat for this species has been reduced in size and quality, thus, we propose a category of endangered for *Miconia ottoschmidtii*.

##### Discussion.

*Miconia ottoschmidtii* is the only Cuban member of an otherwise Hispaniolan clade containing *Miconia lima*, *Miconia limoides*, *Miconia paralimoides* and *Miconia pedunculata* (Fig. 2), as well as several other putative members of the clade (*Miconia phrynosomaderma*, *Miconia marigotiana*, also Hispaniolan endemics). Of these taxa, *Miconia ottoschmidtii* is most phenetically similar to *Miconia lima* but can be differentiated from that species by the size of nearly all parts of the plant, with *Miconia lima* being the larger of the two species. Also, *Miconia lima* has broadly elliptic leaves, whereas those of *Miconia ottoschmidtii* are narrowly elliptic (length/width ratio 1.3–4.6 cm in *Miconia ottoschmidtii* and 1.5–2.2 cm in *Miconia lima*). Several specimens of *Miconia ottoschmidtii* exhibit longer hairs along the stems and hypanthia than the typical granulate hairs generally exhibited by the species (i.e., *Ekman 15794, 15912*, *Bécquer HAC-82434*), and these plants are distributed more or less throughout the distribution of *Miconia ottoschmidtii*. It should be noted that the specimen, *Bécquer HAC-82434*, is the northernmost and westernmost collection of the species in Cuba from Pinar del Río, Pan de Guajaibón, and is disjunct from other collections to the southeast in Guamuhaya montains. This population exhibits less well developed bulla-based hairs on the adaxial leaf surface and has larger and more broadly elliptic leaves (3.05–7.9 × 1.6–3.1 mm vs. 2.4–6.8 × 1–2.2 mm) than most populations from other regions of Cuba (Fig. 17E, F). It shows some phenetic similarity to *Miconia asperifolia*, but clearly is in a clade with another accession of *Miconia ottoschmidtii* from the Sancti Spiritus mountains (Majure et al. 2015; Fig. 2), again showing the lability of morphological characters in the *Lima* clade (see Majure et al. 2015).

The isolectotype sheet of *Miconia ottoschmidtii* at BR is a mixed collection that also contains a fragment of *Miconia norlindii* at the top center of the sheet.

##### Specimens examined.

**CUBA: Prov. Cienfuegos.** Cumanayagua. Sierra del Escambray, subida al Pico San Juan, 7 Nov 1987, *Arias & al. HFC-62962* (HAJB); Sierra del Escambray. Las Cuevas, 27 Feb 1995, *Jutierrez & Panfet HFC-71754* (HAJB); Complejo San Juan, Escambray, Cienfuegos, 2 Nov 1986, *Oviedo et al. s.n.* (HAC); **Prov. Granma.** Sierra Maestra, Falda sur Pico Turquino, *Acuña 12936* (HAC); Buey Arriba. Alrededores del poblado Barrio Nuevo, 1400 msm, 10 May 1988, *Álvarez & al. HFC-63793* (B, HAJB, JE); Buey Arriba. En la zona de las 120, 12 May 1988, *Álvarez & al. HFC-64145* (B, HAJB, JE); Buey Arriba. Pico Verde, 21 May 1988, *Álvarez & al. HFC-64954* (B, HAJB, JE); A lo largo del camino de Minas del Frío a Montpie, 22 Apr 1978, *Bisse & al. HFC-37232* (B, HAJB, JE); Pluviosilva de la zona de Meriño en la subida al Pico Caracas, 700-1000 msm, 24 Apr 1978, *Bisse & al. HFC-37438* (B, HAJB, JE); Valle del arroyo Escondido, 700-1000 msm, 26 Apr 1978, *Stohr HFC-37624* (B, HAJB, JE); B. Masó. Estribo de la falda norte de la Sierra Maestra, al este del Brazón de Santana, 700-1000 msm, *Bisse & al. HFC-40386* (B, HAJB, JE); Sierra Maestra, on the water divide between Rio Yara and Rio Plata, ca. 1050 m, 12 Jul 1922, *Ekman 14258* (GH, NY, S); Bartolomé Masó, Parque Nacional Turquino, sendero Alto de Naranjo-Pico Turquino, km 1, arriba de La Platica, 11 Nov 2013, *Michelangeli et al. 2234* (NY); Buey Arriba. Camino de barrio Nuevo a La Pata de la Mesa, 13 May 1988, *Sánchez HFC-64320* (B, HAJB, JE); Sierra Maestra, Monte La Bayamesa, Pico de Azua, 16 Jan 1987, *Savelev 209* (HAC). **Prov. Guantánamo**. Monte Verde, 1860–1864, *Wright 1233* (BR, GH, HAC, MO, NY, P, S). **Prov. Holguín.** Sierra de Cristal, headwaters of Río Lebisa, 12 Dec 1922, *Ekman 15912* (S); Margenes del Arroyo Peladero Arriba, alto de la Valenzuela, Sierra Maestra, 5–8 Apr 1955, *López-Figueiras 2221* (HAC, HAJB, US). **Prov. Pinar del Río.** Bahia Honda, Pan de Guajaibón, sendero de la ladera norte, 8 May 2004, 400–600 m, *Bécquer HFC-82434* (FLAS, HAJB, NY); **Prov. Sancti Spíritus.** Fomento. Caballete de Casas, 400-700 msm, 8 Nov 1979, *Bisse & al. HFC-40967* (B, HAJB, JE); Loma Caballete de Casas, 400-700 msm, 8 Nov 1979, *Herrera & Imchanitzkaja IMK-429* (HAC); top of Gloria Hill, Banao Mts., Santa Clara, 950 m, 30 Jul 1918, *León LS-7964* (HAC, NY); Santa Clara, Mts. of Trinidad slopes of Pico Potrerillo, 700–900 m, 12 Jun 1922, *Ekman 13990* (A, F, HAJB, MO, S); Santa Clara, Lomas de Banao, El Purial on Rio Banao, in rocky places near the top of El Purial, ca. 850 m, 27 Jan 1923, *Ekman 16226* (S); Santa Clara, Lomas de Banao, El Purial on Rio Banao, edge of forest at Los Guineos, ca. 800 m, 27 Jan 1923, *Ekman 16268* (S); Buenos Aires, Trinidad Hills, Gaviñas Ranch, 2500–3500 ft, 6 Mar 1929, *Jack 7011* (A, GH, HAC, NY); Lomas de Banao, 1 Jan 1920, *Luna 93* (HAC, NY); Banao, Loma de Banao, Tetas de Juana, 600–840 m, 15 Mar 2003, *Pipoly et al. 24812* (FTG). **Prov. Santiago de Cuba.** Sierra de Cobre, Loma del Gato, 25 Sept - 5 Oct 1935, *Acuña 9855* (HAC); Guamá. Entre La Alcarraza y Punta de Lanza, 30 May 1988, *Álvarez & al. HFC-65636* (B, HAJB, JE); Sierra Maestra: firme de la Sierra entre Alcarraza y Punta de Lanza, 800-1000 msm, 28 Apr 1969, *Bisse & Lippold HFC-19643* (HAJB, JE); IBID *HFC-19679* (HAJB); Loma del Gato, Cobre Range, Sierra Maestra, 900 m, Dec 1943, *Chrysogone & Clemente NSC-3193* (GH, HAC, NY); Gran Piedra Mts., 1 Jun 1949, *Clemente NSC-6620* (GH, HAC, US); Sierra Maestra, inter Finca Reunion el Loma del Gato, ca. 750 m, 29 Mar 1916, *Ekman 6926* (NY, S, US); Sierra Maestra, supra Firmeza, ca. 1000 m, 10 Nov 1917, *Ekman 8889* (NY, S); Sierra Maestra, Loma del Gato, 750 m, 9 Nov 1922, *Ekman 15680* (S, US); Monte [Libano] near Monterus, ca. 500 m, 27 Nov 1922, *Ekman 15794* (S); Loma del Gato, Cobre Range of Sierra Maestra, 950 m, 11 Jul–14 Aug 1921, *León LS-10046* (GH, HAC, NY); Southern Oriente and Pico Turquino, Sierra Maestra, 1 Jul 1922, *León LS-10945* (HAC, NY); zona de la Gran Piedra (Santiago de la Cuba), 10 Mar 1954, *López-Figueiras 1076* (HAC, HAJB, US); Sierra Maestra, en la zona “del Gato” 6 Jun 1954, *López-Figueiras 1421* (HAC, HAJB); Cordillera de la Gran Piedra, Sierra Maestra, 1200 m, 25 Mar 1956, *López-Figueiras 2643* (HAC, HAJB); Gran Piedra Range, Sierra Maestra, 900 m, 22 Apr 1956, *López-Figueiras 2692* (HAC, HAJB, US); Guamá, Parque Nacional Turquino, sendero entre Pico Joaquín y El Cojo, cerca del Cojo, 10 Nov 2013, *Michelangeli et al. 2228* (NY).

#### 13. Miconia lima

Taxon classificationPlantaeMyrtalesMelastomataceae

(Desr.) M.Gómez, Anales Hist. Nat. 23: 69. 1894.

**Figure 18.**
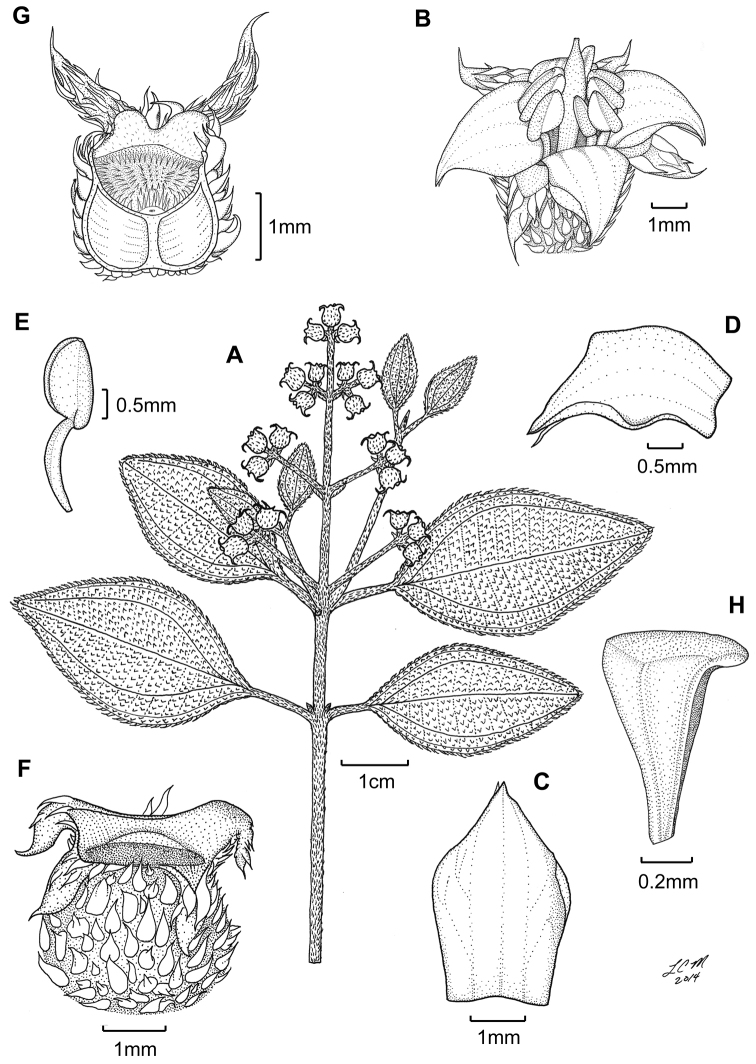
*Miconia lima*. **A** habit (*Ekman H1635*) **B** flower (*Judd 6587*) **C** petal side view (*Clase 935*) **D** petal adaxial surface (*Judd 6587*) **E** stamen (*Howard 12300*) **F** fruit longitudinal section (*Judd 5172*) **G** fruit (*Holdridge 1404*) **H** seed (*Judd 5172*).

**Figure 19.**
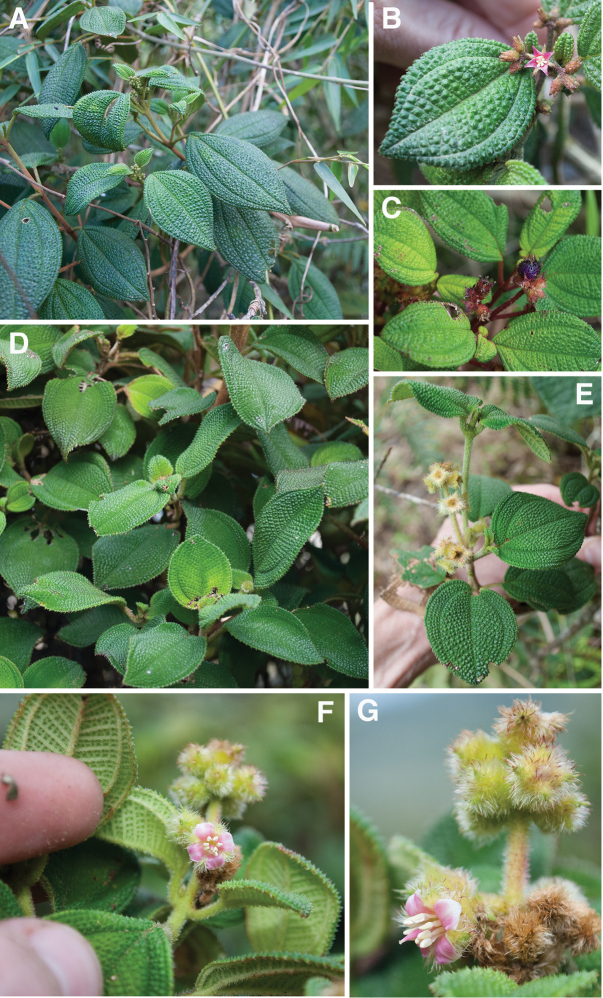
Photos of *Miconia lima* (**A–C**) and *Miconia limoides*. (**D–G**). **A** habit and adaxial leaf surface of *Miconia lima*, also with immature inflorescences (*Majure 5983*) **B** flowering branch of *Miconia lima* showing dark pink petals (*Majure 6020*) **C** fruiting branch of *Miconia lima* with dark purple fruit (*Majure 6036*) **D** vegetative material of *Miconia limoides* showing habit (*Majure 5959*) **E** fruting branch of *Miconia limoides* also showing reflexed stem hairs (*Majure 5958*) **F** flower of *Miconia limoides* (frontal view) showing pink petals and radially disposed stamens **G** typical compact inflorescence and side view of flower of *Miconia limoides* showing pink style and expanded middle portion of style (**F–G** from *Majure 5959*). All photos taken by L.C. Majure.

**Figure 20.**
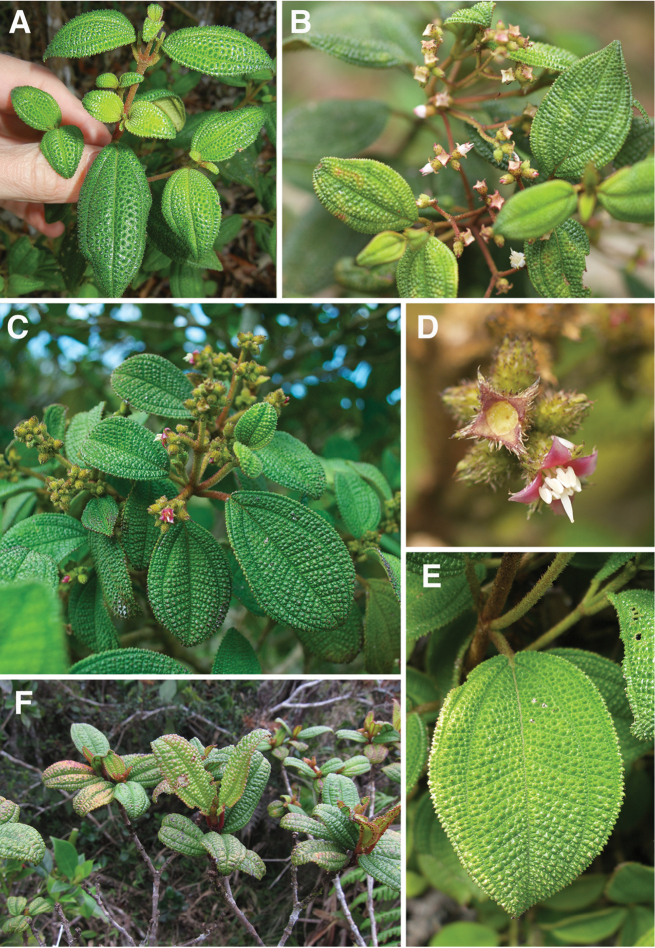
Photos of *Miconia lima* (**A–E**) and *Miconia pagnolensis* (**F**). **A** *Miconia lima* from Massif de la Selle, Haiti showing habit and ascending stem hairs (*Majure 4334*) **B** expanded infloresence and flowers showing whitish-pink petals (Sierra de Bahoruco, DR; *Skean 4312*), **C–E**) “Monteada Nueva” form of *Miconia lima* from the Dominican Republic **C** habit, inflorescence structure and stem showing ascending hairs (*Majure 5960*) **D** flower with dark pink petals showing an thers with a dorso-basal appendage and immature fruit with pinkish calyx lobes **E** leaf adaxial surface and stem with ascending hairs (**D–E** from *Judd 8083*) **F** *Miconia pagnolensis* sp. nov. from the type specimen showing habit and bulla-based hairs of the adaxial leaf surface (*Timyan 27*). Photos **A** and **C** taken by L.C. Majure, **B** by J.D. Skean, Jr., **D–E** by W.S. Judd and **F** by J. Timyan.

Figs 18 , 19A–C , 20A–E

Melastoma lima Desr., Encycl. [Lamarck et al.] 4: 47. 1797. Type: [HAITI]. St. Domingue, *M. Martin s.n.* (lectotype: MPU! [MPU013839], designated here).
Clidemia lima (Desr.) DC., Prodr. [A. P. de Candolle] 3: 161. 1828. Type: Based on Melastoma lima Desr.
Sagraea lima (Desr.) Naudin, Ann. Sci. Nat., Bot. sér. 3, 18: 99. 1852. Type: Based on Melastoma lima Desr.
Calycogonium lima (DC.) Griseb., Cat. Pl. Cub. [Grisebach] 95. 1866. Type: Based on Melastoma lima Desr.
Ossaea lima (Desr.) Triana, Trans. Linn. Soc. London 28: 147. 1871–1872. Type: Based on Melastoma lima Desr.
Leandra lima (Desr.) Judd & Skean, Bull. Florida Mus., Biol. Sci. 36: 61. 1991. Type: Based on Melastoma lima Desr.
Ossaea lima (Desr.) Triana var. grandifolia Cogn., Monogr. Phan. [A.DC. & C.DC.] 7: 1061. 1891. Type: [DOMINICAN REPUBLIC]. “In Santo Domingo, in summo Isabel de la Torre”, *H.F.A. von Eggers 2743* (lectotype: BR! [BR0000013239701], designated here).
Ossaea lima (Desr.) Triana var. ovalifolia Cogn., Symb. Antill. (Urban) 7: 531. 1913. Type: DOMINICAN REPUBLIC. La Vega. Jarabacoa, 650 m, June 1912, *M. Fuertes 1625* (lectotype: BR! [BR0000025284560], designated here; isotypes: BR! [BR0000025284553], CAS [CAS0003715], F! [F1022644], GH! [GH00073117], K! [K000329541], NY! [NY00099702], US! [US00123691]).

##### Type.

Based on *Melastoma lima* Desr.

##### Description.

Evergreen shrub, 1.5–5 m tall; stems round in cross section, not ridged, the internodes 0.3–6.3 cm long, stem indumentum of granulate to long, attenutate bulla-based hairs to 1.5 mm long, these ascending, appressed; nodal line present. Leaves opposite, decussate, elliptic to slightly obovate, 0.7–5.7 × 0.42–3.2 cm, slightly anisophyllous, apex acute, acuminate or obtuse, base acute to rounded, venation acrodromous, 5-veined, the midvein and 2 pairs of arching secondary veins, the outermost sometimes intramarginal, secondary veins mostly basal to suprabasal, the innermost pair suprabasal, produced 0.5–10 mm from leaf base, positioned 1.2–6 mm in from margin at widest point of blade, tertiary veins percurrent, more or less perpendicular to midvein, 1.4–3.9 mm apart at midleaf, intertertiary veins present, tertiary veins often joined by quaternary veins; adaxial leaf surface densely covered in well-developed bulla-based hairs, these covering the leaf areoles, widest hair bases to 2.7 mm, apices of bulla-based hairs mostly erect to recurved towards to the leaf margin, young leaf adaxial surface producing long-stemmed, clavate-dentritic hairs along the primary, secondary, and tertiary veins from glandular hairs produced along the primary, secondary, tertiary, and quaternary veins between the bulla-based hairs; abaxial leaf surface covered in bulla-based hairs, these erect to spreading, oftentimes ascending along the primary and secondary veins, those along the primary, secondary, and tertiary veins larger than hairs produced throughout the lamina, the lamina visible to nearly obscured, lamina appearing as a series of pits from depressions of the bulla-based hairs produced from the upper leaf surface, sessile, glandular hairs produced throughout the lamina and along all primary, secondary and tertiary veins; petioles 0.3–2.1 cm long, covered in ascending, bulla-based hairs on both surfaces, those on the adaxial surface slightly longer. Inflorescences terminal, 5–43 flowered cymes, 1.9–6.5 × 1–6 cm, the peduncle absent to 2.5 cm long, proximal inflorescence branches 3–23 mm long; bracts oblong to ovate with an attenuate apex, 1.3–2.7 mm long; bracteoles 0.8–2.2 × 0.2–0.6 mm, narrowly ovate with an attenuate apex, glabrous or with a few bulla-based hairs. Flowers 4–5-merous, with pedicels to 0.9 mm long; hypanthium 2–3.3 mm long, short-oblong to globose, 4–5-lobed, but lobing mostly obscured by bulla-based hairs, slightly constricted below the torus, free portion of the hypanthium 0.5–1 mm long, abaxial surface covered in bulla-based hairs from 0.8–1.6 mm long, and occasional, sessile, glandular hairs near the bases of the bulla-based hairs; adaxial surface (i.e., free portion) covered in small, bulla-based hairs; calyx teeth 1.2–3.2 × 0.3–0.6 mm, ascending, spreading or deflexed, covered in bulla-based hairs; calyx lobes 1–1.5 × 1.5–2 mm, more or less triangular, apex acute to obtuse, covered in bulla-based hairs abaxially and sessile, sparse, glandular hairs, and occasionally, clavate-dendritic hairs adaxially; calyx tube not tearing, 0.3–0.7 mm long with bulla-based hairs abaxially and sessile, glandular hairs adaxially, clavate-dendritic hairs often produced at the tube apex; petals 4–5, red to pink or pinkish-red, elliptic, 3–4.3 × 1.9–2.6 mm, with an acute apex and slightly membranous margin, with one or two slightly bulla-based hairs produced abaxially, just below the apex, often two bulla-based hairs nearly identical and appearing as one hair with a cordate base, to 1.5 mm long; stamens 8–10; filaments 1.2–2.1 mm long, glabrous, anthers 1.4–1.9 mm long, with one dorsally oriented pore, anther thecae 1.1–1.6 mm long, anthers with a dorso-basal appendage 0.25–0.4 mm long; style 4.7–4.9 mm long, glabrous, slightly dilated in the middle, collar absent, style subtended by a crown of multicellular, elongate-triangular (needle-like) hairs, which are slightly longer than the surrounding bulla-based hairs of the ovary apex, stigma punctate; ovary 1.5–2.5 × 2.3–3.4 mm, apex flat, pubescent with bulla-based hairs, except for the linear or elongate-triangular hairs forming crown, placentation axile with deeply intruded placenta, 4–5-locular; berries globose, 4–5-lobed, purple to purple-black at maturity, 2–3.5 mm long (including calyx tube), 2–3.5 × 3–4.1 mm wide, seeds 0.6–0.8 mm long, obpyramidal, testa smooth, light brown, raphe light brown, smooth, extending the length of the seed.

##### Phenology.

*Miconia lima* has been collected in flower and fruit from Februrary throught November.

##### Distribution

**Figure 21.**
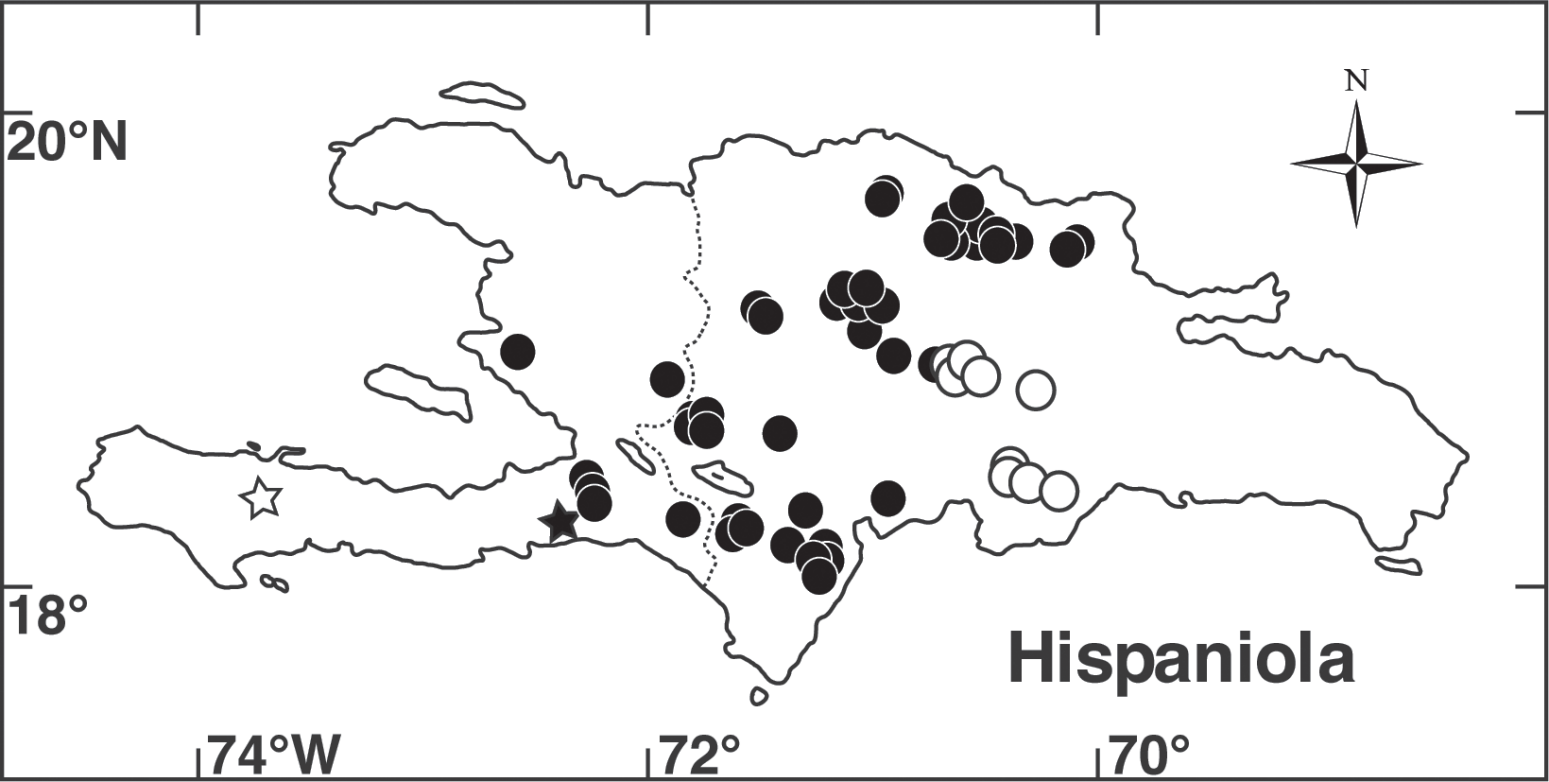
Distribution of *Miconia marigotiana* (closed star), *Miconia lima* (closed circles), *Miconia pedunculata* (open circles) and *Miconia pagnolensis* sp. nov. (open star).

**Figure 22.**
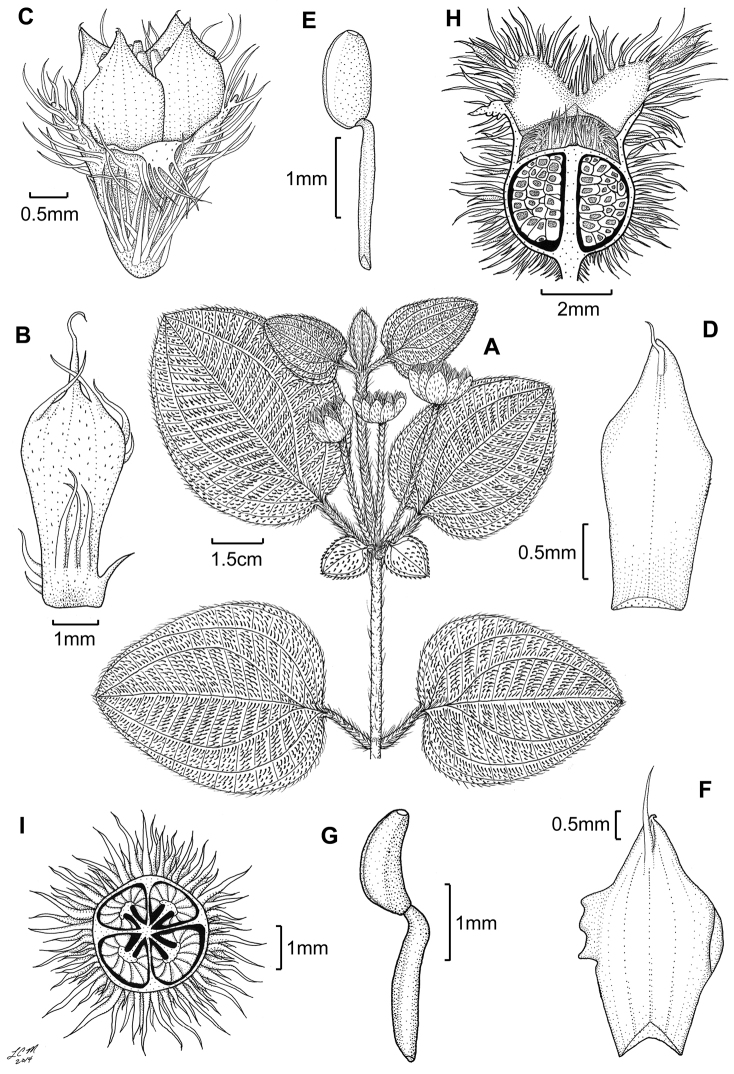
*Miconia pedunculata* (**A–E**) and *Miconia limoides* (**F–H**). **A** habit of *Miconia pedunculata* (*Pimentel 806*) **B** bracteole (*Zanoni 40212*) **C** flower (*Judd 6633*) **D** petal abaxial surface (*Zanoni 28291*) **E** stamen (*Zanoni 28291*) **F** petal abaxial surface of *Miconia limoides* (*Zanoni 18900*) **G** stamen (*Zanoni 18900*) **H** fruit longitudinal section (*Ekman 9579*).

(Fig. 21). *Miconia lima* is the most widespread species of all of the members of the *Lima* clade occurring widely throughout Hispaniola on almost all mountain ranges from 550 to 2000 m in elevation. It is known from the Massif de la Selle, Massif du Cahos, and Massif de Matheux in Haiti and the Sierra de Baoruco, Sierra de Neiba, Cordillera Central and Cordillera Septentrional in the Domincan Republic (Fig. 22). *Miconia lima* was cited by Alain (1957) as occurring on Cuba, based on a misidentification of the Cuban species *Miconia tentaculicapitata* (*Linden 2102*; BR, K, P, see above). Grisebach (1866) cited *Wright 189* as *Miconia lima*; this collection is actually referable to *Miconia cubana*, a Cuban species (see under *Miconia cubana*).

##### Ecology.

*Miconia lima* occurs in moist tropical broadleaf or mixed broadleaf-pine, cloud forests (*Pinus occidentalis*) ranging in elevation from 550–2000 m on limestone-derived soils. The species can be found with *Miconia limoides* in Massif de la Selle, Haiti and Sierra de Baoruco (Monteada Nueva), Dominican Republic and co-occurs with *Miconia paralimoides* in parts of the Cordillera Central, Dominican Republic. *Miconia lima* also occurs with *Calycogonium turbinatum* Urb. & Ekman, *Mecranium septentrionale* Skean, *Meriania involucrata* Naudin, *Miconia capillaris* (Sw.) M.Gómez, *Miconia ferruginea* DC., *Miconia hispidula* (Cogn.) Judd et al. (Loma Isabel de Torres, Dominican Republic), *Miconia dielsiana* Urb., *Miconia jimenezii* Judd & R.S.Beaman, *Miconia punctata* (Desr.) D.Don, *Miconia septentrionalis* Judd & R.Beaman, *Miconia tetrastoma* Naudin, *Miconia lanceolata* DC., *Miconia nanophylla* Judd et al., *Miconia scalpta* (Vent.) Ionta et al., *Miconia subcompressa* Urb. and *Miconia zanonii* Judd et al. See Ciferri (1936) for more information about the species occurrence and associate species in the Cordillera Septentrional (Pico Diego de Ocampo).

##### Conservation status.

*Miconia lima* is the most widespread species of the *Lima* clade on Hispaniola and does not appear to be in any immediate danger from anthropogenic disturbance in most parts of its range. However, localized populations are likely under intense pressure from forest clearing for farming and charcoal production in Haiti (e.g., Massif de la Selle) and the Dominican Republic, so we consider the species as vulnerable.

##### Discussion.

*Miconia lima* forms part of the *Miconia lima* species complex comprised of *Miconia lima*, *Miconia limoides*, *Miconia marigotiana*, *Miconia ottoschmidtii*, *Miconia paralimoides*, *Miconia pedunculata* and *Miconia phyrnosomaderma*. *Miconia lima* is perhaps the most taxonomically complex species of the entire *Lima* clade. The species varies greatly throughout its range but shows geographic cohesion in morphological features, i.e., plants from specific geographic regions can be distinguished from those of other regions. For instance, populations from Massif de la Selle, Haiti tend to have smaller leaves than other populations, and populations in the Cordillera Septentrional, Dominican Republic have longer calyx teeth than most other populations of the species.

Populations in the Monteada Nueva region of the Dominican Republic show potential introgession with *Miconia limoides*, as they have spreading to spreading-ascending stem hairs (instead of strongly ascending stem hairs), strongly angular, bulla-based hairs on the adaxial leaf surface, and dense, bulla-based hairs on the abaxial leaf surface, mostly or nearly obscuring the lamina, as in *Miconia limoides* (e.g., *Howard 12300*, *Judd 5183, 8083*, *Liogier 11661*, *Majure 5960*, *Zanoni 18919, 30470, 38638*; see Fig. 26A–C). *Miconia lima* also potentially hybridizes with *Miconia paralimoides* in the Cordillera Central (see under *Miconia paralimoides*).

Populations of *Miconia lima* from the Loma Quita Espuela region of the Cordillera Septentrional, Dominican Republic are unique within the species in having densely long- shaggy pubescent stems (with hairs to 2.8 mm long), inflorescence axes, and hypanthia, as well as adaxial leaf surfaces with well-developed bulla-based hairs with long, attenuate apices and calyx teeth up to 5 mm long (i.e., *Abbott 2184*, *García 5222*). Partial DNA sequence data from an herbarium specimen (*García 5222*) from this population exhibited synapomorphies with other accessions of *Miconia lima*, and in general, northeasternmost populations of *Miconia lima* in the Domincan Republic tend to have larger leaves and longer hairs on all surfaces of the plant and fall within the morphological limits of Cogniaux’s Ossaea lima var. grandifolia from Loma Isabel de Torres, which we treat as a synonym under a broadly circumscribed *Miconia lima*. Thus, although the Loma Quita Espuela populations are somewhat morphologically divergent from most other populations of *Miconia lima*, we consider them as representing morphologically differentiated, geographically centered populations, which are not clearly diagnosable from others of *Miconia lima*. However, it is certainly possible that more molecular and mophological work on those populations might show them to be distinct from *Miconia lima*.

##### Specimens examined.

**DOMINICAN REPUBLIC: Prov. Baoruco.** Sierra de Neiba, Norte del poblado Apolinar Perdomo cerca del limite con la Prov. San Juan, Monte Bonito; 18°35'N, 71°24'O, 31 Mar 1994, *García 5521* (FLAS, JBSD); Loma del Pavo, Baoruco, 1500 m, 26–27 Jul 1973, *Liogier 19769* (NY). **Prov. Barahona.** La Hotte, between La Cueva and Placer Bonita, alt. 3500–5000 ft, 1 Aug 1950, *Howard 12300* (A, S, US); Sierra de Baoruco, Monteada Nueva, near Polo, 1400–1425 m, 28 May 1986, *Judd 5183* (F, FLAS, JBSD, NY); Monteada Nueva Region, S on Cabral-Polo Road, then 5.9 km SE on “riverbed road” and ca. 0.15 km more on turn-off toward Monteada Nueva (peak is Loma Trocha de Pey), 18°6'51"N, -71°14'5"W, 1330 m, 31 May 2006, *Judd 8083* (FLAS, JBSD); Monteada Nueva, “Cana Brava” S of Cabral Cloud Forest, 1300 m, 15 Jun 1968, *Liogier 11661* (NY); Sierra de Bahoruco, Municipio Polo, Monteada Nueva, lugar denominado Cortico; 18.11419°N, -71.23479°W; 1407 m, 2 Feb 2016, *Majure 5960* (DES, FLAS, JBSD, NY); Sierra de Bahoruco, Prov. Barahona, Municipio Polo, Monteada Nueva, lugar denominado Monte Jo; 18.16822°N, -71.28043°W; 1650 m, 2 Feb 2016, *Majure 5963* (DES, FLAS, JBSD, NY); Sierra de Baoruco, 4 km arriba en el pueblecito rural de Entrada de Cortico, en el camino El Gajo (Monteada Nueva), 18°07.5'N, -71°13.5'W, 4100–4200 ft, 19 Jan 1982, *Zanoni 18919* (FLAS, JBSD, MO, NY); Sierra de Baoruco, en la cima de Morne La Jo; 18°18'N, -71°17'O, 1550–1600 m, 5 Jun 1984, *Zanoni 30470* (FLAS, JBSD, NY); Sierra de Baoruco, Loma “Pie Pol” (Pie de Palo) de La Guásara de Barahona; 18°10'N, -71°12'O, 1250 m, 25 Mar 1987, *Zanoni 38638* (FLAS, JBSD); **Prov. Duarte.** San Francisco de Macoris, 400–1000 m, 5–17 April 1923, *Abbott 2182* (GH, US); San Francisco de Macoris, 400–1000 m, 5–17 April 1922, *Abbott 2184* (NY); Cordillera Septentrional, Reserva Cientifica Loma Quita Espuela, en al lado Noroeste, camino al Valle; 19°21'N, -70°10'O, 600 m, 12 Aug 1993, *García 5222* (FLAS, JBSD, MO). **Prov. Elias Piña.** Cordillera Central, west slope of Loma Nalga de Maco, in clearing called Pinar Claro, along trail from Rio Limpio to the peak, 1650 m, 21 May 1992, *Judd 6587* (FLAS, JBSD, NY); Cordillera Central, Loma Nalga de Maco, entre Pinar Claro y la cima de la loma; 19°13'N, -71°29'O, 1700–1995 m, 21 May 1992, *Santana 956* (FLAS, JBSD). **Prov. Espaillat.** Cordillera Septentrional, Moca, lado N de la Loma El Mogote, 19°29.5'N, -70°29'O, 950 m, 12 Aug 1986, *García 1511* (FLAS, JBSD, MO, NY, S, US); Limestone crest, Jamao, Moca, La Cumbre, 700–800 m, 17 May 1969, *Liogier 15401* (GH, NY, US); Cordillera Septentrional, en la cima de la loma El Magote, aprox. 9 km al NE de Moca; 19°29'N, -70°29'O, 940–960 m, 30 Jul 1986, *Zanoni 36959* (FLAS, JBSD, MO, NY, S); Cordillera Septentrional, al este del Paso, La Cumbre, el paso entre Moca y Jamao al Norte; 19°30'N, -70°30'O, 800 m, 17 Feb 1987, *Zanoni 38327* (FLAS, JBSD, MO, NY, S). **Prov. Hermanas Mirabal.** Cordillera Septentrional, Tenares, Distrito Municipal de Blanco Arriba, paraje La Jibara, en los Mogotes del Peñon, 19°30'56"N, -70°19'55"O, 626 m, 21 Feb 2001, *Veloz 2401* (JBSD). **Prov. Independencia.** Sierra de Neiba, municipio La Descubierta, en la Loma del 15, subiendo por la carretera International, 18°41'N, -71°47'W, 1900–2000 m, 15 Dec 2000, *Clase 2651* (FLAS, JBSD); Sierra de Neiba, La Descubierta, paraje Sabana Real, 18°38'N, -71°48'O, 1800–2000 m, 19 Jul 2002, *Clase 3274* (FLAS, JBSD, MO); Sierra de Neiba, International Hwy., ca. 33.7 km N of La Descubierta on road to Los Doscientos and Hondo Valle, ca. 1800 m, 30 May 1986, *Judd 5194* (F, FLAS, JBSD, MO, S, US); Sierra de Neiba, near La Doscientos, Hondo Valle, 1750–1850 m, 5–7 Sept 1968, *Liogier 12525* (NY); 5–6 km NNW of Angel Feliz, crest and S of crest of Sierra Neiba, 18°41'N, -71°47'W, 15 Oct 1991, Sierra de Neiba, 1500 m, 24–26 Mar 1975, *Liogier 22668* (UCMM); Sierra de Neiba, Municipio La Descubierta, comunidad Sabana Real, yendo desde Loma del 15 hacia La 204, carretera Internacional; 18.68394°N, 71.78865°W; 1901 m, 5 Feb 2016, *Majure 6007* (DES, FLAS, JBSD, NY); Sierra de Bahoruco National Park, El Cielo, 8°07'57"N, -72°40'10.9"W, 1520 m, 2 June 2006, *Skean 4312* (FLAS); 5–6 km NNW of Angel Feliz, crest and S of crest of Sierra de Neiba, 18–41N, -71–47W, 1770–1800 m, 15 Oct 1991, *Thompson 9728* (FLAS); Sierra de Baoruco, en Charco de la Paloma, 37.4 km al sur de Puerto Escondido en el camino a Aceitillar y continuando en el camino a Aguacate, 18°12'N, -71°32'O, 1810 m, 19 Mar 1985, *Zanoni 33924* (FLAS, JBSD, NY); Sierra de Neiba, ladera occidental de la Loma El Hoyazo, entre el Puesto Militar 204 y el Monumento km 204 en la Carretera Internacional (al sur de Aniseto Martinez); 18°41'N, -71°47'O, 1856 m, 15 Jul 1987, *Zanoni 39899* (FLAS, JBSD, MO, NY). **Prov. La Vega.** gorge of Arroyo de la Sal, above the Jimenoa Dam, Jarabacoa, 900 m, 19 Jun 1968, *Liogier 11758* (NY, US); Cordillera Central, Municipio Constanza, Valle Nuevo, en la calle subiendo hacía Pinar Parejo; 18.85540°N, -70.72659°W; 1692 m, 7 Feb 2016, *Majure 6020* (DES, FLAS, JBSD, NY). **Prov. Pedernales.** Sierra de Baoruco, Carretera Internacional, 2024007N, 212375E, 2 Apr 2003, *Clase 3512* (FLAS, JBSD); Sierra de Baoruco, Parque Nacional Sierra de Baoruco, Distrito Municipal José Francisco Peña Gomez, Comunidad Los Arroyos, Carretera International, entrada Ladera del Sur, parche de vegetación justo después del letrero del parque; 18.25557°N, -71.74443°W; 1628 m, 4 Feb 2016, *Majure 5983* (DES, FLAS, JBSD, NY); Sierra de Baoruco, 38 km Sur de Duverge, 0.5 km Sur de Aguacate, en la Carretera Internacional a Los Arroyos y Pedernales, 18°18'N, -71°42.5'O, 1550–1600 m, 18 Aug 1983, *Zanoni 26574* (FLAS, JBSD, NY). **Prov. Puerto Plata.** Cordillera Septentrional, Loma Isabel de Torres, en el casquete de la cima, cara este de la loma; 18°45.2'N, -70°42.5'O, 700–800 m, 23 Sep 1997, *F. Jiménez 2302* (FLAS, JBSD); Loma Isabel de Torres Peak, 775 m, 30 Jun 1963, *J. Jiménez 4778* (FLAS, UCMM, US); Cordillera Septentrional, Pico Diego de Ocampo, above and NW of Piche, 1140 m, 25 May 1986, *Judd 5171* (F, FLAS, JBSD, NY); Cordillera Septentrional, Pico Diego de Ocampo, above and NW of Piche, 1170–1250 m, 25 May 1986, *Judd 5172* (F, FLAS, JBSD, NY, S, US); El Peñon, La Cumbre, Cordillera de Yaroa, 900 m, 2 May 1968, *Liogier 11049* (NY, US); El Peñon, La Cumbre, Cordillera de Yaroa, 2 May 1968, ca. 900 m, *Liogier 11072* (NY); Cordillera de Yaroa, facing the Yaroa Valley, 800–850 m, 11 May 1968, *Liogier 11209* (US); Cordillera de Yaroa, near the trail to Arroyo del Toro, 800–850 m, 28–29 June 1968, *Liogier 11861* (NY, US); Sierra de Yaroa, Arroyo del Toro, 850–900 m, 29 Aug 1968, *Liogier 12389* (NY); Loma Isabel de Torres, 700–800 m, 25 Mar 1969, *Liogier 14582* (GH, NY, US); Cordillera Septentrional, Loma Isabel de Torres, cima de El Teleférico; 19.76345°N, -70.71097°W; 786 m, 10 Feb 2016, *Majure 6036* (DES, FLAS, JBSD, NY); Cordillera Septentrional, Pico Diego de Ocampo, ca. 1200 m, 25 May 1986, *Skean 1804* (FLAS, JBSD); Cordillera Septentrional, Loma Diego de Ocampo, 19°34'47"N, -70°44'42"O, 3 Jul 2000, *Skean 4116* (FLAS, JBSD). **Prov. Puerto Plata/Santiago.** Cordillera Septentrional, Loma Diego de Ocampo, 15 aereo-km Node la ciudad de Santiago; 19°35'N, -70°45'O, 1249 m, 4 Mar 1983, *Zanoni 25575* (FLAS, JBSD, MO, NY). **Prov. San Juan.** La Rucilla, 1800–2000 m, 15 Aug 1968, *Liogier 12144* (NY, US); La Rucilla, Cordillera Central, 1800–2000 m, 15 Aug 1968, *Liogier 12165* (NY). **Prov. San Juan/Azua.** Cordillera Central, Parque Nacional Ramirez, en el sendero entre Los Frios (de Los Montes Frios), 18°53'N, -70°58'O y el Valle de Tetero 18°59'N, -70°65'O, 1400–2100 m, 22 Jun 1988, *Zanoni 41528* (FLAS, JBSD). **Prov. Santiago.** Diego de Ocampo Peak, 1249 m, 1 Jun 2004, *Acevedo-Rodriguez 14224* (US); Cordillera Central, Municipio San Jose de las Matas, Paraje Mata Grande, Parque A. Bermudez, loma del Oro, 22 Apr 1999, *Clase 935* (FLAS, JBSD); Cordillera Central, Municipio de San José de Las Matas, seccion Diferencia, Paraje Diferencia, en Loma de la Guajaca, Parque A. Bermúdez, parte arriba del Rio Amina, 1030 m, 16 Jul 1999, *Clase 1413* (FLAS, JBSD); Cordillera Septentrional, El Cerrazo, ca. 700 m, 20 Feb 1930, *Ekman H14310* (A, NY, S, US); Cordillera Septentrional, Santiago, top of Loma Diego de Ocampo, ca. 1250 m, 19 Jul 1929, *Ekman H13230* (S); Igua, 960 m, 13 Aug 1946, *J. Jiménez 1214* (UCMM, US); Santiago. top of Pico Diego de Ocampo, 1220 m, 3 Sep 1960, *J. Jiménez 4546* (FLAS, UCMM); summit of Pico Diego, Diego de Ocampo (above Pinché), 1220 m, 17 May 1976, *Judd 1510* (A); near summit of Pico Diego de Ocampo, above Pinché, 1175 m, 17 May 1976, *Judd 1528* (A, NY); Loma del Oro, about 5 mi S of Mata Grande, 1200–1400 m, 4 Jun 1968, *Liogier 11529* (NY); slopes of Diego de Ocampo Peak, 1100–1200 m, 17 Sep 1968, *Liogier 12697* (NY); between Guacara and Guacarita rivers, La Guacara Arriba tributary of Bao River, Cordillera Central, 1200–1400 m, 5–9 Nov 1968, *Liogier 13440* (NY); Arroyo del Toro, Tamboril, 800 m, 20 Feb 1965, *Jiménez & Marcano 5132* (NY, UCMM, US); Cordillera Central, 16–17 km al SO de Pedregal de San Jose de las Matas, en Mata Grande en las margenes del Rio Sape Malo al NO de la caseta de la Direccion Nacional al Parques; 19°14'N, -70°58'O, 28 Jun 1988, *Pimentel 1062* (FLAS, JBSD, NY, S, US); Cordillera Septentrional, Loma Diego de Ocampo, 15 aereo-km al NO de la ciudad de Santiago, 19°35'N, -70°45'O, 1249 m, 10 Oct 1987, *Zanoni 40576* (FLAS, JBSD, NY); Cordillera Septentrional, Loma Diego de Ocampo, 13 km por aire al NorO de Santiago, en la ladera del norte, 19°35'N, 70°44.5'O, 1180–1250 m, 7 Jun 1989, *Zanoni 42462* (FLAS, JBSD, NY); Valverde-Santiago. Cordillera Septentrional, sobre Loma El Murazo; 19°41'N, -70°58'O, 1083 m, 18 Dec 1984, *Zanoni 32764* (FLAS, JBSD, NY). **HAITI: Dept. du Centre.** Massif de Cahos, group Las Caobas, Belladére, Morne Lagoune-Ibére, ca. 1350 m, 22 Feb 1926, *Ekman H5620* (S). **Dept. de l’Oeste.** Massif de la Selle, Pétionville, top of Morne Tranchant, 1700–1920 m, 28 Jul 1924, *Ekman H1161* (EHH n.v., GH, NY, S, US); Massif de la Selle, Morne Cabaio, Jardins Bois Pin, 1900 m, 25 Aug 1924, *Ekman H1635* (EHH n.v., IJ, S); Massif de la Selle, Pétionville, Morne Tranchant, ca. 1750 m, 1 Jun 1928, *Ekman H10014* (EHH n.v., NY); Massif de Matheux, l’Arcahaie, Morne Delpech, ca. 1400 m, 14 Nov 1927, *Ekman H9319* (A, IJ, S); [Massif de la Selle], Gros Cheval, Mornes de Commissairés, 1500 m, 9 Aug 1942, *Holdridge 1404* (F, MICH, MO, NY, US); Massif de la Selle, Morne Teleco, 2.8 km NW of Furcy, 2.4 km W of Obléon, 1.5 km SW of Kenskoff, 18.443286°N, -72.294033°W, 1677–1700 m, 18 Jan 2013, *Majure 4334* (EHH, FLAS, NY, US). **Dept. du Sud-Est.** Massif de la Selle, Marigot, Jardins Bois Pin, 1800 m, 25 Aug 1924, *Ekman H1635* (GH, US).

#### 14. Miconia limoides

Taxon classificationPlantaeMyrtalesMelastomataceae

(Urb.) Majure & Judd, J. Bot. Res. Inst. Texas. 7: 269. 2013.

Figs 22F–I , 19D–G

Ossaea limoides Urb., Ark. Bot. 21A(5): 50. 1927. Type: HAITI. Massif de la Selle, Port au Prince, Morne Malanga, ridge of mountain, laubwald, eruptives, 1300 m, 28 Jan 1926, *E L. Ekman H5462* (lectotype: S! [S-R-10029], designated here; isolectotype: K! [K000329547]).
Leandra limoides (Urb.) Judd & Skean, Bull. Florida State Mus., Biol. Sci. 36: 61. 1991. Type: Based on Ossaea limoides Urb.

##### Type.

Based on *Ossaea limoides* Urb.

##### Description.

Evergreen shrub, 1.5–5 m tall; stems round in cross section, not ridged, the internodes 0.3–6.3 cm long, stem indumentum of descending to spreading, bulla-based hairs to 1.6 mm long, these with apices curved upwards; nodal line present. Leaves opposite, decussate, elliptic, 2.1–5.6 × 1.4–3.9 cm, slightly anisophyllous, apex acute, acuminate, to slightly rounded, base acute, cuneate, to rounded, margins dentate to crenulate, dentations (crenulations) covered in a large bulla-based hair, venation acrodromous, 5-veined, the midvein and 2 pairs of arching secondary veins, the outermost sometimes intramarginal, secondary veins mostly basal to suprabasal, the innermost pair suprabasal, produced 1.2–7 mm from leaf base, positioned 0.9–8 mm in from margin at widest point of blade, tertiary veins percurrent, more or less perpendicular to midvein, 1.3–3.2 mm apart at midleaf, intertertiary veins present, tertiary veins often joined by quaternary veins; adaxial leaf surface covered in well developed bulla-based hairs, widest hair bases to 1.8 mm, apices of bulla-based hairs mostly erect to recurved towards the leaf margin, young leaf adaxial surface producing long-stemmed, clavate-dentritic hairs along the primary, secondary, and tertiary veins from between the bulla-based hairs, sessile, glandular hairs produced along the primary, secondary, tertiary, and quaternary veins between the bulla-based hairs; abaxial leaf surface covered in bulla-based hairs, these erect, those along the primary, secondary, and tertiary veins larger than hairs produced throughout the lamina and spreading to descending with the apices recurved upwards, the lamina completely obscured by bulla-based hairs, lamina appearing as a series of pits from depressions of the bulla-based hairs produced from the upper leaf surface and slightly raised intertertiary veins, sessile, glandular hairs produced from between the bulla-based hairs; petioles 0.4–2.2 cm long, covered in descending to spreading, bulla-based hairs on both surfaces, the apices recurved upwards. Inflorescences terminal 3–33 flowered cymes, flowers mostly produced in glomerulate clusters, 0.9–10 × 1.4–5 cm, the peduncle 0.7–3.7 cm long, proximal inflorescence branches 3–15 mm long; bracts oblong to ovate often with an attenuate apex, 1.5–4 mm long; bracteoles narrowly ovate 1.4–3.5 × 0.4–0.8 mm, with an attenuate apex. Flowers 4-merous, sessile or with pedicels to 0.5 mm long; hypanthium 2.7–3.8 mm long, globose, 4-lobed, but lobing mostly obscured by bulla-based hairs, slightly constricted below the torus; free portion of the hypanthium 0.7–1.1 mm long, abaxial surface covered in bulla-based hairs from 1–3 mm long, and occasional, sessile, glandular hairs near the bases of the bulla-based hairs, adaxial surface (i.e., free portion) covered in small, bulla-based hairs; calyx teeth 2.1–3.3 × 0.4–0.8 mm, ascending or spreading, covered in bulla-based hairs; calyx lobes more or less triangular 1.3–2.5 × 1.4–2.6 mm, apex broadly acute, covered in bulla-based hairs abaxially and sessile, sparse, glandular hairs adaxially; calyx tube not tearing, 0.7–1.2 mm long with bulla-based hairs abaxially and sessile, glandular hairs adaxially; petals 4, white to rose colored, elliptic, 4–4.1 × 2.4–2.6 mm, with an acute apex and membranous margin, with one or two slightly bulla-based hairs produced abaxially, just below the apex, to 3.5 mm long; stamens 8; filaments 1.5–2 mm long, glabrous, anthers 1.2–1.4 mm long, with one dorsally oriented pore, anther thecae 1.1–1.3 mm long, anthers without a dorso-basal appendage or with a short appendage to 0.1 mm long; style 4–4.8 mm long, glabrous, not or only slightly dilated in the middle, collar absent, style subtended by a crown of multicellular, bulla-based hairs, which are slightly longer than the surrounding bulla-based hairs of the ovary apex, stigma punctate; ovary 1.5–2.8 × 2.5–4.3 mm, apex flat to convex, pubescent of bulla-based hairs, placentation axile with deeply intruded placenta, 4-locular; berries globose, 4-lobed, purple at maturity, 3.9–7 mm long (including calyx tube), 4.5–9 mm wide, seeds 0.6–0.8 mm long, obpyramidal, testa smooth, light brown, raphe black, smooth, extending the length of the seed.

##### Phenology.

*Miconia limoides* has been collected in flower and fruit from January through August.

##### Distribution

**Figure 23.**
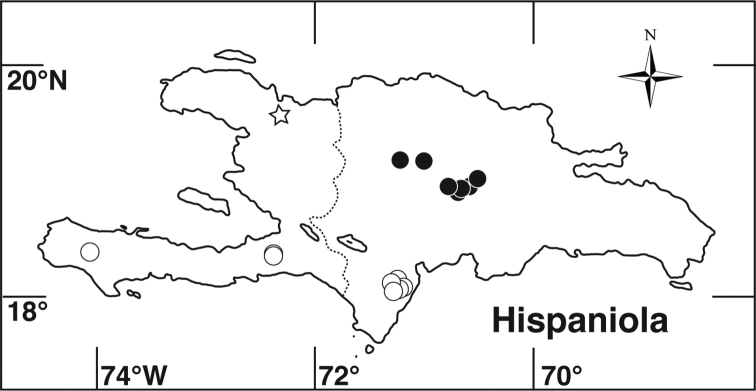
Distribution of *Miconia phyrnosomaderma* (open star), *Miconia limoides* (open circles), and *Miconia paralimoides* (closed circles).

(Fig. 23). *Miconia limoides* only occurs in the southern peninsula of Hispaniola from western Haiti in the Massif de la Hotte (from one collection; *Clase 4132*; see Judd et al. 2015b) to southwestern Dominican Republic in the Sierra de Baoruco (Fig. 19). Only two collections (*Ekman 5462, 9519*) exist from the Massif de la Selle, Haiti from the Port-au-Prince area at Morne Malanga. It is likely that this species occurs more widely in the eastern part of the Massif de la Selle and other parts of Massif de la Hotte.

##### Ecology.

*Miconia limoides* occurs in moist, tropical, broadleaf or mixed broadleaf-pine cloud forests (*Pinus occidentalis*) over limestone-derived soils at elevations ranging from 1100–1400 m. The species is sympatric with *Miconia lima* in both Massif de la Selle, Haiti, and Sierra de Baoruco, Dominican Republic. It also occurs with *Henriettea barkeri* (Urb. & Ekman) Alain, *Henriettea uniflora* Judd et al., *Mecranium ovatum* Cogn, *Meriania involucrata*, *Miconia alainii* Judd & Skean, *Miconia dodecandra*, *Miconia howardiana* Judd et al., *Miconia subcompressa*, *Miconia tetrastoma*, *Miconia umbellata* (Mill.) Judd & Ionta, and *Ossaea gracilis* Alain.

##### Conservation status.

*Miconia limoides* is likely endangered in the Massif de la Selle, Haiti (where it is only known from two Erik Ekman specimens), from habitat loss and the species is not protected in the Sierra de Baoruco of the Dominican Republic, where it is also threatened from anthropogenic disturbance. *Miconia limoides* is only known from one collection in Massif de la Hotte from Parc National Pic Macaya (*Clase 4132*), and the area from where it was collected was heavily disturbed from local farming practices, so we categorize the species as endangered.

##### Discussion.

*Miconia limoides* forms part of the *Miconia lima* complex (Majure and Judd 2013b) and is likely most closely related to *Miconia phrynosomaderma* considering the morphological similarities of descending stem hairs with upward, recurved apices and broadly elliptical leaves. Phylogenetic analyses not including material of *Miconia phrynosomaderma* place *Miconia limoides* as sister to *Miconia lima*, a species with which it is sympatric in parts of its distribution and potentially forms hybrids (see discussion under *Miconia lima*; Fig. 20C–E).

##### Specimens examined.

**DOMINICAN REPUBLIC: Prov. Barahona.** Sierra de Bahoruco, Loma Remigio, en palo doblao del Cachote, 18°05'N, -71°10.5'O, 31 Aug 1999, *F. Jiménez 2992* (FLAS, JBSD); Sierra de Baoruco, Monteada Nueva, near Polo, 1400–1425 m, 28 May 1986, *Judd 5181* (FLAS, JBSD); Sierra de Bahoruco, Monteada Nueva, above (east of) Polo, 1325–1400 m, 18 May 1992, *Judd 6567* (FLAS, JBSD, NY); Monteada Nueva Region (peak is Loma Trocha de Pey), S of Cabral-Polo Rd., then ca. 7.1 km SE on “riverbed road” and dirt road to “Cortico” and silica mine, ca. 0.7 km NNW of silica mine on crest of road, 18°6'38.6"N, -71°13'36.9"W, 1340–1350 m, 31 May 2006, *Judd 8100* (FLAS, JBSD, NY); Sierra de Bahoruco, Prov. Barahona, Municipio Polo, Monteada Nueva, lugar denominado Cortico; 18.11107°N, -71.21983°W; 1370 m, 2 Feb 2016, *Majure 5958* (DES, FLAS, JBSD, NYBG); Sierra de Bahoruco, Prov. Barahona, Municipio Polo, Monteada Nueva, lugar denominado Cortico; 18.11149°N, -71.22688°W; 1428 m, 2 Feb 2016, *Majure 5959* (DES, FLAS, JBSD, NYBG); Sierra de Baoruco, Municipio de Paraiso, en el lugar denominado El Cachote, a 1 km de la caseta de la Sociedad Ecologica de Paraiso; 19267592E 2002124N, 1207 m, 27 Jun 2005, *Veloz 3850* (FLAS, MO); Sierra de Baoruco, 4 km arriba el pueblecito rural de “Entrada de Cortico” en el camino a El Gajo, “Monteada Nueva,” 18°7.5'N, -71°13.5'W, 4100–4200 ft, 19 Jan 1982, *Zanoni 18900* (FLAS, JBSD, NY); Sierra de Baoruco, 7.2 km desde la carretera de Cabral-Polo, en el camino a la Entrada de Cortico y El Gajo (sitio tradicional de Botanicos “Monteada Nuevo”), 18°7.5'N, -71°13.5'O, 4200–4400 ft, 4 May 1982, *Zanoni 20367* (FLAS, GH, JBSD, MO, NY); Sierra de Bahoruco, mas arriba de la Finca Habib, Loma Pie de Pol (Pie Pol), al final de la carretera de La Guasara (de Barahona), 18°10'N, -71°13'O, 1400 m, 19 May 1988, *Zanoni 41131* (FLAS, JBSD); **HAITI: Dept. de l’Oueste.** Massif de la Selle, Crete-á-Piquants, Port-au-Prince, Morne Malanga, 1300 m, 23 Jan 1928, *Ekman H9519* (A, EHH, GH, IJ, NY, S). **Dept. du Sud.** [Massif de la Hotte], Formon, Bois Cavalier, al suroeste de Kay Michel, 18°19'47.3"N, -74°01'38.5"W, 1100 m, 2 Feb 2006, *Clase 4132* (FLAS, JBSD).

#### 15. Miconia phrynosomaderma

Taxon classificationPlantaeMyrtalesMelastomataceae

Majure & Judd, J. Bot. Res. Inst. Texas. 7: 269. 2013.

Fig. 24

##### Type.

HAITI. Dept. du Nord. Massif du Nord, Marmelade, Morne Belle-Terre, 1050 m, fl, fr, 22 May 1927, *E L. Ekman H8204* (holotype: S! [S12-26615]; isotypes: GH!, IJ!, US! [US00775483], EHH n.v.).

##### Description.

Evergreen shrub (height unknown); stems round in cross section, not ridged, the internodes 1–3.2 cm long; stem indumentum of bulla-based hairs 0.4–1.2 mm long, these mixed with some hairs having strongly dilated bases and others only narrowly dilated at the base, the hairs apressed-retrorse along stem or slightly spreading with apices recurved; nodal line present, made up of triangular bulla-based hairs to 2 mm long. Leaves broadly elliptic, 2.4–4.3 × 2–3 cm, often slightly anisophyllous, purplish when young; base rounded to acute; apex broadly acute; venation acrodromous, 5-veined, i.e., with midvein and 2 pairs of arching secondary veins, the innermost pair of secondary veins, mostly symmetrical to subsymmetrical at union with midvein 1.5–5 mm above the leaf base, positioned 2.5–5.5 mm in from margin at widest point of blade, the tertiary veins more or less perpendicular to midvein, 2.4–3.5 mm apart at mid-leaf, tertiary veins sometimes joined by quaternary veins; adaxial surface covered in bulla-based hairs, these not meeting at the base, thus the lamina visible between the hairs, i.e., lamina areoles are not completely filled, widest hair bases to 1.8 mm wide, apices of bulla-based hairs mostly erect to slightly spreading, the young leaf adaxial surface with ephemeral, long-stemmed, clavate-dentritic hairs, these sometimes flattened at the apex, arising from between the bases of bulla-based hairs along the primary and secondary veins toward the base of the leaf, and with subsessile to short stalked glandular hairs along the lamina between bulla-based hairs; abaxial leaf surface covered with bulla-based hairs, although the lamina clearly visible, also with bulla-based hairs covering the primary, secondary, tertiary, and quaternary veins, the lamina covered in sessile glands, also with depressions formed from the bulla-based hairs on the adaxial leaf surface; petiole 0.4–1.2 cm long, covered in bulla-based hairs, these spreading to retrorse and recurved on adaxial surface and mostly appressed-retrorse on abaxial surface. Inflorescences terminal, well-developed to reduced cymes of 3–15 flowers, 1.7–3.9 cm long, 2.2–5.1 cm across, the peduncle 0.1–0.7 cm long, the proximal branches 0.7–1.7 cm long, and pedicels 0.6–1 cm long; bracts narrowly ovate, 2–3 mm long; bracteoles narrowly ovate, 2–2.2 × ca. 0.2 mm, occasionally with bulla-based hairs at base; nodes of inflorescence with mixed bulla-based hairs and long-stemmed, dentritic-clavate hairs, similar to those found at the base of young leaves. Flowers 4-merous; hypanthium 3.1–4 mm long, 5–5.2 mm wide, more or less spherical, slightly 4-lobed, although lobing mostly obscured by bulla-based hairs 0.9–2.5 mm long, the free portion of hypanthium 0.3 mm long, slightly constricted below the torus, both abaxial surface and base of bulla-based hairs with dark, sessile glands, adaxial surface with sessile-glandular hairs; calyx teeth 2.2–3.3 × 0.4–0.7 mm, linear and terete, recurved upon maturation, covered in long, bulla-based hairs; calyx lobes 4 more or less triangular, 1.3 × 1.6 mm, apex acute, with sessile glands near the apex adaxially and bulla-based hairs abaxially; calyx tube 0.4 mm long; petals 4, white, but purplish on the abaxial surface, ovate, 5.1–5.2 × ca. 3 mm, the apex acuminate, margins membranous and entire, clawed at base, with two bulla-based hairs at the apex on the abaxial surface, these 2–3 mm long; stamens 8, the filaments 1.7–1.9 mm long, the anthers 1.4–1.5 mm long, with dorso-basal appendage and a single, dorsally inclined pore, the thecae 1.1 mm long. Style ca. 4.3 mm long, dilated at the center, with punctate stigma, subtended by a crown of long, multicelular hairs, these slightly longer than the surrounding bulla-based hairs on the apex of the ovary; ovary ca. 3.2 × 4.8 mm, apex flat to convex, pubescent with bulla-based hairs. Berries globose, ca. 5 mm long, ca. 6.5 mm wide, blue-black (to purple-black?) at maturity. Seeds (immature) ca. 0.9 mm long, sickle-shaped.

**Figure 24.**
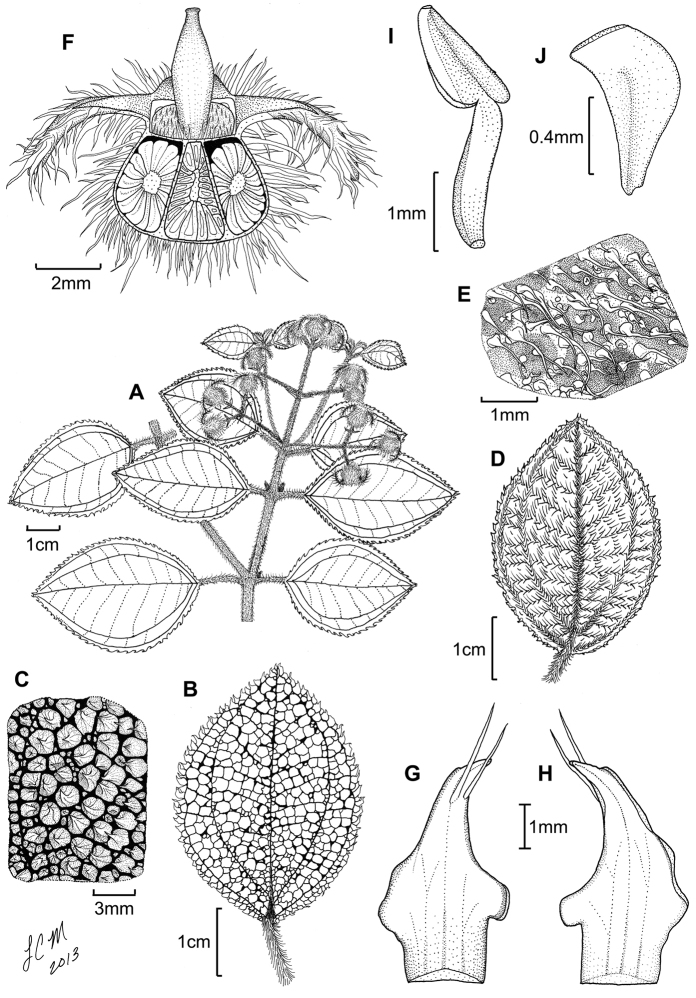
Illustration of *Miconia phrynosomaderma*. **A** habit of *Miconia phrynosomaderma* with the bulla-based hairs removed from the adaxial leaf surface to show venation **B** leaf adaxial surface **C** close-up of adaxial leaf surface showing expanded bulla-based hairs not fully covering the entire lamina **D** leaf abaxial surface **E** close-up of abaxial leaf surface showing sparse bulla-based hairs and clearly visible lamina **F** longitudinal section (slightly oblique) of young fruit **G** petal abaxial surface **H** petal adaxial surface **I** stamen showing anther with short dorso-basal appendage **J** immature seed (all drawn from *Ekman H8204*). Reproduced with permission from Majure and Judd (2013a).

##### Phenology.

This species was collected in flower and fruit in May.

##### Distribution

(Fig. 23). *Miconia phrynosomaderma* is only known from the Massif du Nord, at Morne Belle Terre, Departement du Artibonite, Haiti.

##### Ecology.

This species occurs on metamorphic rock at 1050 m elevation, but no information regarding plant community is available.

##### Conservation status.

*Miconia phrynosomaderma* is likely endangered due to the intense pressure from habitat destruction, as a result of current subsistence farming practices and charcoal production in the Massif du Norde, Haiti. However, there is insufficient data to determine actual conservation status of the species. Thus we categorize the species as data deficient.

##### Discussion.

*Miconia phrynosomaderma* is most likely most closely related to *Miconia limoides* and shares the descending stem hairs with upwardly recurved apices, as well as leaf shape (broadly elliptical; Figs 18, 26D). However, the two species differ in ad- and abaxial leaf surface indumentum, as well as petal base (clawed in *Miconia phrynosomaderma* vs. non-clawed in *Miconia limoides*), calyx teeth length (2.2–3.3 vs. 1.5–2.1 in *Miconia limoides*) and the presence of a dorso-basal anther appendage in *Miconia phrynosomaderma* (to 0.3 mm long vs. only to 0.1 mm long in *Miconia limoides*; Majure and Judd 2013a).

##### Specimens examined.

*Miconia phrynosomaderma* is only known from the type specimen.

#### 16. Miconia marigotiana

Taxon classificationPlantaeMyrtalesMelastomataceae

(Urb. & Ekman) Majure & Judd, J. Bot. Res. Inst. Texas 7: 269. 2013.

Fig. 25

Ossaea marigotiana Urb. & Ekman, Ark. Bot. 22A(17): 65. 1929. Type: HAITI. Sud Est. Massif de la Selle, Marigot, Sd. Bassin Chotard, 9 June 1928, 1750 m, *E.L. Ekman H10071* (lectotype: S! [S-R-10015], designated here; isolectotypes: EHH n.v., G! [G00353949], GH! [GH00112521], NY! [NY00099703], US! [US00775451]).
Leandra marigotiana (Urb. & Ekman) Alain, Sida 18: 1026. 1999. Type: Based on Ossaea marigotiana Urb. & Ekman

##### Type.

Based on *Ossaea marigotiana* Urb. & Ekman

##### Description.

Evergreen shrub, 2–3 m tall; stems round in cross section, not ridged, the internodes 0.9–2.6 cm long, stem indumentum of ascending, appressed, moderately bulla-based hairs to 1.6 mm long; nodal line absent. Leaves opposite, decussate, narrowly elliptic to rhomboid, 2.3–6.6 × 0.9–2.3 cm, slightly anisophyllous, apex acute, base acute to slightly rounded, venation acrodromous, 5-veined, the midvein and 2 pairs of arching secondary veins, the outermost intramarginal, secondary veins mostly basal to suprabasal, the innermost pair suprabasal, produced 2.1–9.3 mm from leaf base, positioned 1.0–2.7 mm in from margin at widest point of blade, tertiary veins percurrent, more or less perpendicular to midvein, 1.0–2.3 mm apart at midleaf, intertertiary veins present, tertiary veins often joined by quaternary veins; adaxial leaf surface densely covered in well developed bulla-based hairs, these covering the leaf areoles, widest hair bases to 1.8 mm, apices of bulla-based hairs mostly recurved toward the leaf margin, young leaf adaxial surface producing occasional long-stemmed, clavate-dentritic hairs along the primary veins towards the leaf base, sessile, glandular hairs produced along the primary, secondary, tertiary, and quaternary veins between the bulla-based hairs; abaxial leaf surface covered in bulla-based hairs, these appressed and ascending along the primary, secondary, and tertiary veins, and erect to spreading on those of along quaternary veins and lamina, those along the primary, secondary, and tertiary veins larger than hairs produced throughout the lamina, the lamina mostly obscured by bulla-based hairs, lamina appearing as a series of pits from depressions of the bulla-based hairs produced from the upper leaf surface and slightly raised intertertiary veins, sessile to short-stalked, glandular hairs produced from between the bulla-based hairs on the lamina; petioles 0.7–1.1 cm long, covered in ascending, appressed, bulla-based hairs on both surfaces. Inflorescences terminal, 9–26 flowered cymes, flowers mostly produced in glomerulate clusters, 2.3–4.1 × 1.0–5.6 cm, the peduncle 0.6–2.5 cm long, proximal inflorescence branches 7–19 mm long; bracts narrowly ovate with an attenuate apex, 1.9–2.1 mm long; bracteoles narrowly ovate, 1.1–1.4 × 0.2–0.22 mm, with an attenuate apex, both bracts and bracteoles only differentiated from bulla-based stem hairs by size, otherwise identical, glabrous or basally with bulla-based hairs. Flowers 5-merous, sessile or with pedicels to 0.3 mm long; hypanthium (immature) ca. 2 mm long, short-oblong, unlobed, slightly constricted below the torus, free portion of the hypanthium ca. 0.5 mm long, abaxial surface covered in bulla-based hairs from 1.1–1.3 mm long, and occasional, sessile, glandular hairs near the bases of the bulla-based hairs; adaxial surface (i.e., free portion) covered in small, bulla-based hairs; calyx teeth 1–1.3 × 0.4–0.5 mm, ascending or spreading, covered in bulla-based hairs; calyx lobes more or less triangular, 0.8–1.2 × 0.5–0.6 mm, apex acute, covered in bulla-based hairs abaxially and sessile, glandular hairs adaxially; calyx tube apparently not tearing, 0.4–0.6 mm long with bulla-based hairs abaxially and sessile, glandular hairs adaxially; petals 4–5, white, ovate, ca. 1.2 × 0.5 mm (immature), with an acute apex and membranous margin, with one large bulla-based hair produced abaxially, just below the apex, to 0.7 mm long; stamens 8–10; filaments (immature) ca. 1.2 mm long, glabrous, anthers (immature) 1–1.1 mm long, with one dorsally oriented pore, anther thecae (immature) 0.9–1 mm long, anthers with a dorso-basal appendage to 0.1 mm long; style ca. 0.9 mm long, glabrous, not dilated in the middle, collar absent, style apparently not subtended by a crown of hairs, stigma punctate; ovary ca. 1.2 × 1.1 mm, apex flat to concave, apparently glabrous, placentation axile with deeply intruded placenta, 2-locular; berries not seen, seeds not seen.

**Figure 25.**
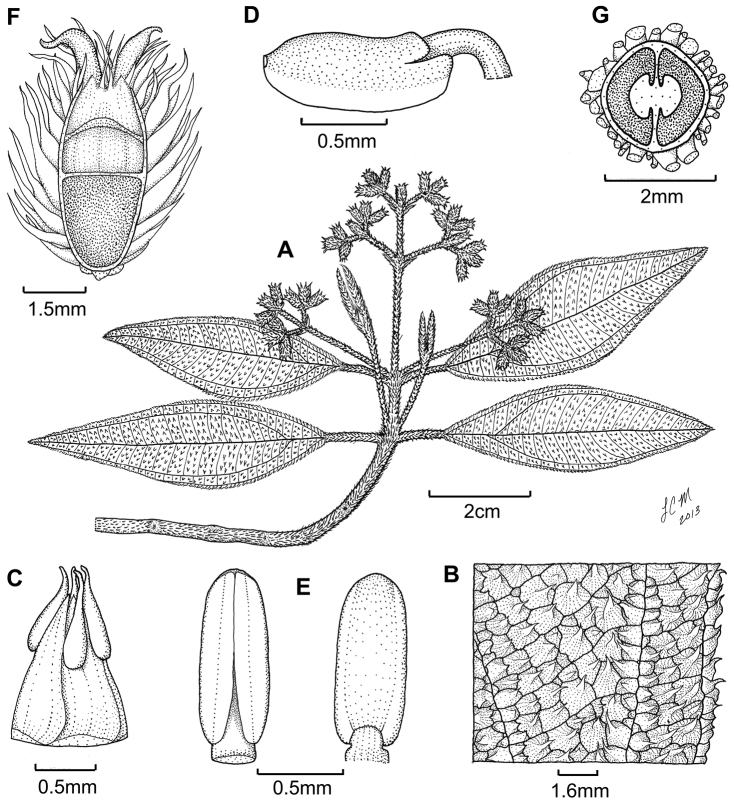
Illustration of *Miconia marigotiana* **A** habit **B** adaxial leaf surface showing well developed bulla-based hairs completely covering areoles **C** petals in bud showing bulla-based hairs on abaxial surface **D** stamen **E** anther ventral and dorsal surfaces **F** immature fruit longitudinal section **G** fruit cross section showing two carpels (all from *Ekman 10071*).

##### Phenology.

Plants have only been collected in June, and these were in bud, and appear to be just before anthesis.

##### Distribution

(Fig. 21). Haiti, Dept. du Sud Est, Massif de la Selle, Marigot, Bassin Chotard.

##### Ecology.

Virtually nothing is known regarding the ecology of this species, but it was collected at 1750 m in elevation, so was likely growing in a moist montane forest over limestone.

##### Conservation status.

*Miconia marigotiana* should be considered endangered, as the part of Massif de la Selle north of Marigot from where the species was collected is under intense pressure from habitat destruction, as a result of current subsistence farming practices and charcoal production. However, we do not have sufficient data to determine the species’ current conservation status and thus consider it data deficient.

##### Discussion.

*Miconia marigotiana* is likely very closely related to *Miconia lima* and *Miconia limoides*, however, it differs from both species in leaf shape, as well as the 2-carpellate ovaries.

##### Specimens examined.

This species is known only from the type gathering by Ekman.

#### 17. Miconia paralimoides

Taxon classificationPlantaeMyrtalesMelastomataceae

Majure & Judd, Phytotaxa 131: 10. 2013.

Figs 26 , 27A–D

##### Type.

DOMINICAN REPUBLIC. Cordillera Central, Provincia La Vega, Constanza, 1.5 hora caminando a pie al sur de Los Mañanguises, en el lugar llamado Sonador, fr. 18°53'N, 70°36'O, 1300 m, 12 Abril 1986, *R. García 1186* (holotype: FLAS! [FLAS179589]; isotypes: JBSD!, MO! [MO-2046086], NY! [NY 01130915], S! [S12-26618], US! [US00775482]).

##### Description.

Evergreen shrub, 1–3 m tall; stems round in cross section, not ridged, the internodes 0.4–8.9 cm long, stem indumentum of bulla-based hairs 0.1–1.3 mm long, these ascending (antrorse) appressed, mostly arcuate, making the stem appear smooth; nodal line present, with larger bulla-based hairs than those on the rest of the stem. Leaves opposite, decussate, broadly elliptic to obovate, 2.7–5.5 × 1.7–3.8 cm, slightly anisophyllous, apex widely acute to rounded, base acute to rounded, venation acrodromous, 5–7-veined, the midvein and 2–3 pairs of arching secondary veins, the outermost intramarginal, secondary veins mostly basal to slightly suprabasal, the innermost pair, more or less asymmetrical at union with midvein, produced 0.3–4 mm from leaf base, positioned 3–7 mm in from margin at widest point of blade, tertiary veins percurrent, more or less perpendicular to midvein, 1.5–2.9 mm apart at midleaf, intertertiary veins present, tertiary veins often joined by quaternary veins; adaxial leaf surface covered in well developed bulla-based hairs completely filling the areoles, bases of bulla-based hairs strongly angular (mostly 4–5 angular) produced from the separation of one hair from another, widest hair bases to 2.2 mm, apices of bulla-based hairs mostly erect, young leaf adaxial surface producing long-stemmed, clavate-dentritic hairs along the primary, secondary, and tertiary veins from between the bulla-based hairs, sessile, glandular hairs produced along the primary, secondary, tertiary, and quaternary veins between the bulla-based hairs, especially toward the base of the leaf; abaxial leaf surface covered in bulla-based hairs, these strongly appressed, those along the primary, secondary, and tertiary veins larger than hairs produced throughout the lamina, the lamina completely covered in bulla-based hairs and thus obscured, lamina appearing as a series of pits from depressions of the bulla-based hairs produced from the upper leaf surface and slightly raised intertertiary veins, sessile, glandular hairs produced from between the bulla-based hairs; petioles 0.4–1.7 cm long, covered in appressed-ascending, bulla-based hairs on both surfaces. Inflorescences terminal, of 5–15 flowered, condensed-short cymes, flowers mostly produced in glomerulate clusters, 0.9–2.8 × 1.2–2.8 cm, the peduncle 0.05–1.5 cm long, proximal inflorescence branches 2–8 mm long, pedicels absent to 1.2 mm long; bracts oblong to ovate, 2.6–4.9 mm long; bracteoles narrowly ovate, 1.7–3.8 × 0.3–0.6 mm. Flowers 4–5(6)-merous, sessile or with pedicels to 1.2 mm long, when 4 or 5-merous, sometimes with one or two calyx teeth apparently aborted; hypanthium 2.9–3.5 mm long, short-oblong to globose, 4-lobed, but lobing mostly obscured by bulla-based hairs, slightly constricted below the torus, free portion of the hypanthium 0.8–1.1 mm long, abaxial surface covered in bulla-based hairs from 0.8–2.6 mm long, and occasional, sessile, glandular hairs near the bases of the bulla-based hairs; adaxial surface (i.e., free portion) covered in small, bulla-based hairs; calyx teeth 2.9–4.2 × 0.8–0.9 mm , ascending or spreading, covered in bulla-based hairs; calyx lobes more or less triangular, 1.3–1.6 × 1.5–2 mm, apex acute, covered in bulla-based hairs abaxially and sessile, sparse, glandular hairs adaxially; calyx tube not tearing, 0.1–0.4 mm long with bulla-based hairs abaxially and sessile, glandular hairs adaxially; petals 4–5(6), red to violet-red, elliptic, 4.5–7 × 2.6–3.6 mm, with an acute apex and membranous margin, with one or two slightly bulla-based hairs produced abaxially, just below the apex, to 2.8 mm long; stamens 8–10(12); filaments 1.8–2.8 mm long, glabrous, anthers 1.5–1.6 mm long, with one dorsally oriented pore, anther thecae 1.2–1.4 mm long, anthers with a dorso-basal appendage 0.18–0.3 mm long; style 5.0–5.3 mm long, glabrous, not or only slightly dilated in the middle, collar absent, style subtended by a crown of multicellular, linear to elongate-triangular (needle-like) hairs, which are slightly longer than the surrounding bulla-based hairs of the ovary apex, stigma punctate; ovary 1.6–2.2 × 2.4–3.4 mm, apex concave, pubescent with bulla-based hairs, except for the linear or elongate-triangular hairs forming crown, placentation axile with deeply intruded placenta, 4-locular; berries globose, slightly 4-lobed, purple (to purple-black) at maturity, 4–10 mm long (including calyx tube), 5.5–9 mm wide, seeds 0.8–1 mm long, obpyramidal, often falcate, testa smooth, light brown, raphe dark brown, smooth, extending the length of the seed.

**Figure 26.**
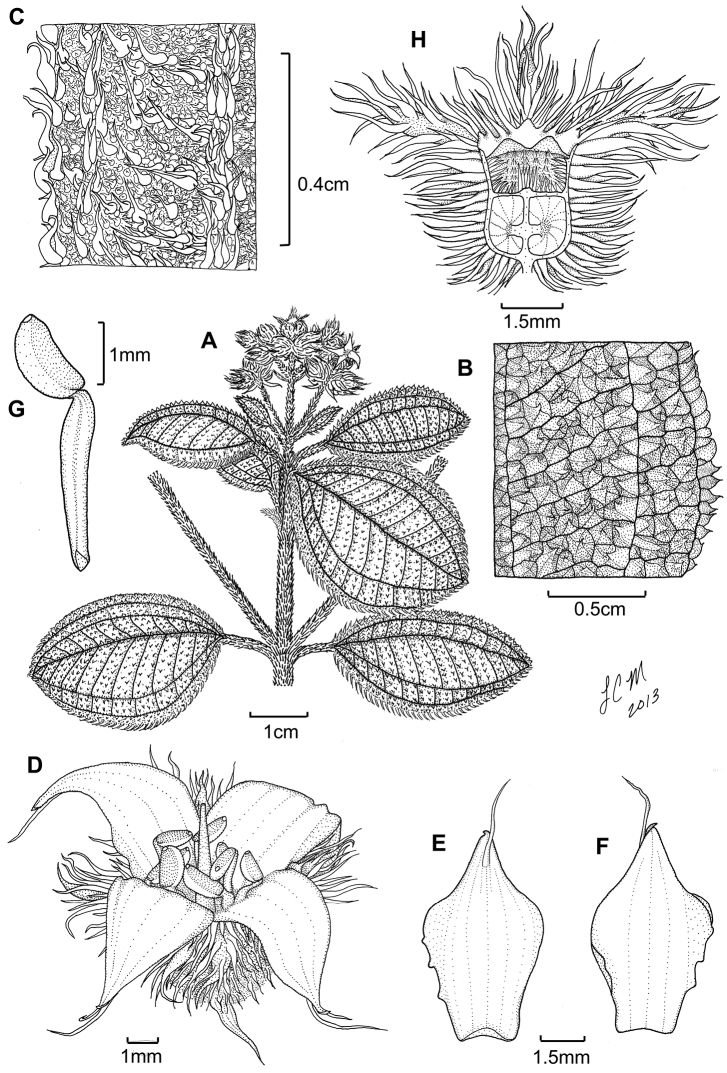
Illustration of *Miconia paralimoides*. **A** habit (*Veloz 4059*) **B** close-up of upper leaf surface (*García 1186*) **C** close-up of lower leaf surface (*García 1186*) **D** flower *Veloz 4059*) **E** petal abaxial surface (*Veloz 4059*) **F** petal adaxial surface (*Veloz 4059*) **G** stamen (*Veloz 4059*) **H** fruit longitudinal section (*F. Jiménez 176–A*). Reproduced with permission from Majure and Judd (2013b).

**Figure 27.**
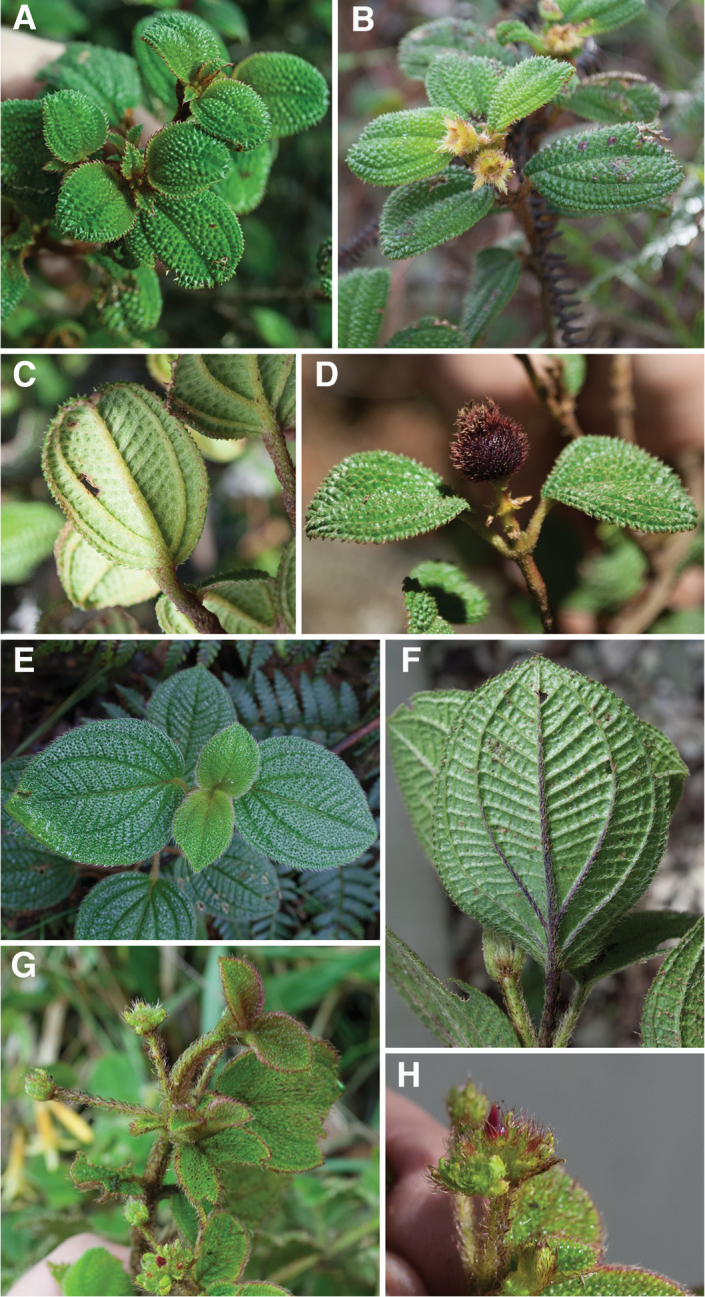
Photos of *Miconia paralimoides* (**A–D**) and *Miconia pedunculata* (**E–H**). **A** habit of *Miconia paralimoides* showing well-developed bulla-based hairs on leaf adaxial surface **B** infructescence of *Miconia paralimoides* **C** leaf abaxial surface showing obscured epidermis and ascending, appressed hairs on petioles **D** mature fruit of *Miconia paralimoides* (all from *Majure 6021*) **E** habit and adaxial leaf surface of *Miconia pedunculata* **F** abaxial leaf surface showing dark purple primary and secondary veins **G** terminal, glomerulate inflorescence of *Miconia pedunculata* showing large bracts subtending floral buds and long, ascending hairs on inflorescence axis and stems **H** reddish-pink floral bud of *Miconia pedunculata* (all from *Majure 6053*). All photos taken by L.C. Majure.

##### Phenology.

*Miconia paralimoides* has been collected in bud, flowering, and in immature fruit from September through April, and has been collected in mature fruit from February through April.

##### Distribution

(Fig. 23). *Miconia paralimoides* is restricted to the Cordillera Central, Dominican Republic.

##### Ecology.

*Miconia paralimoides* occurs in humid, broadleaved mixed forests or pine-dominated cloud forests from 1000-1950 m in elevation. *Miconia paralimoides* occurs with *Miconia pedunculata* and *Miconia lima* in part of its range, although it is unknown if all three species are sympatric in a single area. Other associated melastomes include *Mecranium puberulum* Cogn., *Meriania involucrata*, *Miconia umbellata*, and *Sagraea fuertesii* (Cogn.) Alain.

##### Conservation status.

Certain populations of *Miconia paralimoides* are located inside either national parks or scientific reserves (i.e., Parque Nacional Jose del Carmen Ramirez, Reserva Científica Ébano Verde), however, the species also is under threat from habitat fragmentation and loss outside of those areas. We therefore suggest this species be given a preliminary conservation assessement of vulnerable.

##### Discussion.

Certain populations show potential introgression with *Miconia lima* (i.e., *Clase 1111*, *Liogier 12137, 21719*, *Judd 5165*, *Skean 4134*, *Zanoni 37445, 46740*), due to their shorter hairs, but they have larger fruit than is typical of *Miconia lima* (4.2–4.4 × 5.2–6.2 vs. 2–3.5 × 3–4.1 mm in *Miconia lima*) and appressed bulla-based hairs on the lower leaf surface, as in *Miconia paralimoides*. It is possible that these collections merely represent a short-haired morphotype of *Miconia paralimoides*. Interestingly, Majure and Judd (2013b) considered *Miconia paralimoides* likely closely related to the more phenetically similar *Miconia limoides*, hence the specific epithet. However, our molecular phylogenetic results suggest that *Miconia paralimoides* is sister to the more phenetically divergent *Miconia pedunculata*. There are no clear morphological characters linking these two species, other than those characters typical of the *Lima* clade and the condensed inflorescences of the two species (Figs 20, 24A–E), once again emphasizing the lability of morphological characters in this group.

##### Specimens examined.

**DOMINICAN REPUBLIC: Prov. La Vega.** Cordillera Central, Municipio Constanza, Valle Nuevo, en la calle subiendo hacía Pinar Parejo; 18.84333°N, -70.73644°W; 1944 m, 7 Feb 2016, *Majure 6021* (ASU, DES, FLAS, JBSD, MO, NYBG, USMS); Cordillera Central, Municipio de Constanza, Loma el Paragua, en el lugar denominado el Chiflito, 19°00'06.8'N, -70°43'39.6"O, 1674 m, 10 Nov 2006, *Veloz 4059* (JBSD); Cordillera Central, Municipio Jarabacoa, Distrito Municipal Manabao, Los Tablones, al pie de la Subida de La Cotorra, Parque A. Bermudez; UTM 19Q 301221E, 2107542N, 21 Feb 2011, *Clase 6739* (FLAS, JBSD); Cordillera Central, Reserva Cientifica Ebano Verde, en al camino viejo entre la caseta principal (a la orilla del Arroyo La Sal), y la cima de Loma La Golondrina, 19°04'N, -70°34'O, 1020 m, 12 Mar 1992, *F. Jiménez 176-A* (JBSD); Cordillera Central, Loma La Golondrina, at end of rd to peak of Loma La Golondrina E from Paso Bajito (SE of Jarabacoa), SE of Loma La Sal, 1450–1500 m, 23 May 1986, *Judd 5165* (FLAS, JBSD, NY); Cienaga de la Culata, Constanza, 1600–1700 m, 15–16 Oct 1968, *Liogier 13013* (NY); Loma Redonda, Cienaga de la Culata, Constanza, 1600–1950 m, 23 Sep 1969, *Liogier 15998* (GH, NY, P); Cordillera Central, Parque Nacional Jose del Carmen Ramirez, trail from Los Tablones to La Comparticion; 19°34'51"N, -70°44'42"W, 1980 m, 9 Jul 2000, *Skean 4134* (FLAS, JBSD); Cordillera Central, Reserva Cientifica Ebano Verde, en el camino sendero de Loma El Col a ladera oriental del Río Camu, 19°05'N, -70°33.5'O, 1450 m, 25 Jun 1992, *Zanoni 46740* (FLAS, JBSD, NY); Cordillera Central, Parque Nacional J.A. Bermudez, La Laguna aprox. 3 horas a pie desde La Cienaga (de Manabao) en el sendero al Pico Duarte; 19°02'N, -70°32'O, 2000 m, 13 Jan 1987, *Zanoni 37445* (FLAS, JBSD, MO, NY, US). **Prov. Monte Christi.** Santo Domingo, Cordillera Central, Moncion, Lagunas de Cenobi, Cerro Prieto, ca. 1700 m, 9 Jun 1929, *Ekman H12777* (S). **Prov. San Juan.** slopes of La Rucilla, 1800–2000 m, 15 Aug 1968, *Liogier 12137* (NY, US); La Cotorra, subida a La Rucilla, 1900 m, 15–19 Jun 1974, *Liogier 21719* (NY). **Prov. Santiago Rodríguez.** Cordillera Central, colectada en la subida de La Cotorra, Parque Nacional A. Bermudez, 2110 m, 28 Apr 1999, *Clase 1111* (FLAS); Cordillera Central, Parque Nacional J. C. Ramirez, entre Monte Llano & Los Descansaderos, 19°14'N, -71°17'O, 1300–1400 m, 10 Jul 1988, *Zanoni 41982* (FLAS, JBSD, NY).

#### 18. Miconia pedunculata

Taxon classificationPlantaeMyrtalesMelastomataceae

Majure & Judd, J. Bot. Res. Inst. Texas. 7: 269. 2013.

Figs 22A–E , 27E–H

Ossaea polychaeta Urb. & Ekm,. Ark. Bot. 23A(11): 27. 1931. Type: DOMINICAN REPUBLIC. Cordillera Central, Domingo, [Prov. Monseñor Nouel], Loma la Campana, ca. 1000 m, 2 Feb 1929, *E.L. Ekman H11522* (lectotype: S! [S-R-1003], designated here; isolectotypes: EHH n.v., K! [K000535603], NY! [NY00099709]).
Ossaea urbaniana Alain, Brittonia 20: 158. 1968, nom. illeg. superfl. (nom. nov. for Ossaea polychaeta Urb. & Ekman, non Ossaea polychaete Urb. & Ekman) Type: Based on Ossaea polychaeta Urb. & Ekman
Leandra polychaeta (Urb. & Ekm.) Alain, Sida 18: 1026. 1999. Type: Based on Ossaea polychaeta Urb. & Ekman
Leandra urbaniana (Alain) Alain, Sida 20: 1645. 2003, nom. illeg., later homonym of Leandra urbaniana Cogn. (1886) Type: Based on Ossaea polychaeta Urb. & Ekman

##### Type.

Based on *Ossaea polychaeta* Urb. & Ekman, non *Miconia polychaeta* Wurdack

##### Description.

Evergreen shrub, 1–2 m tall; stems round in cross section, not ridged, the internodes 0.7–8.5 cm long, stem indumentum of relatively sparse, ascending bulla-based hairs to 6.1 mm long, and black, sessile glandular hairs; nodal line present. Leaves opposite, decussate, elliptic, broadly elliptic to almost orbicular, or ovate, 1.4–7.6 × 1–5.9 cm, slightly anisophyllous, apex broadly acute, base rounded to cordate or uncommonly acute, venation acrodromous, 7–9-veined, the midvein and 3–4 pairs of arching secondary veins, veins often purplish on abaxial leaf surface, secondary veins mostly basal, although the innermost pair oftentimes suprabasal, produced 1.5–13 mm from leaf base, positioned 5–14 mm in from margin at widest point of blade, tertiary veins percurrent, more or less perpendicular to midvein, 1.3–4.3 mm apart at midleaf, intertertiary veins absent, tertiary veins sometimes joined by quaternary veins; adaxial leaf surface covered in lateral rows of bulla-based hairs, the rows formed by the production of hairs between tertiary veins, hairs not completely covering lamina areoles, widest hair bases to 1.7 mm, apices of bulla-based hairs mostly recurved, abundant, sessile, glandular hairs produced along the primary, secondary, and tertiary veins, as well as along the bases of bulla-based hairs, these sometimes stipitate along primary and secondary veins; abaxial leaf surface covered in sparse, bulla-based hairs, these restricted to the primary, secondary, tertiary, and quaternary veins, thus the lamina clearly visible, lamina appearing as a series of pits from depressions of the bulla-based hairs produced from the upper leaf surface, sessile, glandular hairs produced along higher and lower order veins, as well as throughout the lamina; petioles 0.4–3.4 cm long, covered in sparse, bulla-based hairs, as well as sessile, glandular hairs (same as the stem). Inflorescences terminal, cymose with flowers forming cymose, glomerulate clusters at the apices of long inflorescence branches making them appear long, pedunculate, 13–17 flowered, 3.2–6.4 × 3.8–5.2 cm, the peduncle absent to 0.5 cm long, inflorescence branches 15–45 mm long, pedicels absent (flowers sessile); bracts foliaceous, mostly ovate to elliptic, 5–10 mm long; bracteoles obovate, 6–7 × 1.6–2.5 mm, ab- and adxial surface covered in bulla-based hairs. Flowers 4–6-merous, sessile; hypanthium 2.6–3.5 mm long, oblong, slightly 4-lobed, slightly to strongly constricted below the torus, free portion of the hypanthium 0.9–1 mm long, abaxial surface covered in bulla-based hairs 4.4 mm long, and abundant, sessile, glandular hairs; adaxial surface (i.e., free portion) covered in bulla-based hairs and sessile, glandular hairs; calyx teeth 3.2–3.6 × 0.3–0.4 mm, mostly spreading, covered in ascending bulla-based hairs; calyx lobes more or less triangular, 1.5–2 × 1.7–2.1 mm, apex rounded, covered in bulla-based and sessile, glandular hairs abaxially and sessile, glandular hairs adaxially; calyx tube not tearing, 1.1–1.3 mm long with bulla-based hairs abaxially and sessile, glandular hairs adaxially; petals 4–5, white or pinkish, narrowly elliptic to oblong, 4.8–5 × 1.9–2 mm, with an acute apex, with one slightly bulla-based hair produced abaxially, just below the apex, to 1.2 mm long; stamens 8–10; filaments 2–2.3 mm long, glabrous, anthers 1.2–1.3 mm long, with one apical to slightly dorsally inclined pore, anther thecae 1.1–1.2 mm long, anthers without a dorso-basal appendage; style 4.9–5.6 mm long, glabrous, dilated in the middle, collar absent, style subtended by a crown of multicellular, triangular hairs, mostly fused at the base, stigma punctate; ovary 1.5–2.4 × 1.1–3 mm, apex flat to concave, glabrous, except for the linear or elongate-triangular hairs forming crown, placentation axile with deeply intruded placenta, 5-locular; berries globose, 4-lobed, purple at maturity, 6.8 mm long (including calyx tube), 7 mm wide, seeds 0.6–0.7 mm long, obpyramidal, biconvex, testa smooth, light brown, raphe light brown, smooth, extending the length of the seed.

##### Phenology.

This species has been collected in flower and fruit from May through December. However, flower buds are present on the type specimen, which was collected in February, as well as on *Majure 6053* also collected in Februrary, so this species likely flowers throughout most of the year.

##### Distribution

(Fig. 21). *Miconia pedunculata* is restricted to the Cordillera Central, Dominican Republic.

##### Ecology.

*Miconia pedunculata* occurs in broadleaved, moist, cloud forests from 1000–2240 m in elevation. Some associate melasomes are *Mecranium puberulum*, *Meriania involucrata*, *Miconia crotonifolia* (Desr.) Judd & Ionta, *Miconia lima*, *Miconia paralimoides*, *Miconia tetrastoma*, *Miconia umbellata*, *Sagraea fuertesii* and *Sagraea oligantha* (Urb.) Alain.

##### Conservation status.

Although this species apparently can tolerate a range of anthropogenic disturbance (according to label data) and also occurs within a scientific reserve in a portion of its distribution, it is of limited distribution in the Cordillera Central, Dominican Republic, and not terribly abundant where it is found. Thus, we assign a preliminary status of vulnerable to *Miconia pedunculata*.

##### Discussion.

The northern populations of *Miconia pedunculata* (e.g., those at the Reserva Científica Ébano Verde) are morphologically differentiated from the southern populations (e.g., from Prov. Peravia), and can be recognized by the less well-developed bulla-based hairs on the adaxial leaf surface, and their more densely pubescent abaxial leaf surfaces. However, the minor morphological differences in these two geographically disjunct groups of populations appear to merely represent variation within a single species. Accessions of *Miconia pedunculata* from both of these regions form a well-supported clade in phylogenetic analyses of the group (Majure et al. 2015; Fig. 2).

Although the name *Ossaea polychaeta* was validly published, Alain Liogier superfluously coined the name *Ossaea urbaniana* Alain (Liogier 1968) as a way of distinghishing *Ossaea polychaeta* (=*Miconia pedunculata*) from *Ossaea polychaete* Urb. & Ekman (=*Miconia polychaete* (Urb. & Ekman) Ionta et al.), a species of the *Sagraea* clade (Ionta et al. 2012) endemic to Massif de la Hotte, what he incorrectly considered to be merely an orthographic variant of the specific epithet. He later transferred *Ossaea polychaeta* to *Leandra polychaeta* (Liogier 1999), and then further transferred *Ossaea urbaniana* to *Leandra urbaniana* (Liogier 2003), creating a later homonym for the already existing *Leandra urbaniana* Cogn. Majure and Judd (2013) coined the name *Miconia pedunculata* for *Ossaea polychaeta*, as the binomial *Miconia polychaeta* Wurdack (Wurdack 1972) already existed for an Andean species from the Cuzco region of Peru.

##### Specimens examined.

**DOMINICAN REPUBLIC: Prov. La Vega.** Cordillera Central: Reserva Cientifica Ébano Verde, 2237 m, 28 May 2003, *Acevedo-Rodriguez 12638* (JBSD); La Vega. sobre Loma Golondrina, 19°03'N, -70°33'O, 1400–1565 m, 29 May 1992, *Zanoni 46124* (FLAS, JBSD, MO, S); Loma de la Sal, SE of Jarabacoa, E of Paso Bajito, ca. 1200 m, 26 May 1992, *Judd 6633* (FLAS, JBSD); Loma de la Sal, SE of Jarabacoa, E of Paso Bajito, 19°04'N, -70°34'O, 1350–1440 m, 27 May 1992, *Zanoni 45942* (FLAS, JBSD, NY); **Prov. Monseñor Nouel.** Cordillera Central, Municipio Constanza, Reserva Científica Ébano Verde, Loma Alto Casabito, en el sendero bajando justo duspués del mirador; 19.04034°N, -70.51817°W; 1398 m, 12 Feb 2016, *Majure 6053* (DES, FLAS, JBSD, NYBG, USMS); Cordillera Central: Reserva Cientifica Ébano Verde), Alto Casibito, along Rt. 12 between Bonao & Constanza at Summit, at the tower/visitor lookout, 19°02'43’’N -70°31'26"W, 1445 m, 1 Jul 2000, *Skean 4103* (ALBC, FLAS, JBSD)(note: label specifies this collection in Prov. La Vega); en la Loma Alto de Casabito, 1/1 km al N del paso del Casabito (cruce Abanico de Bonao-La Palma-El Río de Constanza), 19°2.5'N, -70°31.5'O, 1350–1400 m, 22 Jun 1992, *Zanoni 46527* (FLAS, JBSD)(note: label specifies this collection in Prov. La Vega). **Prov. Peravia.** Loma Valvacoa (Balbacoa), subida por ladera S.E. de la loma desde cañaveral, 18°28'N, 70°20.5'O, 1440–1700 m, 17 Mar 1993, *Jiménez 860* (FLAS, JBSD); El Tope” (la cima) de la Loma Rodríguez, 18°26'N, -70°18'O, 1320–1510 m, 29 Dec 1983, *Zanoni 28291* (JBSD, NY). **Prov. San José de Ocoa.** Cordillera Central, Loma del Rancho, SE de San José de Ocoa, 18°29'N, -70°27.5'O, 1300–1400 m, 19 Aug 1987, *Pimentel 806* (FLAS, JBSD, MO, S); en las cimas de Loma del Rancho, al SE de San José de Ocoa, subida por el lado de “Tumbaca,” 18°31'N, -70°28'O, 1350–1400 m, 14 Aug 1987, *Zanoni 40212* (FLAS, JBSD, MO, NY, US).

#### 19. Miconia pagnolensis

Taxon classificationPlantaeMyrtalesMelastomataceae

Majure & Judd sp. nov.

urn:lsid:ipni.org:names:60473362-2

Fig. 20F

##### Diagnosis.

Species differing from *Miconia lima* in its smaller size (shrub 0.5–0.9 m tall vs. 1.5–5 m tall), smaller leaves (0.9–2.3 × 0.5–1.2 cm vs. 0.7–5.7 × 0.42–3.2 cm) and leaf apex (obtuse to acute in *Miconia pagnolensis* vs. acute to acuminate in *Miconia lima*).

##### Type.

HAITI. Dept. du Sud. Massif de la Hotte, near Morne Bois Pagnol, 12.7 km NE of Duchity, 18.41664°N, -73.77299°W, 1184 m, 20 Jun 2012, *J. Timyan 27* (holotype FLAS! [FLAS260449]).

##### Description.

Evergreen shrub, 0.5–0.9 m tall, stems round in cross section, not ridged, the internodes 0.3–0.67 cm long, stem indumentum of ascending, appressed bulla-based hairs, the longest to 0.6 mm long, nodal line present. Leaves oppostie, decussate, elliptic, small, 0.9–2.3 × 0.5–1.2 cm, base acute, apex obtuse to acute, venation acrodromous, 5-veined, the midvein and two pairs of arching secondary veins, outermost pair of secondary veins basal, intramarginal, innermost pair suprabasal to basal, produced 0.4–1.4 mm from the base, 1.1–2 mm in from the margin at widest point of leaf blade, tertiary veins percurrent, more or less perpendicular to the primary vein, 0.9–1.7 mm apart at midleaf, intertertiary veins obscure but present, tertiary veins often joined by obscure quaternary veins; adaxial leaf surface covered in well developed bulla-based hairs, these completely covering the leaf areoles, the largest 1.6 mm wide at the base, clavate-dendritic hairs prominent in young leaves along the primary, secondary, tertiary and quaternary veins, especially toward the base, abaxial leaf surface covered in bulla-based hairs, those along the primary and secondary veins larger than those along the epidermis and tertiary veins, mostly ascending to appressed, prominent pits formed from the adaxial surface hairs, sessile, glandular hairs throughout the lamina; petioles 1.5–4.5 mm long, covered in ascending, appressed bulla-based hairs. Inflorescences terminal, structure indiscernable (only immature inflorescence seen).

##### Phenology.

Immature inflorescences can be seen on the type specimen collected in late June.

##### Distribution

(Fig. 21). This species is restricted to the Massif de la Hotte, Haiti, and is only known from the type locality.

**Etymology.** The specific epithet, *pagnolensis*, refers to the type locality, Morne Bois Pagnol, and the only place that this species has been collected. It should be noted that there are differing spellings for the type locality. The word is derived from the French, Espagnol (meaning Spanish). The Haitian Creole spelling for the site is Panyòl, although it is also spelled Pangnol and Pagnol (J. Timyan, pers. comm.). We have chosen to use the spelling Pagnol for the site and thus the specific epithet, *pagnolensis*.

##### Ecology.

This species has been collected in a moist, broadleaved forest on karst limestone at around 1200 m in elevation and is associated with *Abarema* sp. (Fabaceae), Brunellia comocladifolia Bonpl. subsp. domingensis Cuatrec. (Brunelliaceae), *Byrsonima* sp. (Malpighiaceae), *Cyathea* sp. (Cyatheaceae), *Schefflera tremulua* (Krug & Urb.) Alain (Araliaceae), *Garrya faydenii* Hook. (Garryaceae), *Prestoea montana* (Graham) G.Nicholson (Arecaceae), and *Wercklea hottensis* (Urb.) Fryxell (Malvaceae).

##### Conservation status.

The conservation status of this species is unknown, however, the location of this collection is surrounded by degraded, remnant moist tropical forest, most of which has been cleared for subsistence farming. Thus, *Miconia pagnolensis* is likely endangered by habitat loss. However, we suggest a preliminary status of data defficient until future fieldwork can further illuminate population sizes of the species.

##### Discussion.

Material of this new species has been collected only once by Joel Timyan (i.e., *Timyan 27*, FLAS). According to phylogenetic analyses (Majure et al. 2015), this species is phylogenetically distinctive and most closely related to members of the *Acuminata* subclade of the *Lima* clade (Fig. 2). *Miconia hybophylla* is the only other putative member of the *Acuminata* subclade in Haiti. Additionally, vegetative morphology certainly places *Miconia pagnolensis* within the *Lima* clade (Fig. 26F). The type specimen shows phenetic similarity with *Miconia lima*, but the shrubs are smaller than *Miconia lima* (0.5–0.9 vs. 1.5–5 m tall), with mostly smaller leaves (0.9–2.3 × 0.5–1.2 cm vs. 0.7–5.7 × 0.42–3.2 cm), with obtuse to acute (vs. acute or acuminate) apices. *Miconia pagnolensis* also occurs outside of the range of *Miconia lima*, which, in the southern peninsula of Haiti, is only known from the Massif de la Selle. We formally name this species, despite its being known only from an essentially vegetative specimen, because our analysis of molecular data clearly indicates the phylogenetic distinctiveness of the taxon, and because its vegetative morphology is diagnostic (allowing it to be distinguished from the phenetically similar *Miconia lima*). The specific epithet refers to the type locality – which is quite inacessable. We think it is important to have a formal name for this rare taxon, as it may be years before it is re-collected (unfortunately, like several other species of sect. Lima, which are only known from the type gathering), and knowledge of its existence will stimulate searches.

#### Names not validly published

*Clidemia hirsuta* Macfad., Fl. Jamaica [Macfadyen] 2: 45. 1850 [?], non *Clidemia hirsuta* (Sw.) Griseb.— manuscript name apparently not effectively published (see Stafleu and Cowan 1981).

## Supplementary Material

XML Treatment for Miconia sect. Lima

XML Treatment for Miconia jashaferi

XML Treatment for Miconia hirtistyla

XML Treatment for Miconia cubacinerea

XML Treatment for Miconia tentaculicapitata

XML Treatment for Miconia norlindii

XML Treatment for Miconia asperifolia

XML Treatment for Miconia hybophylla

XML Treatment for Miconia granulata

XML Treatment for Miconia cubana

XML Treatment for Miconia argentimuricata

XML Treatment for Miconia bullotricha

XML Treatment for Miconia ottoschmidtii

XML Treatment for Miconia lima

XML Treatment for Miconia limoides

XML Treatment for Miconia phrynosomaderma

XML Treatment for Miconia marigotiana

XML Treatment for Miconia paralimoides

XML Treatment for Miconia pedunculata

XML Treatment for Miconia pagnolensis

## Index to numbered collections

Collection numbers are given for each collector with the respective species collected in parentheses. Collections without collection numbers are not included here (ca. 5 gatherings). Although Alain and Liogier both refer to Henri Alain Liogier, we maintain those entries seperately, as Liogier used only the name, Bro. Alain, while collecting in Cuba, but generally used Henri Alain Liogier (or Alain Henri Liogier) while collecting in Hispaniola after he left the Catholic religious order. Collections made by Erik Ekman in Hispaniola are preceeded with an H, as his collection numbers were started anew during his work in Haiti and the Dominican Republic.

Abbott, W.L. 2182, 2184 (*lima*)

Acevedo-Rodriguez, P. 14224 (*lima*); 12638 (*pedunculata*)

Acuña, J. 12633, 13275, 13276, 13277 (*jashaferi*); SV-10189, SV-15144, SV-22705 (*hirtistyla*); 6756, 7714, 9645, 9914, 10193, 13934, 21122 (*norlindii*); 13288 (*granulata*); 7712, 13274 (*argentimuricata*); 9855, 12936 (*ottoschmidtii*)

Acuña, J. & Diaz-Barreto 17520 (*argentimuricata*)

Alain, Bro. 871, 3838, 4726, 4774, 5139, 5489, 7294, 7345 (*jashaferi*)

Alain, Bro. & J. Acuña 7538 (*cubacinerea*)

Álvarez, A. HFC-24074, HFC-55897 (*jashaferi*); HFC-63823, HFC-63699, HFC-64989, HFC-64545, HFC-64791 (*norlindii*); HFC-27126, HFC-27212, HFC-33783, HFC-42557 (*granulata*); HFC-27511, HFC-42530, HFC-64613, HFC-64782, HFC-64999 (*argentimuricata*); HFC-27626 (*bullotricha*); HFC-63793, HFC-64145, HFC-64954, HFC-65636 (*ottoschmidtii*)

Álvarez, A. & R. Berazaín HFC-24386 (*granulata*); HFC-24388 (*argentimuricata*)

Anderson, W.R. & Sternberg 3478 (*asperfolia*)

Arias, I. HFC-58668 (*argentimuricata*)

Bässler HFC-61109 (*argentimuricata*)

Bécquer, E.R. HFC-81644 (*norlindii*); HFC-82434 (*ottoschmidtii*)

Bisse, J. HFC-2668, HFC-3208, HFC-5206, HFC-8179, HFC-11382, HFC-11532, HFC-15391, HFC-19574, HFC-22018, HFC-26994, HFC-22697, HFC-30339, HFC-33804, HFC-37099, HFC-37176, HFC-39514, HFC-45062, HFC-53233 (*jashaferi*); HFC-37347, HFC-37628, HFC-40517 (*hirtistyla*); HFC-21323, HFC-21335, HFC-37324, HFC-40419, HFC-52409 (*norlindii*); HFC-19585, HFC-21469, HFC-33783 (*granulata*); HFC-16787, HFC-19543, HFC-21318,HFC-21332, HFC-21467, HFC-33951, HFC-36916, HFC-37002, HFC-39655, HFC-44528, HFC-49721, HFC-52325, HFC-52629, HFC-52878, HFC-53491(*argentimuricata*); HFC-49731 (*bullotricha*); HFC-37232, HFC-37438, HFC-40386, HFC-40967 (*ottoschmidtii*)

Bisse, J. & Berazaín HFC-21871 (*granulata*)

Bisse, J. & Duek HFC-9337 (*norlindii*)

Bisse, J. & González HFC-25753 (*argentimuricata*)

Bisse, J. & Köhler HFC-5211, HFC-6162, HFC-6739 (*granulata*); HFC-5025, HFC-5197, HFC-6363, HFC-6651, HFC-6772, HFC-8858, HFC-9335, HFC-9455 (*argentimuricata*)

Bisse, J. & H. Lippold HFC-13735, HFC-14699, HFC-18815, HFC-19016, HFC-19091, HFC-19431 (*norlindii*); HFC-11454, HFC-11630, HFC-11640, HFC-11903 (*granulata*); HFC-11144, HFC-13543, HFC-14664, HFC-19017, HFC-19685 (*argentimuricata*); HFC-19643 (*ottoschmidtii*)

Bisse, J. & F.K. Meyer HFC-27230 (*bullotricha*)

Bisse, J. & Rojas HFC-3794 (*norlindii*); HFC-2725, HFC-3242 (*granulata*); HFC-2560 (*argentimuricata*); HFC-8562 (*bullotricha*)

Borhidi, A.121/8 (*granulata*)

Britton, N.L. 3397, 4046 (*asperifolia*)

Christenhusz, M. & Tuomisto 3132 (*asperifolia*)

Chrysogone, Hno. 6374, NSC-5814 (*norlindii*)

Chrysogone, Hno. & Hno. Clemente NSC-3193 (*ottoschmidtii*)

Clase, T. 935, 1413, 2651, 3274, 3512 (*lima*); 4132 (*limoides*); 1111, 6739 (*paralimoides*)

Clemente, Hno. NSC-4056 4706b, 6778 (*jashaferi*); 207, NSC-5814 (*norlindii*); 4705 (*granulata*); NSC-6620 (*ottoschmidtii*)

Dietrich HFC-67133, HFC-67420 (*norlindii*); HFC-45400 (*argentimuricata*)

Eggers, H.F.A. von 3757 (*asperifolia*)

Ekman, E.L. 3849 (*jashaferi*); 14617 (*hirtistyla*); 14823 (*tentaculicapitata*); 7082, 8890, 14343, 14447, 15656, 16076 (*norlindii*); 3702, 14291, 14342, 15927, 15940 (*argentimuricata*); 6926, 8889, 13990, 14258, 15680, 15794, 15912, 16226, 16268 (*ottoschmidtii*); H1161, H1635, H5620, H9319, H10014, H14310, H13230 (*lima*); H9519 (*limoides*); H12777 (*paralimoides*)

García, R. 1511, 5222, 5521 (*lima*)

Greuter, W.R. 25752 (*norlindii*)

Grudzinskaya, J. 764 (*jashaferi*); 759 (*norlindii*); 737 (*argentimuricata*)

Harris, W. 6292, 10682, 10773 (*asperifolia*)

Herrera, P. & N. Imchanitzkaja IMK-429 (*ottoschmidtii*)

Hioram, Hno. 7218 (*tentaculicapitata*)

Holdridge, L. 1404 (*lima*)

Howard, R. 12300 (*lima*)

Ionta, G. 2005 (*asperifolia*)

Jack, J.G. 7011 (*ottoschmidtii*)

Jiménez, F. 2302 (*lima*); 2992 (*limoides*); 176-A (*paralimoides*); 860 (*pedunculata*)

Jiménez, J. 1214, 4546, 4778 (*lima*)

Jiménez, J. & Marcano 5132 (*lima*)

Judd, W.S. 5359, 5443, 5477, 8300, 8337 (*asperifolia*); 1510, 1528, 5171, 5172, 5183, 5194, 6587, 8083 (*lima*); 5181, 6567, 8100 (*limoides*); 5165 (*paralimoides*); 6633 (*pedunculata*)

Jutierrez & Panfet HFC-71754 (*ottoschmidtii*)

León, Hno. LS-22578 (*jashaferi*); LS-10927 (*hirtistyla*); LS-12331 (*tentaculicapitata*); LS-10114, LS-10920 (*norlindii*); LS-22577, 22594 (*granulata*); LS-10416, LS-10948 (*argentimuricata*); LS-7964, LS-10046, LS-10945 (*ottoschmidtii*)

Liogier, A. 11049, 11072, 11209, 11529, 11661, 11758, 11861, 12144, 12165, 12389, 12525, 12697, 13440, 14582, 15401, 19769, 22668 (*lima*); 12137, 13013, 15998, 21719 (*limoides*)

Lippold, H. HFC-16283 (*hirtistyla*); HFC-16088 (*norlindii*); HFC-12491, HFC-12543, HFC-16566 (*argentimuricata*)

Linden, J.J. 2102 (*tentaculicapitata*)

López-Figueiras, M. UO-684, UO-705, UO-2191, UO-2237 (*jashaferi*); UO-430, 2030, 2610, 2657, (*norlindii*); UO-390, UO-396, 2179, 2180, 2613 (*argentimuricata*); 1076, 1421, 2221, 2643, 2692 (*ottoschmidtii*)

Luna, A. 93 (*ottoschmidtii*)

Majure, L.C. 4334, 5960, 5963, 5983, 6007, 6020, 6036 (*lima*); 5958, 5959 (*limoides*); 6021 (*paralimoides*); 6053 (*pedunculata*)

Michelangeli, F.A. 2262, 2284 (*jashaferi*); 2213 (*norlindii*); 2265, 2269 (*granulata*); 2219 (*argentimuricata*); 2228, 2234 (*ottoschmidtii*)

Montero, V. 21295 (*jashaferi*)

Morton, C.V. 9272 (*norlindii*)

Pimentel, J. 1062 (*lima*); 806 (*pedunculata*)

Pipoly, J.J. 24466, 24478, 24522, 24554 (*norlindii*); 24812 (*ottoschmidtii*)

Proctor, G.R. 7620, 10090, 10163, 10192, 23256, 26600, 28226, 28711 (*asperifolia*)

Sánchez HFC-64320 (*ottoschmidtii*)

Santana, J. 956 (*lima*)

Savelev 209 (*ottoschmidtii*)

Shafer, J.A. 4143 (*jashaferi*); 4405, 8006 (*argentimuricata*)

Skean, J.D. 4165 (*jashaferi*); 1869, 1877 (*asperifolia*); 4285 (*argentimuricata*); 1804, 4116, 4312 (*lima*); 4134 (*paralimoides*); 4103 (*pedunculata*)

Stohr HFC-37624 (*ottoschmidtii*)

Stuchlik 771 (*norlindii*)

Thompson, S. 9728 (*lima*)

Tinchanitzkaja, N. 757-758 (*norlindii*)

Veloz, A. 2401 (*lima*); 3850 (*limoides*); 4059 (*paralimoides*)

Wright, C. 1233 (*ottoschmidtii*)

Yeno, M. 1087 (*jashaferi*); 1088 (*granulata*)

Zanoni, T. 18919, 25575, 26574, 20470, 32764, 33924, 36959, 38327, 38638, 39899, 40576, 41528, 42462 (*lima*); 18900, 20367, 41131 (*limoides*); 37445, 41982, 46740 (*paralimoides*); 40212, 45942, 46124, 46527 (*pedunculata*)
